# Advances in Cathode Materials for High-Performance Lithium-Sulfur Batteries

**DOI:** 10.1016/j.isci.2018.07.021

**Published:** 2018-07-26

**Authors:** Chunwei Dong, Wang Gao, Bo Jin, Qing Jiang

**Affiliations:** 1 Key Laboratory of Automobile Materials, Ministry of Education, College of Materials Science and Engineering, Jilin University, Changchun 130022, China

**Keywords:** Inorganic Chemistry, Electrochemical Energy Storage, Energy Materials

## Abstract

Lithium-sulfur batteries (LSBs) represent a promising energy storage technology, and they show potential for next-generation high-energy systems due to their high specific capacity, abundant constitutive resources, non-toxicity, low cost, and environment friendliness. Unlike their ubiquitous lithium-ion battery counterparts, the application of LSBs is challenged by several obstacles, including short cycling life, limited sulfur loading, and severe shuttling effect of polysulfides. To make LSBs a viable technology, it is very important to design and synthesize outstanding cathode materials with novel structures and properties. In this review, we summarize recent progress in designs, preparations, structures, and properties of cathode materials for LSBs, emphasizing binary, ternary, and quaternary sulfur-based composite materials. We especially highlight the utilization of carbons to construct sulfur-based composite materials in this exciting field. An extensive discussion of the emerging challenges and possible future research directions for cathode materials for LSBs is provided.

## Graphical Abstract

**Figure:**
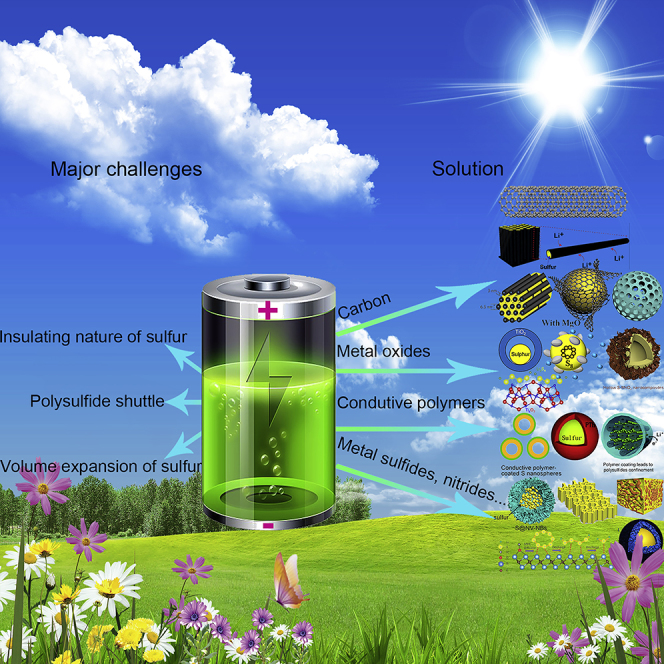

## Introduction

With the rapid development of the modern society, environmental pollution and imminent climate change associated with the use of fossil fuel have attracted considerable attention (Chu et al., 2016). To mitigate these issues and reduce our dependence on fossil fuel, alternative energy technologies based on clean and sustainable energy sources need to be developed, such as solar and wind powers (Seh et al., 2016, Manthiram et al., 2014). However, there exist intrinsic weaknesses for solar and wind powers, such as intermittency and out of control, which lead to significant challenges in efficient and economical electrical energy storage (EES) systems (Wang et al., 2018, Manthiram et al., 2015). Rechargeable battery systems such as nickel metal hydride batteries and lithium-ion batteries (LIBs) have ruled over the electronic market for over a century and are the most viable option for EES (Mahmood et al., 2013, Rehman et al., 2017, Armand and Tarascon, 2008, Chen et al., 2018). Among them, LIBs possess many advantages, such as high energy density, high operating voltage, low self-discharge rate, no memory effects, and long lifetime (Zhou et al., 2016, Wang et al., 2012, Meng et al., 2017). However, expensive LIBs based on the insertion compound-type anode and cathode materials have limited charge/discharge storage capacity and energy density, which cannot satisfy the high-energy demands of electric vehicles, hybrid electric vehicles, and large-scale energy storage devices (Zhang et al., 2016a). New systems with higher energy density and low cost are being intensively explored in the academic and industrial world.

**Figure 1:**
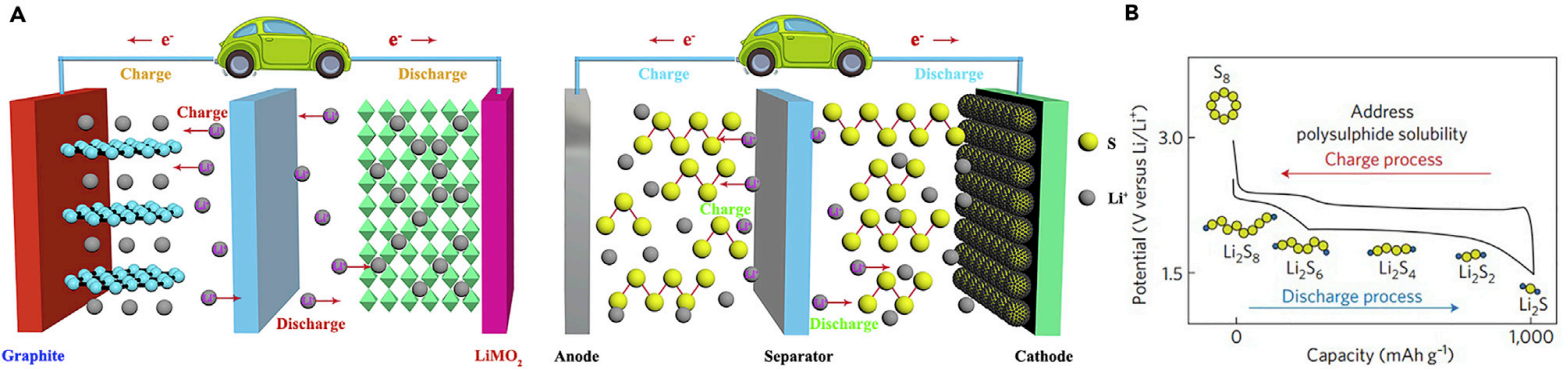
Schematic Representation and Voltage Profiles (A) Schematic representation of LIBs based on the intercalation reaction (left) and LSBs based on the conversion reaction (right). (B) Schematic and voltage profiles of a Li-S battery. Reprinted with permission from (Bruce et al., 2011). Copyright 2012, Nature Publishing Group.

Among the various rechargeable battery systems, lithium-sulfur batteries (LSBs) represent the promising next-generation high-energy power systems and have drawn considerable attention due to their fairly low cost, widespread source, high theoretical specific capacity (1,675 mAh g^−1^), and high energy density (2,600 Wh kg^−1^) (Li et al., 2016e, Manthiram et al., 2017). LSBs were introduced in the early twentieth century and use sulfur as the active cathode material. A comparison of configurations between LIBs and LSBs is shown in Figure 1A. Conventional LSBs generally consist of sulfur-based cathode, binder, separator, organic liquid electrolyte, lithium metal anode, and current collector (Kang et al., 2016). Sulfur as a cathode is naturally abundant, inexpensive, and environment friendly (Zhao et al., 2014c, Su et al., 2016, Mi et al., 2016, Jin et al., 2003, Li et al., 2017b). Metallic lithium as an anode possesses the lowest density and high electron negativity and can deliver a high specific capacity of 3,860 mAh g^−1^ (Liu et al., 2016). During discharge process, Li^+^ ions are produced in the anode and move through the electrolyte to the cathode, whereas the electrons flow through the external circuit, producing Li_2_S as the final discharge product in the cathode. A general illustration of the reaction process of LSBs is shown in Figure 1B (Bruce et al., 2011). Undoubtedly, LSBs as low-cost energy storage devices are particularly attractive for next-generation high-energy power systems.

Despite the overwhelming advantages, such as high specific capacity, high theoretical energy density, and low cost, the commercial utility of LSBs has been gravely hindered by several issues (Wang et al., 2015, He et al., 2018). The main challenges in LSBs can be summarized as follows. First, both sulfur and lithium (di)sulfide discharge product are electronically and ionically insulating, which leads to low electrochemical utilization and limits the rate capability. As a result, carbons, polymers, and metal oxides are required as additives to improve the electrochemical performance of LSBs. Second, the dissolution of intermediate polysulfides generated during cycling results in the notorious shuttling effect, leading to loss of the active material, poor cycling performance, rapid capacity attenuation, and corrosion of lithium metal (Zhang et al., 2012, Hua et al., 2017, Pan et al., 2017). Third, a large volumetric expansion of 80% occurs during the conversion from S to Li_2_S_2_/Li_2_S, leading to structural instability of the electrode and rapid decrease of battery capacity. Fourth, there are limitations associated with using a lithium metal anode. Moreover, customers need to run a 500 km after a single charge. For electric vehicles to fulfill the goal of driving distance exceeding 300 miles (500 km), batteries with a specific energy of ∼500 Wh kg^−1^ are needed. However, today, the specific energy of LSBs achieved is only 160–350 Wh kg^−1^. With the purpose of addressing the aforementioned problems in LSBs, enormous efforts and improvements have been made in the past decades, and can be found in recent review articles.

In this review, the major developments of sulfur/carbons, sulfur/metal oxides, sulfur/conductive polymers, sulfur/metal sulfides, sulfur/metal nitrides, sulfur/metal carbides, sulfur/metal phosphides, organosulfur-based cathode materials, and other ternary and quaternary composite materials will be discussed. Finally, some perspectives and directions on the future development of LSBs are pointed out based on knowledge from the literature and our experiences, which will pave the way for further significant progress in the field of LSBs.

### Sulfur/Carbon Binary Composite Materials

In 2009, Nazar and his team prepared a highly ordered, mesoporous carbon-sulfur composite cathode for LSBs and achieved a major breakthrough. After that, various porous carbon-sulfur composites were investigated, and many research articles have been published on various carbons. In terms of structures, four kinds of carbon materials are discussed in this section, including porous carbons (Strubel et al., 2015, Chung and Manthiram, 2014, Xie et al., 2016, Liang et al., 2016, Chen et al., 2014c, Zhang et al., 2015a, Hou et al., 2016, Zhong et al., 2016, Ren et al., 2016, Li et al., 2016d), graphene or graphene oxide (GO) (Zhou et al., 2017, Tan et al., 2017, Hwa et al., 2017, Kaiser et al., 2017, Chu et al., 2017, Deng et al., 2017, Ma et al., 2017, Xiang et al., 2017, Hao et al., 2017, Qiu et al., 2014), carbon nanotubes (CNTs) (Zhao et al., 2014a, Ye et al., 2016, Hwang et al., 2016, Ahn et al., 2012, Lee and Kim, 2015, Yan et al., 2015, Xi et al., 2015, Zhu et al., 2015, Deng et al., 2016, He et al., 2015), and carbon nanofibers (CNFs) (Zhang et al., 2015b, Zeng et al., 2014, Chung et al., 2015, Singhal et al., 2015).

#### Sulfur/Porous Carbons

Structures of porous carbons are ideal to fulfill different tasks such as improving electronic conductivity, trapping soluble polysulfides contained in electrolyte within the cathode area, minimizing sulfur leaching, and accommodating volumetric change of sulfur during charge and discharge process due to different densities between sulfur and Li_2_S_2_/Li_2_S. Therefore, porous carbon materials represent the most promising group of carbon materials with respect to the practical application of LSBs. Generally, they are amorphous carbon materials with relatively low degree of graphitization but high diversity of pore structures. According to IUPAC definition, pore size is classified as micropore (<2 nm), mesopore (2–50 nm), and macropore (>50 nm). Nanostructured carbon covers all these three categories. Using this criterion, porous carbon can be divided into microporous carbon, mesoporous carbon, and macroporous carbon. Microporous carbons facilitate the immobilization of sulfur and lithium polysulfides due to their confined spaces and thus restrain the shuttle effect to achieve a long cycling stability. Mesoporous carbons not only facilitate the entrapment of sulfur and its reduced species but also provide reasonable pore volumes for high sulfur loading and sufficient ion transport channels for better rate capability. Macroporous carbons within nanoscale permit fast electrolyte ingress and diffusion, which can enhance ion transport kinetics and offer large volumetric space for high sulfur loading. Due to their wide range of microstructures, good conductivities, high specific surface areas, tunable pore sizes, and abundant frameworks, porous carbon materials have been widely used in LSBs for different functions in the anode, cathode, and separator.

Micropores facilitate the immobilization of sulfur and intermediate polysulfides because the small pores confine sulfur and inhibit dissolution of polysulfides formed during the electrochemical reaction (Zhao et al., 2013, Gu et al., 2014). Zhang and co-workers initially used microporous carbon sphere as a sulfur host to improve the long stability of sulfur cathode for LSBs (Zhang et al., 2010). The prepared sulfur-carbon sphere composite cathode demonstrated a long-term cyclability over 500 cycles at a current density of 400 mA g^−1^. The electrochemical reaction was constrained inside the narrow micropores, which was the critical factor for the enhancement of the long electrochemical stability of the sulfur cathode. Moreover, the narrow micropores could restrict the dissolution of lithium polysulfides in organic electrolyte due to strong adsorption. For the sulfur-microporous carbon composite, when the pore size of carbon matrix was around 0.5 nm, this type of cathode system showed better electrochemical performance than the normal S-based electrodes that were realized by Xin et al. (Xin et al., 2012). The confined small S_2−4_ molecules as new cathode materials for LSBs avoided the unfavorable transition between the commonly used large S_8_ and S_4_^2−^. In very recent years, more and more researchers are interested in producing porous carbons by direct carbonization of metal-organic frameworks (MOFs) for a variety of potential applications due to the facile preparation procedures, high carbon yield, and unique porous structures (Xi et al., 2013, Xu et al., 2013). In this regard, Lou's group fabricated microporous carbon polyhedrons (MPCPs) as carbon host matrices to incorporate sulfur for LSBs (Wu et al., 2013). MPCPs/sulfur composite cathode showed a high initial discharge capacity and a stable cycling performance in both 1,3-dioxolane (DOL)/1,2- dimethoxyethane (DME) and ethylene carbonate (EC)/diethyl carbonate (DEC) electrolytes for LSBs.

**Figure 2:**
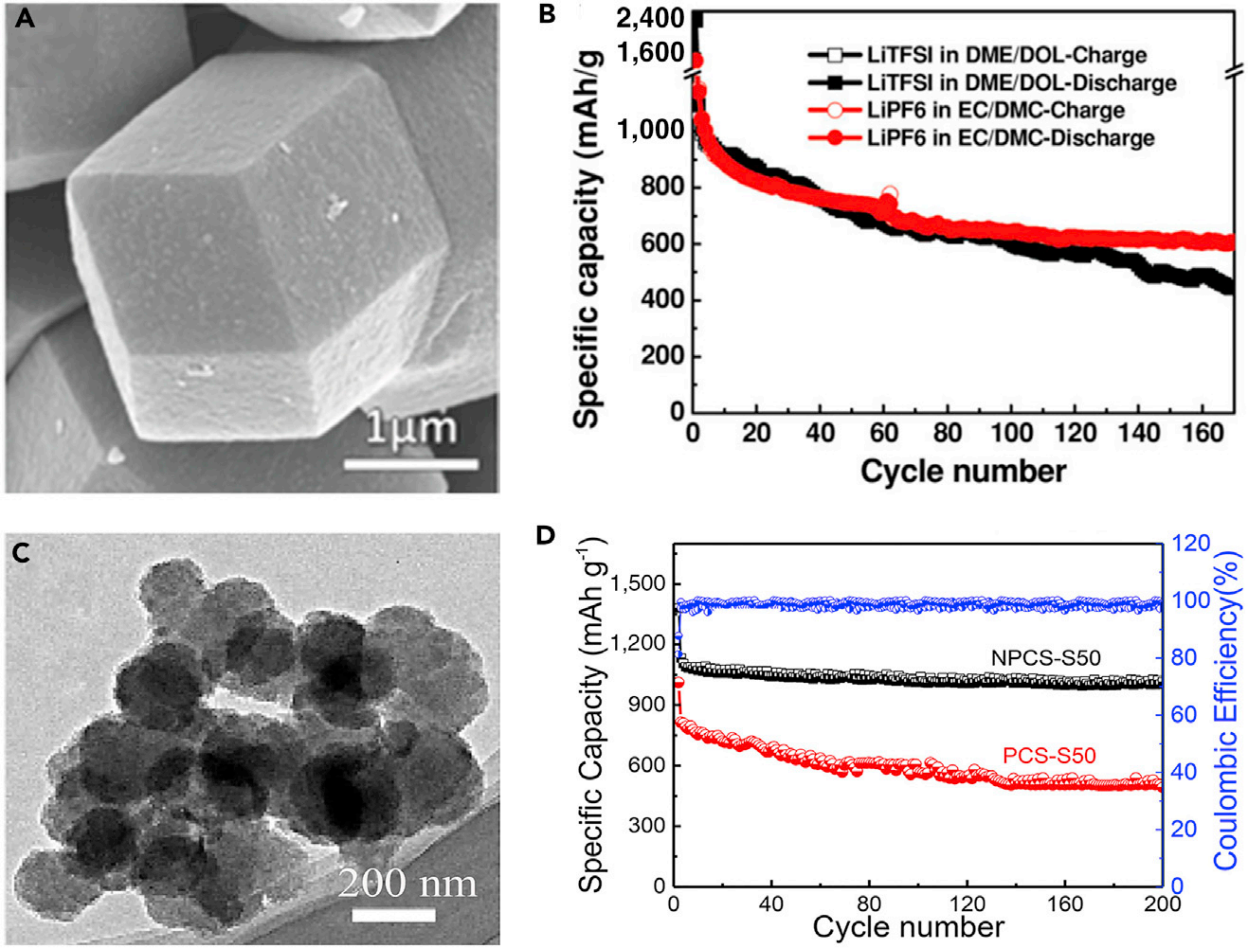
Morphological Characterization and Performance Investigations (A) Scanning electron micrograph of the porous carbon named by Fudan University with rhombic dodecahedral morphology and ordered porous structure. (B) Cycle performance comparison of FDU/S-40 with carbonate-based (EC/DMC) and ether-based (DME/DOL) electrolytes at a current density of 500 mA g^−1^. Reprinted with permission from (Li et al., 2014d). Copyright 2014, Wiley-VCH. (C) TEM image of NPCSs. (D) Cyclic performance and Coulombic efficiency of NPCS-S50 and PCS-S50 hybrids at 0.3 C (1 C = 1,675 mA g^−1^). Reprinted with permission from (Niu et al., 2016). Copyright 2016, Elsevier.

To further understand lithiation/delithiation mechanism and how to select proper electrolytes for S_2–4_ cathodes, Huang and his team prepared S_2–4_ and S_2–-4_/S_8_ composite based on highly ordered microporous carbon as a confining matrix and investigated the electrode mechanism of S_2–4_ cathode in the various electrolytes, combining with theoretical calculation (Li et al., 2014d). The scanning electron micrograph of the as-prepared highly ordered microporous carbon is shown in Figure 2A. It was found that the electrolyte and microstructure of carbon matrix played a significant role in the electrochemical performance of S_2–4_. S_2–4_ confined in the micropores showed excellent cycle stability and good adaptability in carbonate- and ether-based electrolytes (Figure 2B). The lithiation/delithiation reaction of small S_2–4_ molecules confined in the carbon micropores was carried out via a solid-solid mechanism, and this mechanism was beneficial to high Coulombic efficiency. The dissolution of polysulfides and the irreversible reaction between the polysulfides and carbonates were avoided. Moreover, the contact between the polysulfide and the solvent molecule could be stopped by the micropores of carbon matrix. Because of the advantages mentioned above, the battery delivered highly improved electrochemical performance. The element sulfur confined in the micropores could improve the performance of LSBs. In this regard, Peng et al. (Peng et al., 2015) synthesized ultrathin three-dimensional (3D) microporous carbon for LSBs' cathode, which possessed a uniform pore width of approximately 0.6 nm and thickness of approximately 50 nm, by glucose hydrothermal carbonization and self-assembling on GO template. The as-synthesized S@ ultrathin microporous carbon (UMPC) composite was used as a cathode in LSBs, which displayed stable discharge capacity and good rate performance. The outstanding C-rate capacity was attributed to good conductivity of ultrathin porous carbon network and shorter lithium-ion diffusion channel. In addition, the smaller sulfur molecules contributed to enhancing the suppressing effect of intermediate polysulfide product shuttle and fastening lithium-ion transit.

Other than structural modulation, chemical modulation such as heteroatom doping is able to regulate the properties of the microporous carbon significantly as well. For example, nitrogen-doped porous carbon spheres (NPCSs) were designed, prepared, and employed as highly effective sulfur host matrices by simple one-step polymerization and subsequent ZnCl_2_ activation (Niu et al., 2016). The as-prepared NPCSs possess high surface area, large micropore volume, and high nitrogen content. The abundantly doped nitrogen could form strong interaction between Li_2_S_n_ and N atom, suppress the shuttling effect of the soluble polysulfides, and realize the long cycle stability. Figure 2C displays nanostructures of NPCSs. Li-S battery based on the carbon-sulfur hybrid (NPCSs-S) delivered a high reversible capacity of 1,002 mAh g^−1^ at a current rate of 0.3 C after 200 cycles (Figure 2D). It was demonstrated that the nitrogen-doped porous carbon (NPC) could inhibit the dissolution of polysulfides into electrolyte and enhance the reaction kinetics of LSBs. However, microporous carbon only can load a limited amount of sulfur, which will lead to low-energy-density LSBs. To solve this problem, various carbon materials are investigated by researchers.

Admittedly, improvement was achieved by using NPCSs as sulfur host materials. However, the sulfur content is still far below satisfactory, since low sulfur loading will lead to low energy density, which impedes the practical application of LSBs. Mesopores can facilitate the entrapment of sulfur and lithium polysulfide intermediates generated in the electrochemical reaction and offer a reasonable pore volume for a high sulfur loading. Consequently, a large number of studies have focused on mesoporous carbons (He et al., 2011, Schuster et al., 2012, Li et al., 2014a). Early work on disordered mesoporous carbon/sulfur composites was carried out by Wang and co-workers (Wang et al., 2008). Sulfur-coated mesoporous carbon (S-C) composite was synthesized by heating a mixture of elemental sulfur and as-synthesized mesoporous carbon and showed a high initial capacity, but severe capacity fading was observed at a low sulfur loading and the cycle life of 40 cycles needed to be improved as well.

**Figure 3:**
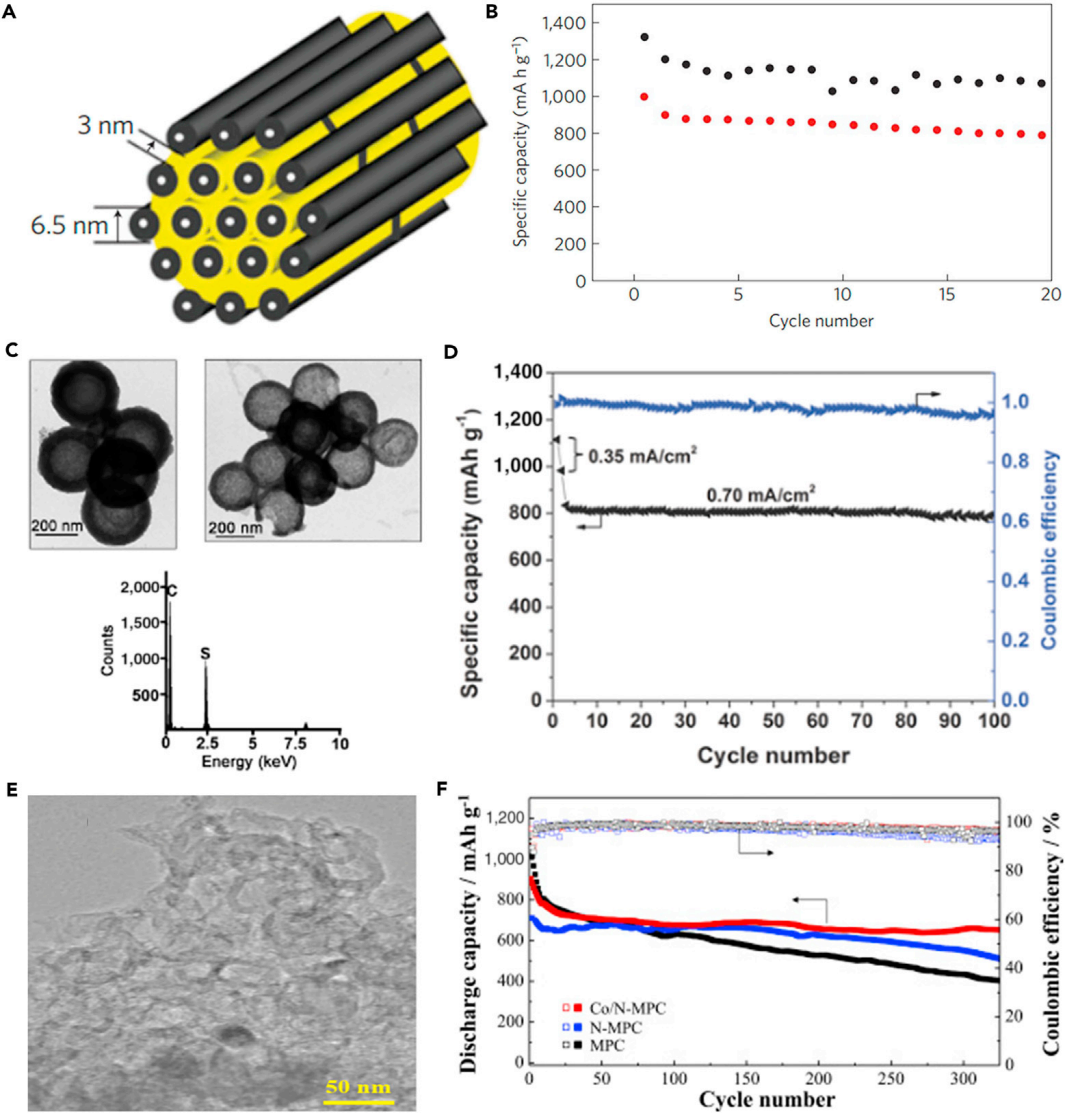
Synthesis Strategies, Morphological Characterization, and Performance Investigations (A) Structural scheme of CMK-3/S composite. (B) Cycling stability comparison of CMK-3/S-PEG (upper points, in black) versus CMK-3/S (lower points, in red). Reprinted with permission from (Ji et al., 2009). Copyright 2009, Nature Publishing Group. (C) TEM images of mesoporous carbon hollow spheres and C@S nanocomposite, and energy-dispersive X-ray (EDX) analysis of C@S nanocomposite showing the presence of sulfur. Reprinted with permission from (Jayaprakash et al., 2011). Copyright 2011, Wiley-VCH. (D) Cycle performance and Coulombic efficiency of MPNC-S70 cycled at a current density of 0.35 mA cm^−2^ for the first two cycles and 0.70 mA cm^−2^ for the subsequent cycles. The capacity values were calculated based on the mass of sulfur. Reprinted with permission from (Song et al., 2014). Copyright 2014, Wiley-VCH. (E) TEM image of Co/N-MPC. (F) Cycling performance of the cells using MPC, N-MPC, and Co/N-MPC for sulfur retention at 1 C and 25°C. Reprinted with permission from (Jin et al., 2016b). Copyright 2016, Elsevier.

In 2009, Nazar's group made a contribution to fabricate sulfur-mesoporous carbon composite, which employed a highly ordered mesoporous carbon (CMK-3) framework to encapsulate sulfur nanofillers within the mesopore channels by a melting diffusion method and generated an intimate electrical contact with the insulating sulfur (Figure 3A) (Ji et al., 2009). The SBA-15 silica was employed as template for preparing the ordered mesoporous carbon (CMK-3). This CMK-3 carbon exhibited uniform and narrow mesopore diameter of 3.33 nm, good conductivity of 0.20 S cm^−1^, and large pore volume of 2.1 cm^3^ g^−1^. The sulfur content in CMK-3-sulfur composite was optimized to 60–70 wt%, which was lower than the theoretical value (79 wt%). The ordered mesoporous carbon could accommodate the volumetric expansion of sulfur nanofillers during the charge/discharge process and provide lithium-ion diffusion channel. CMK-3/sulfur composite displayed a high initial discharge capacity of 1,005 mAh g^−1^ at a current density of 168 mA g^−1^, indicating that the dissolution of polysulfides was inhibited. To further inhibit the diffusion of polysulfides out of the cathode structure and improve the utility of active material, Nazar's group proposed the concept of carbon-sulfur-polymer ternary composite for LSBs, and CMK-3/S composite was coated by a thin-layer polymer of polyethylene glycol (PEG). The thin-layer polymer could further retard the diffusion of noteworthy polysulfides out of the electrode. The polymer-modified CMK-3/S composite possessed an initial reversible capacity as high as 1,320 mAh g^−1^ at 168 mA g^−1^ at room temperature, which was supplied up to nearly 80% of the theoretical capacity of sulfur (Figure 3B). After that, various mesoporous carbon-based flexible sulfur cathodes have been explored; for example, C@S nanocomposites based on mesoporous hollow carbon capsules were synthesized through a facile and scalable method (Jayaprakash et al., 2011). Elemental sulfur was encapsulated and sequestered in the interior of mesoporous hollow carbon capsules (Figure 3C). The carbon capsules could restrain polysulfide dissolution and shuttling effect in the electrolyte, and the porous shell was of benefit to good transport of electrons and provides efficient uptake of elemental sulfur. The as-prepared C@S nanocomposite capsules were used as cathode composites in LSBs and exhibited outstanding electrochemical features at both low and high current densities. Otherwise, the extended scan cyclic voltammetry measurements could confirm the electrochemical stability of C@S nanocomposites. Another facile synthetic method is also adopted for preparing mesoporous carbon; Zhang and his team synthesized highly mesoporous carbon foam (MCF) via a facile, cost-effective, and template-free Pechini method (Tao et al., 2013). The as-prepared MCF possessed a high specific surface area and suitable pore size distribution. MCF/S composite cathode exhibited superior electrochemical performance compared with pure sulfur. Among MCF/S composite cathodes with different sulfur contents, MCF/S (57.22 wt%) cathode delivered the best electrochemical performance with enhanced capacity retention. A stable discharge capacity of 878 mAh g^−1^ was maintained after 50 cycles at 0.05 C. The highly improved electrochemical performance of MCF/S cathode was attributed to the cross-linked hierarchical structure of MCF conductive matrix, which could efficiently trap sulfur and enhance the utilization of active sulfur during the electrochemical reaction, hence relieving the shuttle effect in LSBs. As for other practical applications, MCF can be generalized as well.

Analogous to heteroatom-doped microporous carbon, doped mesoporous carbon also enhances the entrapment of polysulfides and hence is very promising for constructing flexible sulfur-based cathodes. For example, Sun et al. evaluated the effect of nitrogen-doped mesoporous carbon (NC) on the electrochemical properties of LSBs at room temperature and 50°C via using an ionic-liquid electrolyte of 0.5 M lithium bis-(trifluoromethanesulfonyl)imide in methyl propylpyrrolidinium bis(trifluoromethanesulfonyl)imide (Sun et al., 2012). To facilitate the comparison, they also prepared an activated carbon/sulfur (AC/S) composite without nitrogen doping under the same conditions. Because of nitrogen doping, NC/S composite exhibited enhanced activity toward sulfur reduction, higher redox current density, and faster charge-transfer kinetics than the AC/S composite. The corresponding battery displayed a high initial capacity of 1,420 mAh g^−1^ at room temperature under a current density of 84 mA g^−1^ (C/20), which was higher than the battery based on AC/S composite. The discharge potential showed the same tendency as well. After 2 years, a novel mesoporous nitrogen-doped carbon (MPNC)-sulfur nanocomposite as a cathode material for advanced LSBs was prepared (Song et al., 2014). The nitrogen doping was believed to play a significant role in sulfur immobilization that could promote the formation of bonds between sulfur atom and oxygen functional group on the high-surface-area carbon framework. S-O chemical bonding existed in MPNC-sulfur nanocomposite, which was verified by X-ray absorption near-edge structure spectroscopy, and density functional theory (DFT) calculation was conducted to better understand the mechanism. MPNC-sulfur nanocomposite was synthesized by a melting diffusion method, and then employed in LSBs. An excellent Coulombic efficiency of above 96% was achieved, and a capacity retention rate of 95% was obtained within 100 cycles at a current density of 0.70 mA cm^−2^ (Figure 3D). More importantly, a high areal capacity of ∼3.3 mAh cm^−2^ was obtained by using the novel cathode. Even when the content of sulfur was over 4 mg cm^−2^ in the composite electrode, a stable capacity of around 800 mAh g^−1^ still remained. The results mentioned above suggest that the introduction of heteroatoms can lead to a satisfactory enhancement in the chemical adsorption of lithium polysulfides.

More recently, Sun et al. (Sun et al., 2016b) reported that novel mesoporous carbon materials (MCMs) with excellent electronic conductivity and high surface area were synthesized by carbonization of waste litchi shells (LSs) with KOH activation method. The as-prepared MCMs were used as matrices to encapsulate sulfur for LSB cathodes. As a result, MCMs-S composite cathode treated at 300°C exhibited a high initial specific capacity of 1,667 mAh g^−1^ at a current rate of 0.2 C, and a more stable capacity of 612 mAh g^−1^ after 200 cycles at 0.5 C, which was a significant improvement compared with the untreated MCMs-S composite cathode. MCMs possessed excellent electronic conductivity, high surface area, and narrow pore size distribution. The above factors led to excellent electrochemical performance.

Because macroporous carbon has open structure, it cannot effectively encapsulate sulfur and intermediate polysulfides within pores for LSBs. Compared with microporous and mesoporous carbon materials, macroporous carbon materials as cathode hosts of LSBs were less investigated. Waste LSs were used as the carbon source to prepare porous activated carbons through KOH activation method (Zhang et al., 2014a). This porous activated carbon possessed an ultrahigh specific surface area of 3,164 m^2^ g^−1^ and a large pore volume of 1.88 cm^3^ g^−1^, and these two factors were beneficial to enhance the sulfur content and ensure highly dispersed elemental sulfur in the carbon/sulfur composite. Therefore, the utilization of active material was enhanced, which was reflected by the enhanced capacity and prolonged cycling life. The activation litchi shells (a-LSs)/sulfur composite was prepared via a melting diffusion approach, and employed in LSBs. The battery based on the resulting AC/S composite cathode with 60 wt% sulfur content possessed a low fade rate of 0.06% per cycle over 800 cycles at a current density of 800 mA g^−1^. In addition, after LiNO_3_ was added in the electrolyte, the Coulombic efficiency of AC/S composite electrode was enhanced.

In a further study, a modified macroporous carbon (mMPC) containing Co-N_x_ site was synthesized for the first time (Jin et al., 2016b). Figure 3E shows transmission electron microscopic (TEM) image of Co/N-MPC. Compared with MPC and N-MPC, Co/N-MPC possessed stronger adsorption capability. Various nitrogen sites were created simultaneously during macropore formation, such as graphitic-N, pyrrolic-N, pyridinic-N, pyridinic-N oxide, and Co-N_x_. Owing to the synergy effect of nucleophilic N and electrophilic Co, mMPC-containing Co-N_x_ site showed excellent polysulfide adsorption capability. The corresponding Li-S battery assembled with Co/N-MPC exhibited excellent cyclability, and a rate capacity of 660 mAh g^−1^ was maintained after 300 cycles at a higher C-rate of 1 C (Figure 3F).

#### Sulfur/Graphene

As a two-dimensional (2D) carbon representative, graphene can be synthesized from graphite and inherently shows many advantages, such as high surface area (2,600 m^2^ g^−1^), excellent electrical conductivity (∼10^6^ S cm^−1^), lightweight, good flexibility, superior mechanical strength, and good chemical inertness (Fang et al., 2016, Zhou et al., 2013a). Considering these physical properties, graphene has risen abruptly in the electrochemical energy storage devices in recent years (Zhou et al., 2014a, Song et al., 2016, Yu et al., 2016), and especially is used as a favorable substrate for loading active materials like sulfur. Graphene is composed of a one-atom-thick 2D planar carbon sheet, and the sulfur particles are loaded on the surface of 2D nanosheets. Lithium polysulfides are also adsorbed on the side of cathode, which relies on the functional groups on the surface of graphene to some extent. Moreover, the surface of graphene can be decorated with various functional groups, such as carboxyl and hydroxyl groups. The doped graphene used as a host matrix for LSBs can form a strong carbon-sulfur chemical bonding to anchor sulfur particles and intermediate lithium polysulfides. Very recently, many researches have focused on fabricating sulfur-graphene composite cathodes for LSBs.

In 2011, Dai and co-workers synthesized a graphene-sulfur composite material as a cathode for LSBs (Wang et al., 2011). The composite material was prepared by synthesizing submicrometer sulfur particles coated with PEG-containing surfactants and graphene sheets. The graphene wrapping not only trapped dissolvable polysulfide intermediates generated in the electrochemical reaction but also improved the overall electronic conductivity. PEG coating layers partly accommodated volumetric variation of the sulfur particles during the electrochemical process. Consequently, the battery based on graphene-sulfur composite exhibited high and stable specific capacity. In the same year, Zhang and his team used a low-cost and environment-friendly chemical approach to immobilize sulfur through the reactive functional groups on GO (Ji et al., 2011b). The functional groups on the GO surface could effectively immobilize sulfur and lithium polysulfides, and thus GO-S nanocomposite showed a high specific capacity after 50 cycles at 0.1 C when used as a cathode material for LSBs. Further improvement was achieved by using a novel composite structure of high-performance sulfur-graphene. With the aim to produce the graphene-sulfur composites and freestanding low-defect graphene sheets, Lin et al. (Lin et al., 2013) developed a new sulfur-assisted exfoliation of graphite. The van der Waals force among adjacent π-π stacked graphene layers was weaker than the interaction between sulfur and graphene due to the similar electronegativities of the two elements. The as-prepared graphene sheet possessed a high electrical conductivity of 1,820 S cm^−1^ and a high Hall mobility of 200 cm^2^ V^−1^ s^−1^, which was beneficial to homogenously disperse sulfur, anchor sulfur, and enhance the electronic conductivity of insulating sulfur. The battery assembled with the unique structure exhibited excellent electrochemical behavior at higher C-rates of 1 and 2 C.

**Figure 4:**
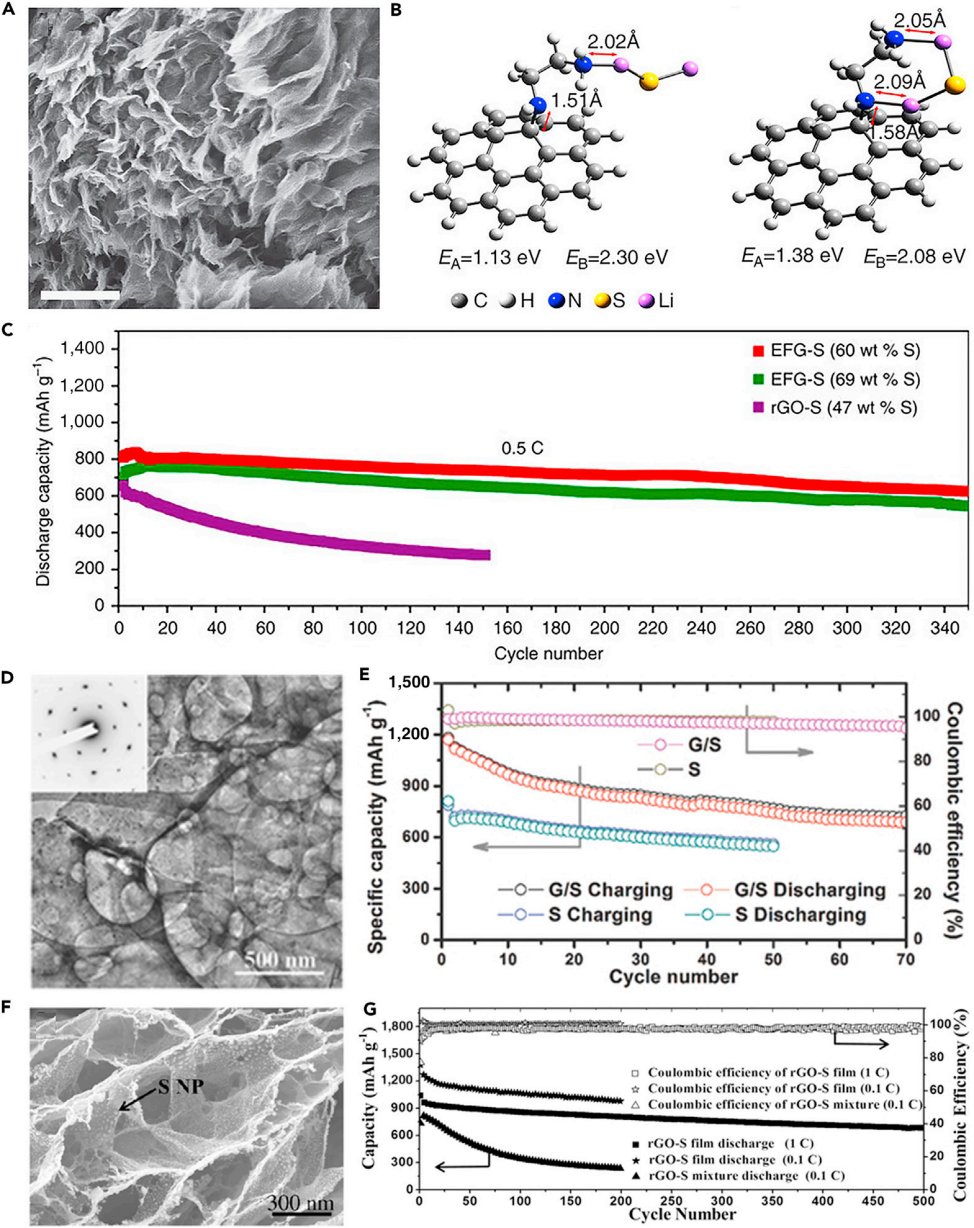
Morphological Characterization, DFT Calculation, and Performance Investigations (A) Scanning electron micrograph (scale bar, 500 nm) of EFG-S nanocomposite. (B) DFT calculation showing the interaction between Li_2_S cluster and EFG. (C) Long-term cyclability of EFG-S nanocomposite (60 and 69 wt% S) and rGO-S composite (47 wt.% S). Reprinted with permission from (Wang et al., 2014b). Copyright 2014, Nature Publishing Group. (D) TEM image of G/S nanosheets. (E) Cyclic performance and Coulombic efficiency of G/S cathode at 0.5 C, compared with pure S cathode. Reprinted with permission from (Fei et al., 2015). Copyright 2015, Wiley-VCH. (F) Cross-sectional scanning electron micrograph of rGO-S composite film at high magnification. (G) Cycling performance and Coulombic efficiency of rGO-S mixture and rGO-S composite film electrodes at 0.1 C for 200 cycles and long-term cycle stability of rGO-S composite film electrode at 1 C for 500 cycles. Reprinted with permission from (Cao et al., 2016b). Copyright 2016, Wiley-VCH.

To understand the mechanism of discharge products and further improve the electrochemical performance of LSBs, a high-performance sulfur-carbon cathode was prepared via covalently stabilizing the sulfur (Wang et al., 2014b). The scanning electron micrograph of ethylenediamine (EDA)-functionalized reduced graphene oxide (rGO) (EFG)-S nanocomposite is shown in Figure 4A. The strong affinity of lithium sulfides to EFG was demonstrated by experimental measurement and theoretical calculation. DFT calculation demonstrated the high binding energy of 1.13–1.38 eV between them (Figure 4B). The unique molecular structure of EDA with high reactivity was beneficial to covalently join polar lithium sulfides and the nonpolar carbon surface together, and hence enhance the utility of active material. The rGO framework could improve the conductivity and mechanical stability of nanocomposite by reducing electrically insulating GO to conductive rGO. Using EFG and sulfur structure as cathode material delivered excellent cyclability at a current rate of 0.5 C (Figure 4C). Many other groups also employed GO to improve the cycling performance of LSBs. For example, Fei et al. (Fei et al., 2015) prepared graphene/sulfur (G/S) hybrid nanosheets via redox reaction between GO and H_2_S. A space-confined “sauna” system was introduced into the redox reaction for the first time. The advantage of novel space-confined “sauna” reaction was verified by experimental measurement and theoretical calculation. A uniform layer of S was anchored onto the surface of graphene (Figure 4D). The charge and ion transfer kinetics of G/S nanocomposites were enhanced, which was attributed to the highly conductive graphene and the uniform distribution of sulfur on graphene. The strong chemical bonding on G/S interface could inhibit the dissolution of polysulfides into electrolyte, enhance the utilization of active material, and thus improve the electrochemical performance of G/S nanocomposite as a cathode composite for LSBs. The battery based on G/S hybrid nanosheets showed a high specific capacity of 700 mAh g^−1^ after 70 cycles at 0.5 C, and a high Coulombic efficiency of >96% was obtained as well (Figure 4E), implying that the dissolution of polysulfides and loss of active sulfur were suppressed.

Recently, Cao et al. (Cao et al., 2016b) fabricated freestanding nanostructured rGO-S composite film by synchronously reducing and assembling GO sheets with S nanoparticles on a metal surface. The scanning electron micrograph of rGO-S composite film is shown in Figure 4F. This composite film could effectively inhibit the diffusion of lithium polysulfides in organic electrolyte while acting as a physical barrier to trap polysulfides, and provide a fast way of electron and ion transport. Moreover, the metal current collector (e.g., Al foil), conductive agent, and binder are inactive components in the electrode, which offsets the merits of high-energy LSBs. However, the fabrication of freestanding binder-free rGO-S film electrode can avoid this disadvantage dramatically. With all these advantages, rGO-S composite film delivered excellent electrochemical performance. As shown in Figure 4G, the structure delivered a high initial discharge capacity of 1,302 mAh g^−1^, and more importantly, it was able to maintain a stable cycling performance for 200 charge/discharge cycles at 0.1 C. Because of the excellent conductive and mechanical properties of rGO-S composite film, they could be prepared into different shapes to be used as cathode materials for flexible LSBs.

#### Sulfur/Carbon Nanotubes

Since their discovery in 1991, CNTs have drawn much attention as ideal materials for considerable applications. They can be divided into two categories: single-walled carbon nanotubes (SWCNTs) and multi-walled carbon nanotubes (MWCNTs). One-dimensional (1D) CNTs have many advantages, such as scalable synthesis, low production cost, excellent mechanical properties, and high electrical and thermal conductivities. Hence, CNTs have been widely employed as suitable matrices to encapsulate sulfur for LSBs. Moreover, CNTs can form the 3D conductive network to maintain the structural integrity of cathode during charging-discharging process. It can also offer large void in the materials to accommodate the volumetric change during cycle, lead to good cycle stability, and provide fast electron conductive pathways. In recent years, various sulfur-carbon composites have been designed to overcome crucial difficulties in LSBs (Ai et al., 2016, Jin et al., 2017, Ma et al., 2014c). In 2003, 1D CNTs were used as the inactive additive materials in LSBs by Lee and co-workers (Han et al., 2003). MWCNTs (20 wt%) were added to the sulfur cathode, which resulted in an increase in the specific capacity (∼400 mAh g^−1^ without additive versus 485 mAh g^−1^ with additive) and improved the adsorption ability of lithium polysulfides. Therefore, a high capacity retention was also obtained after 50 cycles after the addition of MWCNTs into the pristine sulfur cathode. It represented a promising way to inhibit polysulfide dissolution. However, the capacity decay over 50 cycles was observed in both cases (with and without additive), which might result from the dissolution of intermediate polysulfide products into the electrolyte. Moreover, the traditional slurry mixing method was hard to guarantee sufficient contact between sulfur and CNTs. With the purpose of creating a more intimate contact and hence inhibiting polysulfide dissolution, a sulfur-coated MWCNT composite material (S-coated MWCNTs) was prepared via a facile heating treatment (Yuan et al., 2009). The cycle life of the sulfur cathode was improved, and a reversible capacity of 670 mAh g^−1^ was maintained after 60 cycles. The enhanced cycle ability was attributed to the introduction of MWCNTs and the homogeneous distribution of MWCNTs in the composite cathode.

Before 2011, sulfur was mainly coated on the outer surface of MWCNTs; it resulted in the diffusion of lithium polysulfides into organic electrolyte and thus decreased the utilization of active substance during the electrochemical reaction. To achieve higher cycle stability and efficiency, sulfur-impregnated disordered CNT (SDCNT) composite cathode material for LSBs was synthesized by Wang's group (Guo et al., 2011). The sulfur impregnation was through heat treatment of SDCNTs under three different temperatures 160, 300, and 500°C in vacuum-sealed quartz tubes. Among all three samples, SDCNT-500 cathode showed excellent performance, and a capacity retention of 72.9% was achieved after 100 cycles except the first one. After 30 cycles, the capacity of SDCNT-500 composite cathode remained unchanged. Meanwhile, a high Coulombic efficiency of 96% was maintained at 0.25 C during 100 cycles. The superior cyclability and Coulombic efficiency were believed to arise from high-temperature heat treatment of SDCNTs in a vacuum environment. S_8_ molecule was broken down to S_6_ or S_2_ by heat treatment to form sulfur-carbon bonding, and the conventional Li-S_8_ reaction with dissolvable polysulfides would be changed. There were some graphitized carbon layers and smaller voids/defects in amorphous carbon, and the vaporized small sulfur molecules could be incorporated, and hence liquid electrolyte could not directly access and avoid polysulfide dissolution. With all the advantages mentioned above, the SDCNTs demonstrated enhanced electrochemical properties as cathode materials for LSBs.

**Figure 5:**
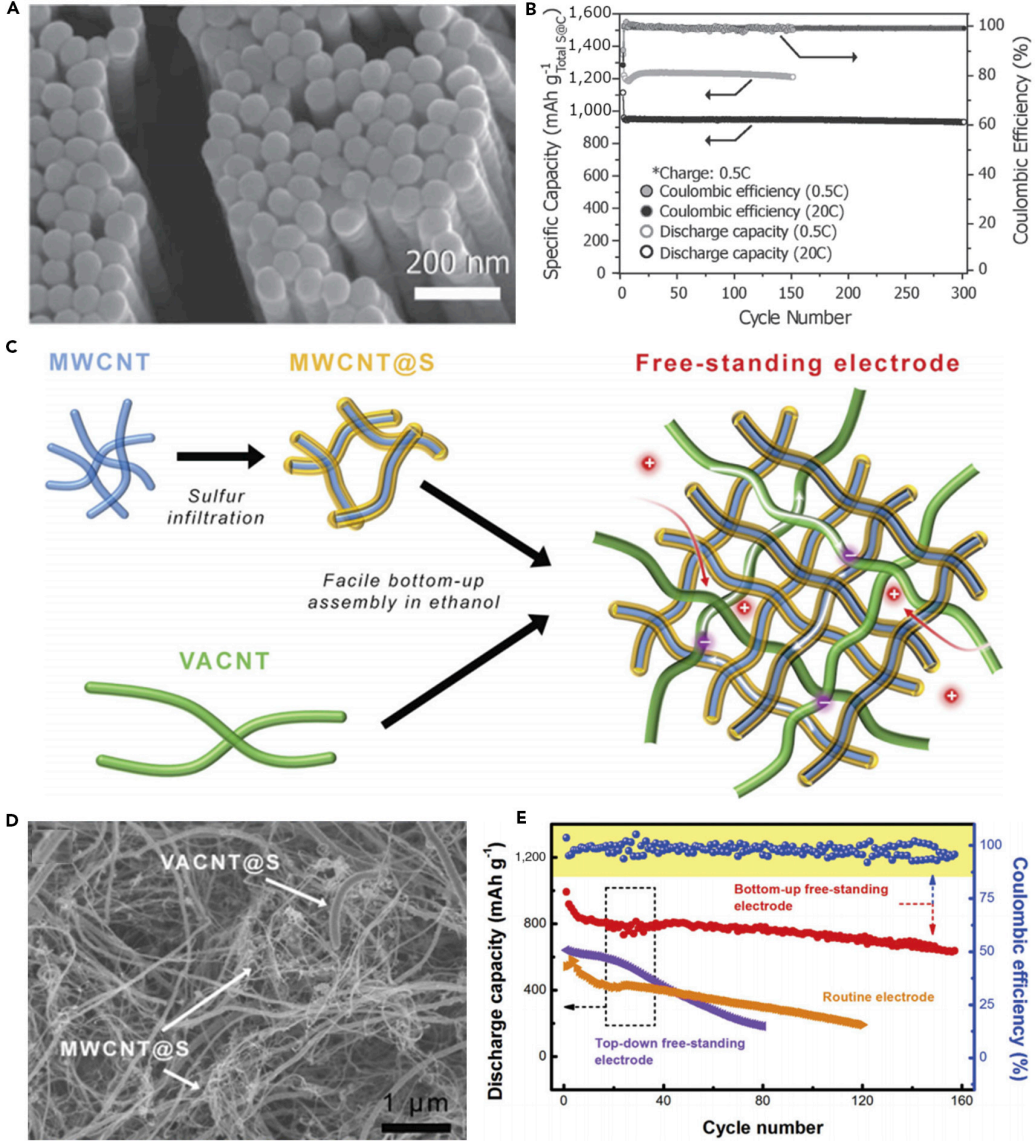
Morphological Characterizations, Synthesis Strategy, and Performance Investigations (A) Scanning electron micrograph of S@C NW array at high magnification. (B) Capacity retentions and Coulombic efficiencies at discharge rates of 0.5 and 20 C. Reprinted with permission from (Moon et al., 2013). Copyright 2013, Wiley-VCH. (C) Schematic illustration of the hierarchical, free-standing electrode with ultrahigh sulfur-loading capability via a facile bottom-up approach. Red and purple spheres represent lithium ions and electrons, respectively. VACNT, vertically aligned carbon nanotube. (D) Scanning electron micrograph of a bottom-up free-standing electrode. (E) Cycling performance of the top-down free-standing electrode and a routinely prepared electrode blade coated on an aluminum foil for comparison at a current density of 0.05 C. Reprinted with permission from (Yuan et al., 2014). Copyright 2014, Wiley-VCH.

To make full use of the advantages of CNTs and address various problems that hinder the practical use of LSBs, a flexible cathode was synthesized through template-directed chemical vapor deposition (CVD), carbon thermoreduction, and ethanol evaporation-induced assembly (Zhou et al., 2012). LSBs assembled with the membrane cathode showed excellent performance. High discharge capacities of 712 mAh g^−1^ (23 wt% S) and 520 mAh g^−1^ (50 wt% S) were attained at a high current density of 6 A g^−1^, and the membrane cathode had a long life of over 100 cycles. CNT matrix possessed high conductivity, elemental sulfur could be confined in CNT walls, and a mesoporous structure contributed to fast ion migration. However, the preparation of S-CNT composite in large quantities at low cost remains a great challenge, and this disadvantageous condition partly offsets the merit of the low-price LSBs. For the same purpose, CNT network has been investigated as a conductive matrix to load sulfur by many researchers (Sun et al., 2016a, Yoo et al., 2016). For example, an encapsulated sulfur electrode was designed by Kim's group (Moon et al., 2013). The well-aligned sulfur nanowires were completely covered by a minimal amount of carbon (Figure 5A). Sulfur@carbon nanotubes nanowires (S@C NW) electrode could address all of the aforementioned issues (such as shuttle effect and volumetric expansion) in LSBs. The carbon layer on the surface of sulfur nanowires could effectively inhibit the dissolution of polysulfides, improve the conductivity of elemental sulfur, and thus result in excellent electrochemical performance. S@C electrode showed a reversible capacity of ∼1,520 mAh g^−1^ at 0.5 C, and more importantly, a high discharge capacity of 960 mAh g^−1^ was retained during 300 deep cycles at a higher C-rate of 20 C (Figure 5B). However, the real reason for causing a stable monoclinic sulfur at room temperature and the accurate effect of such monoclinic phase on the performance of LSBs are unknown, and we need to further explore it.

**Figure 6:**
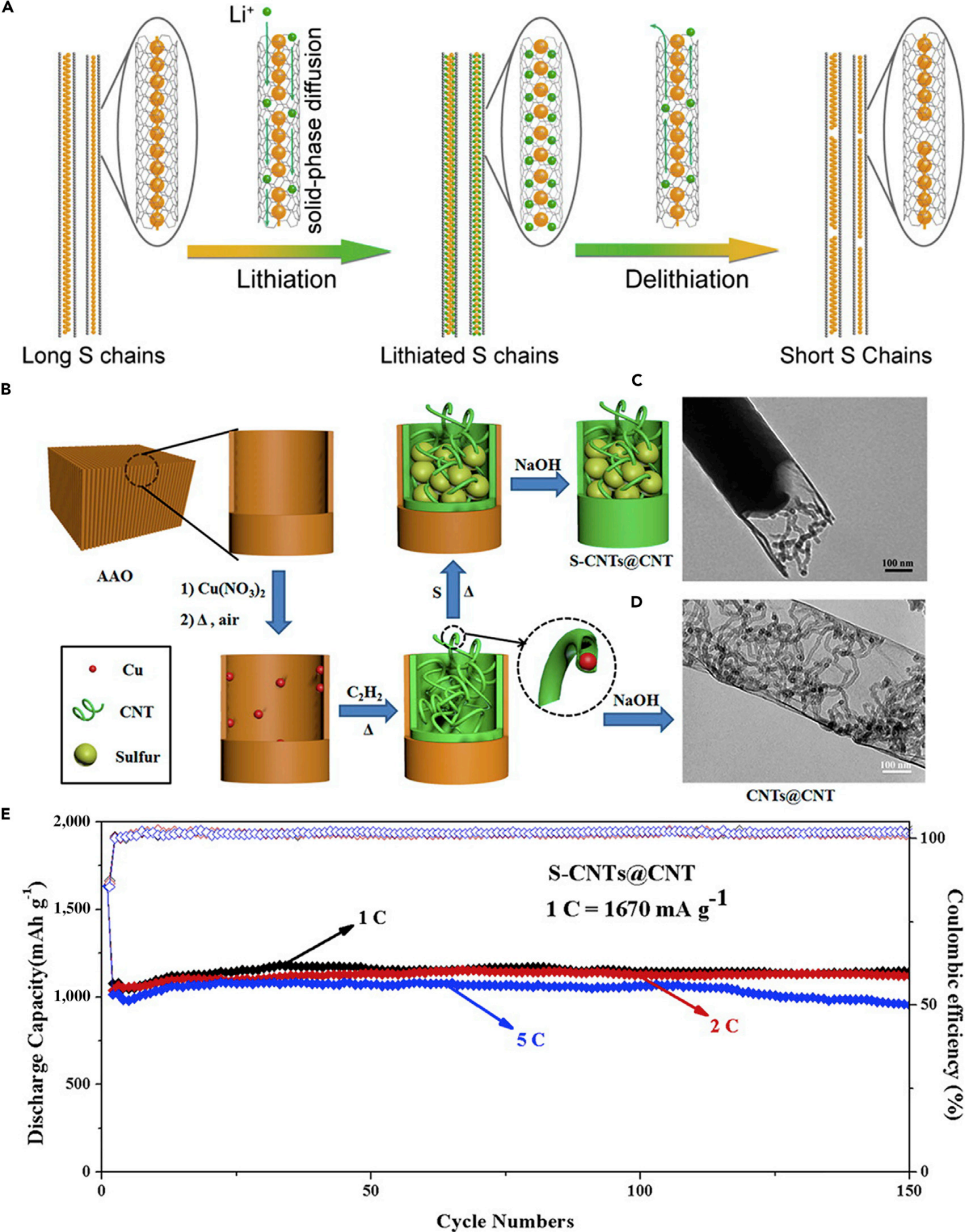
Synthesis Strategy, Lithiation/Delithiation Processes, Morphological Characterization, and Performance Investigations (A) Schematic presentation of proposed electrochemical lithiation/delithiation processes of S chains in a Li-S battery. Reprinted with permission from (Yang et al., 2015). Copyright 2015, American Chemical Society. (B) Schematic illustration showing the templated growth of S@CNT and S-CNTs@CNT. (C) TEM image showing the tip sections: S-CNTs@CNT. (D) TEM image of CNTs@CNT. (E) Cycling performance of S-CNTs@CNT at high current rates of 1, 2, and 5 C. Reprinted with permission from (Jin et al., 2016a). Copyright 2016, American Chemical Society.

Similarly, a hierarchical free-standing CNT-S paper electrode was fabricated by Zhang's group using a facile bottom-up strategy (Figure 5C) (Yuan et al., 2014). In CNT-S paper electrode, an ultrahigh sulfur loading of 6.3 mg cm^−2^ was obtained, the short MWCNTs were used as the short-range electrical conductive network, and super-long CNTs acted as both the long-range conductive network and intercrossed binders (Figure 5D). More importantly, CNT could dramatically enhance the loading content of sulfur in the free-standing CNT-S paper electrode. Hence, the battery assembled with CNT-S paper electrode displays outstanding electrochemical properties. An initial discharge capacity of 6.2 mAh cm^−2^ (995 mAh g^−1^) was obtained, and a low cyclic fading rate of 0.20%/cycle was achieved after 150 cycles at a low current density of 0.05 C (Figure 5E). A higher areal capacity of 15.1 mAh cm^−2^ could be achieved by stacking three CNT-S paper electrodes as a cathode in LSBs as well. To investigate the electrochemistry of S chains in LSBs, Yang et al. (Yang et al., 2015) fabricated a model system by using S chains encapsulated in SWCNTs and double-walled CNTs. Figure 6A describes the electrochemical reaction mechanism of S chains in a Li-S battery. S consisted of S_8_ rings in the structure of S with MWCNTs, which was completely different from 1D S chains encapsulated in CNTs (S/CNTs). The well-defined 1D S chains in CNTs underwent solid-phase electrochemical reaction and had high electrochemical activity during the charge/discharge process, which was proved by the results of electrochemistry measure. S/CNTs cathode showed excellent cycling stability over 20 cycles.

The above-mentioned method can address the critical problems existing in the sulfur cathode of LSBs to some extent, such as poor electrical conductivity, dissolution of lithium polysulfides, and large volumetric change during cycling process, but the loading content of sulfur remains low. Keeping in view these hurdles, Wang's group reported that a tube-in-tube carbon structure was employed to enhance the loading content of sulfur and improve the electrochemical properties of LSBs (Figure 6B) (Jin et al., 2016a). The composite cathode possessed a high loading amount of 85.2 wt% sulfur. The large-diameter amorphous CNTs were filled with sulfur and small-diameter CNTs. Small-diameter CNTs could improve the electrical conductivity of sulfur, mitigate the internal shuttling effect, and enhance structural stability. TEM images of S-CNTs@CNT and CNTs@CNT are shown in Figures 6C and 6D, respectively. L-S battery showed a good cyclability at 0.1 C, and a superior discharge capacity of 1,633 mAh g^−1^ was also achieved at a current rate of 0.1 C, which was near its theoretical capacity. In the meanwhile, large discharge capacities of 1,146, 1,121, and 954 mAh g^−1^ were observed after 150 cycles at higher C-rates of 1, 2, and 5 C, respectively (Figure 6E).

#### Sulfur/Carbon Nanofibers

CNFs hold physicochemical and morphological characteristics, which are similar to those of CNTs. However, they have no hollow space in the middle and a graphitic structure. Due to their good electrical conductivity and excellent mechanical strength, CNFs contribute to intimate electronic contact between sulfur and current collector, and thus improve the utilization of active material during the electrochemical reaction (Ji et al., 2011a). Therefore, CNFs are considered to be promising host matrix candidates for elemental sulfur. Moreover, CNF additive can form the interwoven network structure for suppressing S/Li_2_S agglomeration, which usually covers the sulfur cathode and produces inactivation areas. To inhibit the prominent polysulfide diffusion and achieve high-performance LSBs, Zheng et al. (Zheng et al., 2011) fabricated a hollow CNF-encapsulated sulfur cathode. The hollow CNF arrays were synthesized by utilizing an anodic aluminum oxide template. The high aspect ratio of the hollow CNF arrays provided an ideal structure for suppressing the diffusion of intermediate polysulfides and also offered large space for sulfur expansion during the electrochemical reaction due to the different densities between sulfur and Li_2_S_2_/Li_2_S, and the thin carbon wall allowed rapid transport of lithium ions. The battery assembled with carbon hollow fiber/sulfur composite electrode displayed good cyclability at a C-rate of C/5. For the same purpose, Rao and co-workers designed a novel LSB system (Rao et al., 2012), which combined the advantages of CNFs-sulfur (CNFs-S) cathode with gel polymer electrolyte (GPE). Li-S cell showed excellent electrochemical performance. The improved battery performance could be ascribed to the synergism of CNFs-S cathode and GPE. Specifically, CNFs supplied the effective conduction path, and GPE inhibited the dissolution of the polysulfides. However, the preparation cost of the composite electrode could not be ignored.

**Figure 7:**
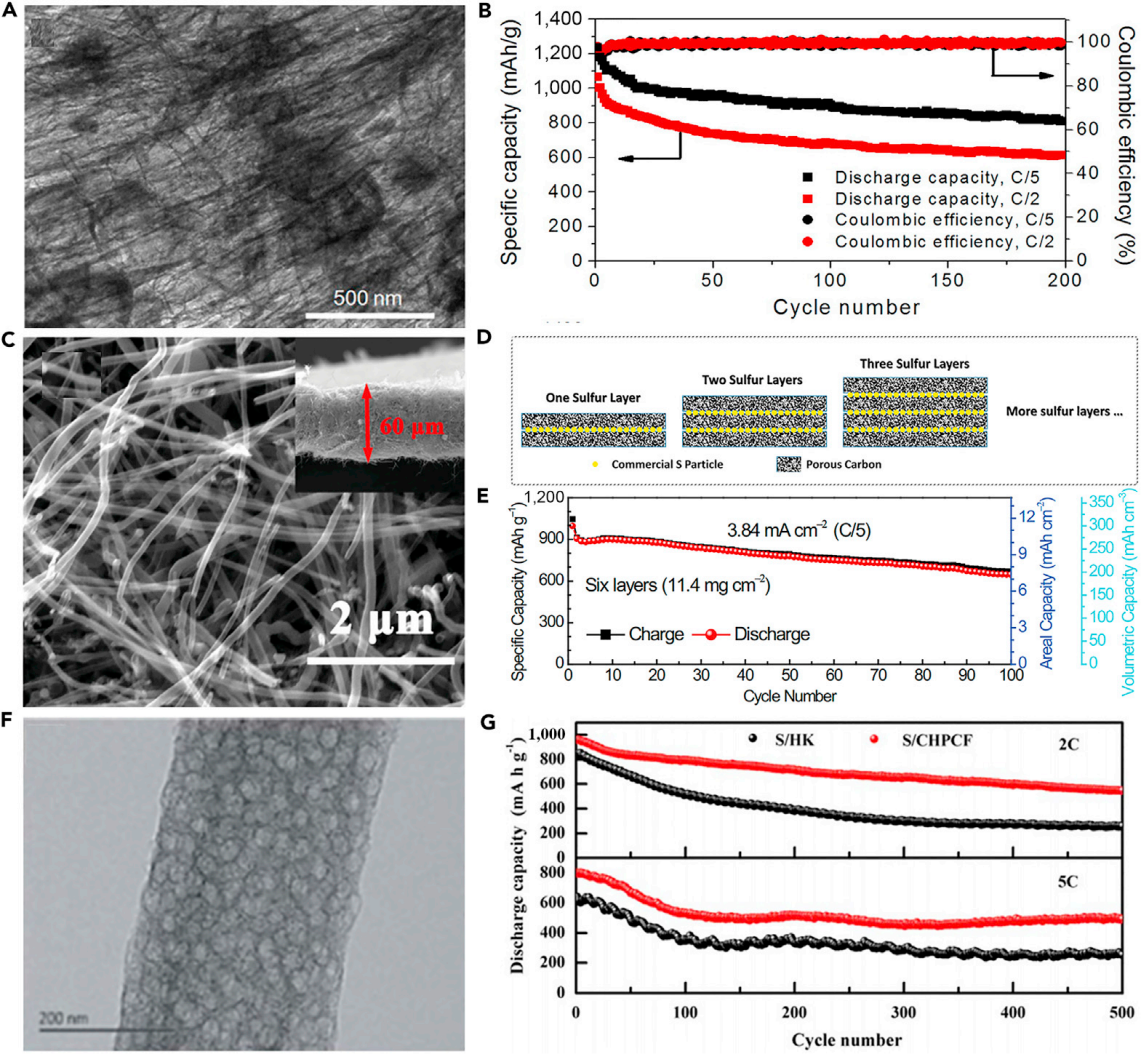
Morphological Characterization and Electrochemical Performance Investigations (A) TEM image of sulfur encapsulated by crab shell-templated carbon showing nanochannel array structures. (B) Capacity and Coulombic efficiency at C/5 and C/2 versus cycle number. Reprinted with permission from (Yao et al., 2013). Copyright 2013, American Chemical Society. (C) Scanning electron micrograph of PCNF layers. (D) Schematic representation of the layer-by-layer sulfur cathodes. (E) Cycling performance of the cell with six-sulfur-layer cathode. Reprinted with permission from (Qie and Manthiram, 2015). Copyright 2015, Wiley-VCH. (F) TEM image of CHPCF. (G) Long-term cycling performance of S/HKC (the carbon derived from HKUST-1) and S/CHPCF electrodes with LiNO_3_ as an additive. Reprinted with permission from (Yang et al., 2016). Copyright 2016, The Royal Society of Chemistry.

With the aim to decrease the cost of preparing nanostructured battery electrode materials, and thus achieve the practical application of LSBs, hollow CNFs were fabricated through using crab shell nanochannel templates by Cui and his team (Yao et al., 2013). The active material sulfur was introduced into hollow CNFs by thermal infusion (Figure 7A). The hollow CNF-S cathode showed a high specific capacity and capacity retention of 60% after 200 cycles at C/5 (Figure 7B). The crab shell nanotemplates possess high surface area and are beneficial to deposit active material. The hollow nanostructures provided sufficient space for accommodating the volumetric variation in the host material during the discharge/charge processes. 1D hollow structures would also reduce the contact of active material with the electrolyte. This biotemplating offered a promising way to synthesize nanostructured electrode materials from low-cost sustainable sources. For the same purpose, Manthiram et al. prepared a high-areal-capacity sulfur cathode via a facile and unique layer-by-layer strategy (Qie and Manthiram, 2015). Figure 7C shows scanning electron microscopy image of porous carbon nanofiber (PCNF) layers. Such unique layer-by-layer cathodes were composed of inexpensive commercial sulfur powder and PCNF papers (Figure 7D), which facilitated ion and electron transport, and effectively trapped the soluble polysulfides within the electrode. Consequently, LSBs assembled with a single-sulfur-layer cathode displayed improved electrochemical performance. Moreover, a six-sulfur-layer cathode possessed a high areal sulfur loading of 11.4 mg cm^−2^, and a high initial discharge capacity of 995 mAh g^−1^ at a C-rate of C/5 was achieved as well (Figure 7E). This approach enhanced the practical application viability of LSBs.

To overcome the issue of lithium polysulfide diffusion and enhance the performance of CNF-based sulfur cathode, Yang et al. (Yang et al., 2016) fabricated a 1D oriented cross-linking hierarchical porous carbon fibers (CHPCFs)-sulfur composite cathode applied in LSBs. CHPCFs possessed cross-linked structure and reasonable hierarchical porous distribution (Figure 7F), which contributed to the transport of ion and electron. Moreover, the large micro-porous surfaces of CHPCFs could confine the notable polysulfide diffusion and accommodate active material. Owing to their unique architecture, S/CHPCF composites demonstrated high specific capacity and long cycle stability over 500 cycles, but still there was significant capacity degradation (Figure 7G). Meanwhile, an outstanding high-rate capability was achieved at 15 C. This strategy can arouse great interest in other advanced high-C-rate energy storage applications.

Carbons as the conductive host matrices for sulfur, such as porous carbons, carbon fibers, CNTs, graphene, or their hybrids, play a significant role in improving the electrical conductivity of sulfur, inhibiting the shuttle effect of dissolvable polysulfides and accommodating the large volumetric change of sulfur composite in the electrochemical process, and thus result in enhanced electrochemical performance. Table 1 simply compares the representative reports to provide an overall perspective of what types of materials are being tested in this area. Elemental sulfur is mainly loaded by melting diffusion, ball-milling, and chemical co-precipitation for different materials. The first method mentioned in Table 1 is melting diffusion. It possesses the advantage of good dispersing effect. However, this method has high energy consumption and the operational process is tedious. The second method is ball-milling, which has many advantages as well as some disadvantages. The advantages can be summarized as follows: (1) grinding in a closed machine has no dust flying; (2) grinding is reliable, and is an intermittent or continuous operation; and (3) grinding has low energy consumption. Despite these advantages, this method has the following drawbacks: (1) strong vibration and noise, (2) bulky size, (3) low efficiency, and (4) staining of products. The chemical co-precipitation technique has also been applied in LSBs by many researchers, and its advantages are as follows: (1) homogeneous mixing of reactant precipitates reduces the reaction temperature and (2) the simple direct process for the synthesis of fine metal oxide powders is highly reactive in low-temperature sintering. Even so, this process is not suitable for the preparation of highly pure, accurate stoichiometric phase. Moreover, it does not work well if the reactants have very different solubility and precipitation rate. As mentioned above, each method has its own characteristics. Therefore, we should choose the appropriate sulfur loading method according to different materials. More recently, advanced carbons with carefully designed microstructures and chemical compositions have been proposed as multifunctional components in LSBs. However, the nonpolar hydrophobic carbonaceous materials have rather weak intermolecular interaction for anchoring the more polar hydrophobic intermediate polysulfides or adhere well to Li_2_S, and hence the diffusion and shuttling of lithium polysulfides still exists during the long-term cycling.

**Table 1:** Summary of Different Carbon Materials Employed in the Cathodes for LSBs

Type of Host Materials	S Content (%)	Initial Discharge Capacity	Final Discharge Capacity (Cycle)	Current Density (mA g^−1^)	Sulfur Infiltration Method	Voltage Window (V)	References
Hierarchical porous carbon	46	1,412	539 (500)	100.5	Melting diffusion	1.7–2.6	(Jung et al., 2014)
Graphene-based layered porous carbon	68	885.5	620 (100)	837.5	Chemical co-precipitation	1.7–2.6	(Yang et al., 2014)
Unstacked double-layer templated graphene	64	1,084	701 (200)	1,675	Melting diffusion	1.7–2.8	(Zhao et al., 2014b)
Amino-functionalized carbon nanotubes	70	950	750 (300)	837.5	Melting diffusion	1.6–2.6	(Ma et al., 2016)
3D multi-walled carbon nanotube frameworks	60	1,600	580 (100)	335	Chemical co-precipitation	1.0–3.0	(Wang et al., 2014a)
Activated multichannel carbon nanofiber	80	1,351	920 (300)	335	Chemical co-precipitation	1.8–2.8	(Lee et al., 2016)
3D graphene nanosheet-carbon nanotube	68.93	1,373.8	836.5 (200)	167.5	Chemical co-precipitation	1.7–2.8	(Zhang et al., 2017c)
Nanosized nickel sulfide-decorated 3D carbon hollow spheres	72	723	695 (300)	837.5	Melting diffusion	1.7–2.8	(Ye et al., 2017)
Hierarchical porous yolk-shell carbon nanosphere	76	679	337 (1,000)	837.5	Melting diffusion	1.7–2.8	(Yang et al., 2017)
Hollow-in-hollow carbon spheres with hollow foam-like cores	70	1,080	780 (300)	1,000	Melting diffusion	1.9–3.0	(Zang et al., 2015)
Nitrogen-doped MOF-derived microporous carbon	27	1,656	936.5 (100)	335	Melting diffusion	1.0–3.0	(Li and Yin, 2015)
Hollow core-shell interlinked carbon spheres	70	1,100	960 (200)	837.5	Melting diffusion	1.7–2.8	(Sun et al., 2015)
3D hyperbranched hollow carbon nanorod	71.5	1,255	1,147 (500)	836.5	Chemical co-precipitation	1.7–2.6	(Chen et al., 2014a)
Three-dimensional porous carbon	90	1,115	670 (1,000)	3,350	Chemical co-precipitation	1.5–3.0	(Li et al., 2016a)
Hollow carbon nanofiber	60.8	1,090	600 (100)	1,675	Chemical co-precipitation	1.5–3.0	(Li et al., 2014b)
Pyrrole-modified graphene foam	42	985.8	797.9 (100)	837.5	Melting diffusion	1.5–3.0	(Zhang et al., 2017a)
Cation-functionalized pigment nanocarbon	85	1,223	1,010 (100)	167.5	Chemical co-precipitation	1.7–2.8	(Zeng et al., 2017)
Microporous carbon polyhedron-encapsulated polyacrylonitrile nanofibers	52	1,518.6	1,282 (100)	160	Melting diffusion	1.0–3.0	(Zhang et al., 2017b)
Honeycomb-like ordered mesoporous carbon	69.8	1,238	505.7 (500)	837.5	Melting diffusion	1.7–2.8	(Park et al., 2017)
Popcorn-inspired porous macrocellular carbon	76.1	1,257.2	821 (500)	335	Melting diffusion	1.7–2.8	(Zhong et al., 2017)
Sandwich-type hybrid carbon nanosheets	74	1,200	860 (100)	1,675	Melting diffusion	1.7–2.8	(Chen et al., 2014b)
3D interconnected porous carbon aerogels	27	2,368	822 (50)	100	Melting diffusion	1.0–3.0	(Zhang et al., 2014b)

The capacity in the brackets is calculated according to the mass of the active material sulfur, and the unit is mAh g^−1^.

### Sulfur/Metal Oxide Binary Composite Materials

It is acknowledged that major challenges in the practical implementation of LSBs reside in the dramatic capacity decay. The diffusion of intermediate polysulfides during cycling leads to shuttle effect and thus results in the pronounced capacity fading. Carbon materials are typically used as the host matrices for sulfur, but can only partially restrict polysulfide intermediates, and the hydrophilic lithium polysulfide molecules will diffuse out from carbon host over long-term cycling. That is to say, pure carbons cannot serve as ideal matrices. Metal oxides are the most promising inorganic compounds to anchor polysulfides in LSBs. Metal oxides have many advantages, such as strong adsorption performance and high specific surface area. They can increase the contact area between the electrode and electrolyte and inhibit the dissolution of the discharge products into the electrolyte. Metal oxide that typically contains an anion of oxygen in the oxidation state of O^2−^ can provide a strong polar surface, efficiently trap lithium polysulfides, and improve the utilization of elemental sulfur. Owing to the strong binding between the oxygen and the metal, metal oxides tend to be insoluble in most organic solvents. Moreover, the volumetric energy density of LSBs is enhanced by fabricating metal oxide and sulfur composite cathode, and the conversion reaction between polysulfide intermediates and Li_2_S_2_/Li_2_S can be promoted dramatically. Because of the advantages of polar metal oxides, they have been extensively explored as excellent additives in the cathodes of LSBs (Tao et al., 2016). Very recently, many researchers have turned their attention toward metal oxides. For example, to inhibit polysulfide dissolution into liquid electrolyte and promote Li/S redox reaction, Lee and co-workers prepared nano-sized Mg_0.6_Ni_0.4_O used as an additive in LSBs (Song et al., 2004). Owing to the nano-sized Mg_0.6_Ni_0.4_O's catalytic effect and the polysulfides' adsorbing effect the porosity of the sulfur cathode could also be increased. Mg_0.6_Ni_0.4_O and MgO had the same crystal structure and the effect of retaining liquid electrolyte. However, Mg_0.6_Ni_0.4_O possessed a catalytic effect of dissociating the chemical bond (e.g., N=O; bond dissociation energy 607 kJ/mol). Compared with N=O, the dissociation energy of S-S single bond was lower (255 kJ/mol). In view of this, this feature could be applied to LSBs. Hence, the discharge capacity and cycle durability of LSBs were enhanced: the initial charge-discharge capacity was increased from 741 to 1,185 mAh g^−1^ and the cyclic durability was also improved from 76% of the initial discharge capacity at the 50th cycle to 85% after the addition of nano-sized Mg_0.6_Ni_0.4_O additive. Although the performance of LSBs was improved at the 50th cycle, the discharge capacity continuously decreased after the 50th cycle. It was demonstrated that polysulfide dissolution into electrolyte still existed. Similarly, another metal oxide (nano-sized Al_2_O_3_) was added into sulfur electrode to improve the electrochemical properties of cathode for LSBs (Xu et al., 2017). After Al_2_O_3_ nanoparticles were added into sulfur electrode, the properties of LSBs were also enhanced. An increase in specific capacity (402 mAh g^−1^ without additive versus 741 mAh g^−1^ with additive) was obtained at 0.06 C. The improvement of discharge capacity and cycle performance was attributed to the polysulfides' adsorption effect of porous nano-sized Al_2_O_3_ particles. Nano-sized Al_2_O_3_ being a metal oxides, its small particle size and large surface area could prominently improve the efficiency of adsorbing polysulfides. Although the discharge capacity was relatively high and cycle performance was good until the 25th cycle, the cycle life needed to be prolonged.

**Figure 8:**
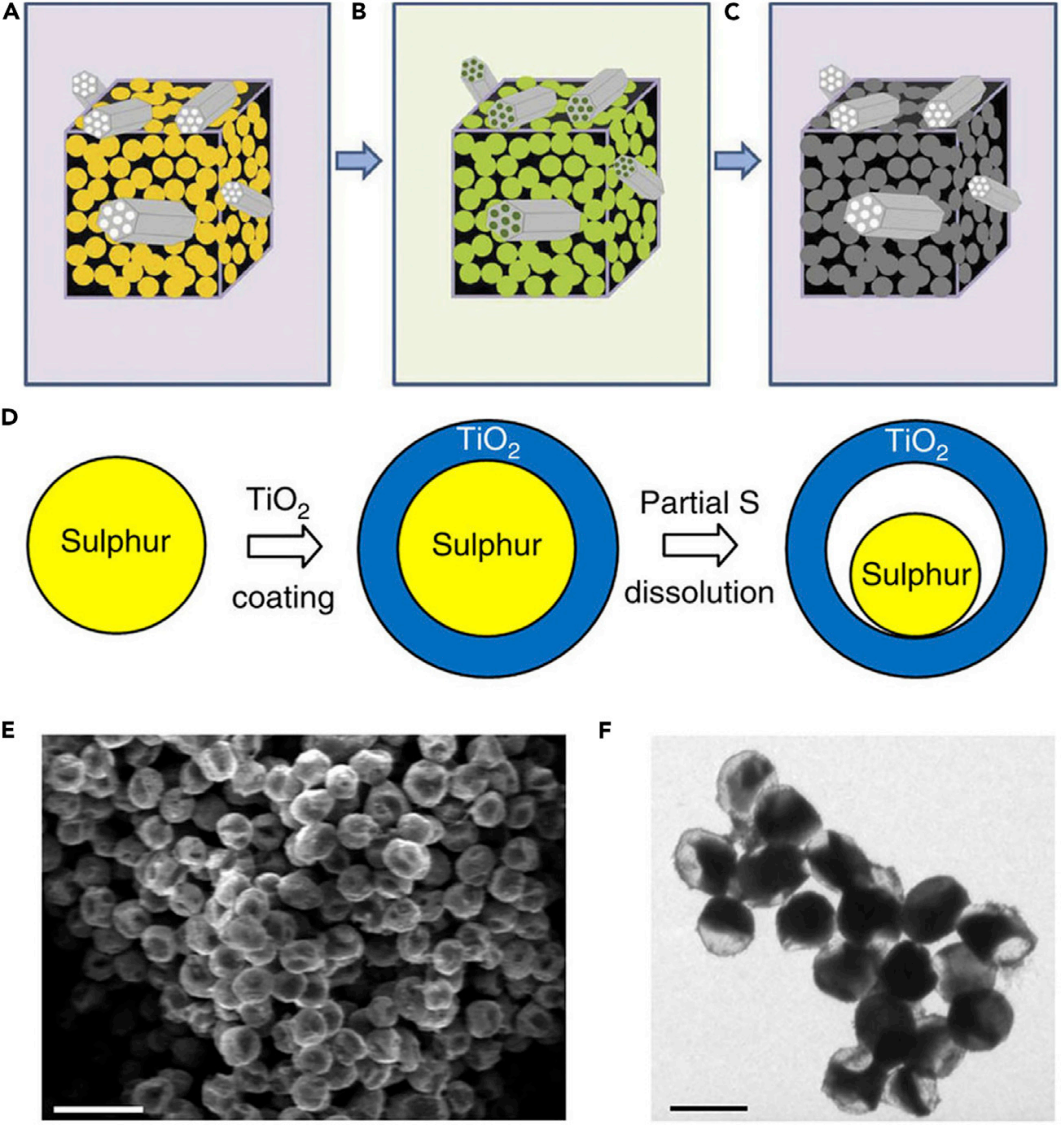
Discharge Process, Synthesis Strategies, and Morphological Characterization (A) Before discharge: the black area represents carbon with pores infiltrated by sulfur and gray particles on the cube surface are SBA-15. (B) Discharge to 2.15 V: the green-colored area denotes polysulfide ions (S_n_^2−^, 3 ≤ n ≤ 8), which are “concentrated” in the SBA-15. (C) Discharge to 1.5 V: polysulfide ions diffuse out of SBA-15 platelets and are further reduced into solid sulfides (Li_2_S/Li_2_S_2_) within the silica colloidal monolith (SCM) carbon framework. Reprinted with permission from (Ji et al., 2011c). Copyright 2011, Nature Publishing Group. (D) Schematic of the synthetic process that involves coating of sulfur nanoparticles with TiO_2_ to form sulfur-TiO_2_ core-shell nanostructures, followed by partial dissolution of sulfur in toluene to achieve the yolk-shell morphology. (E and F) (E) Scanning electron micrograph and (F) TEM images of as-synthesized sulfur-TiO_2_ yolk-shell nanostructures. (E) Scale bar, 2 μm. (F) Scale bar, 1 μm. Through large-ensemble measurements, the average nanoparticle size and TiO_2_ shell thickness were determined to be 800 and 15 nm, respectively. Reprinted with permission from (Seh et al., 2013). Copyright 2013, Nature Publishing Group.

Like nano-sized Mg_0.6_Ni_0.4_O and Al_2_O_3_, porous SiO_2_ is also employed in LSBs. To mitigate the problem of polysulfide dissolution, Nazar and his team reported a new concept on the basis of the design principles of drug delivery (Ji et al., 2011c). Porous silica was embedded into the carbon-sulfur composite to absorb the intermediate polysulfides and permit reversible desorption/release (Figures 8A–8C). That is to say, porous silica served as a highly effective internal polysulfide reservoir for the cathode in LSBs. Compared with the pure sulfur cathode, the battery assembled with SBA-15 delivered a high initial discharge capacity. SiO_2_ possessed high surface area, large pore volume, biconnected porous structure, and highly hydrophilic surface, and displayed strong adsorption capacity during the electrochemical process. SiO_2_ could adsorb polysulfides mainly due to its mesoporous structure. Therefore, it was used as a highly effective internal polysulfide reservoir for the cathode in LSBs. Therefore, porous SiO_2_ played an important role in improving the electrochemical performance of LSBs. Nano-sized metal oxides mentioned above were used as additives in LSBs. However, there was relatively little emphasis on dealing with the volumetric change of sulfur during the charge/discharge process. To mitigate this problem, Cui and his team designed a sulfur-TiO_2_ yolk-shell nanoarchitecture for the first time (Figure 8D) (Seh et al., 2013). The yolk-shell nanoarchitecture could offer an internal void space to accommodate the volumetric variation of sulfur and confine lithium polysulfides within the shell to enhance the utilization of active material during the electrochemical reaction (Figures 8E and 8F). More importantly, the highly exposed (001) facets and Sn^2−^ could form strong physicochemical interaction, and thus restrained the dissolution of polysulfides and the notorious shuttling effect. S_4_^2−^ was likely to be trapped at oxygen-defective sites and coordinated with two Ti^3+^ sites. This was the main reason that polysulfides could be adsorbed by TiO_2_. Consequently, compared with sulfur-TiO_2_ core-shell and pure sulfur nanoarchitecture, the sulfur-TiO_2_ yolk-shell nanoarchitecture exhibited the best stable cycling performance, and the rate of capacity decay was only 0.033% per cycle after 1,000 cycles. TiO_2_ was also used as an adsorbent in the sulfur cathode and could improve the cycle performance of LSBs to a certain extent.

**Figure 9:**
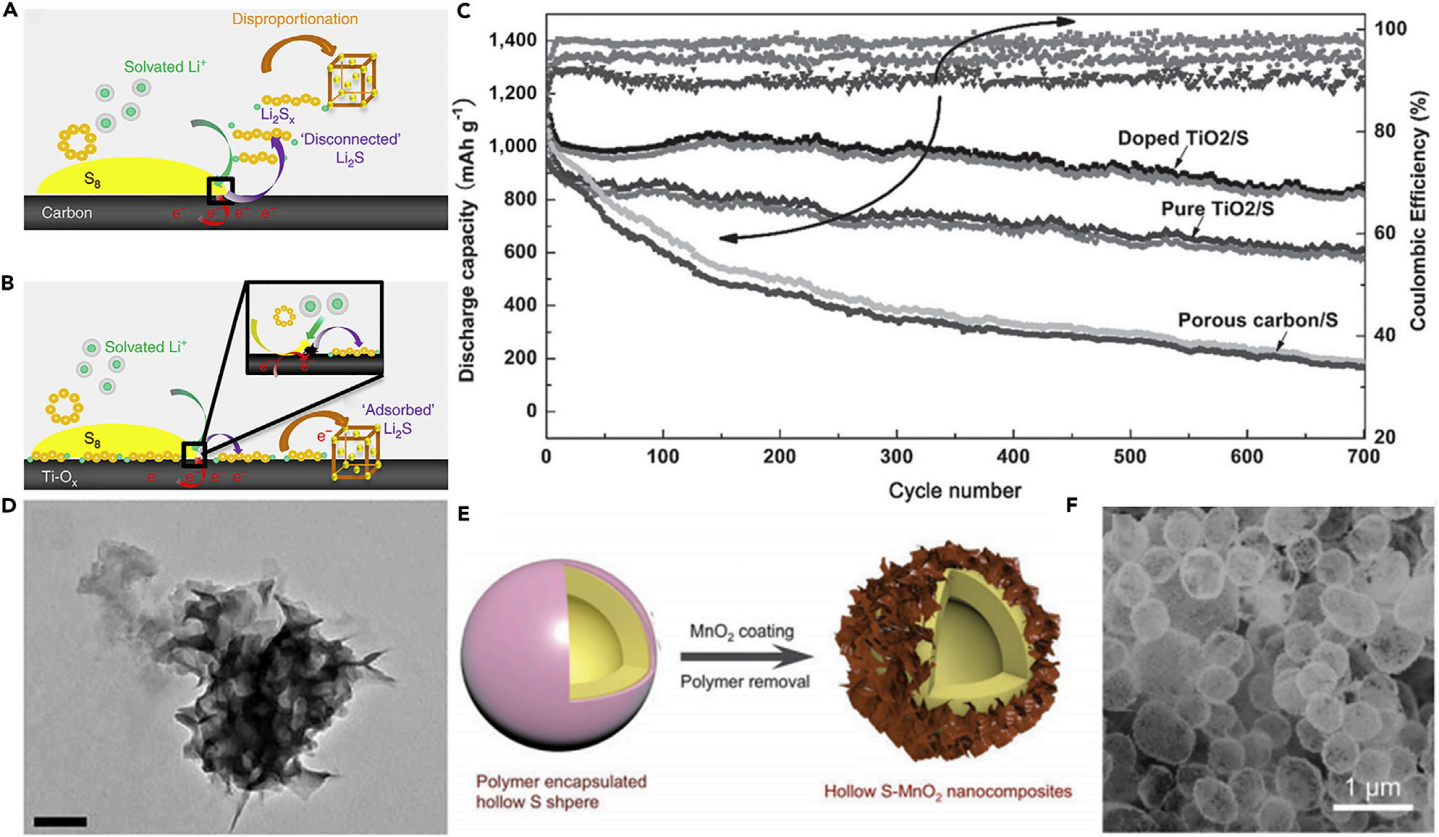
Surface-Mediated Reduction, Performance Investigations, Morphological Characterization, and Synthesis Strategies (A) On reduction of S_8_ on a carbon host, LiPSs (Li_2_S_X_) desorb from the surface and undergo solution-mediated reactions leading to broadly distributed precipitation of Li_2_S. (B) On reduction of S_8_ on the metallic polar Ti_4_O_7_, LiPSs adsorb on the surface and are reduced to Li_2_S via surface-mediated reduction at the interface. Reprinted with permission from (Pang et al., 2014). Copyright 2014, Nature Publishing Group. (C) Cycling performance and Coulombic efficiency of doped TiO_2_/S, pure TiO_2_/S, and porous carbon/S cathode at 0.5 C. Reprinted with permission from (Wang et al., 2016b). Copyright 2014, Wiley-VCH. (D) TEM image of S/MnO_2_ nanosheets composite. Reprinted with permission from (Liang et al., 2015b). Copyright 2015, Nature Publishing Group. Scale bar: 200 nm. (E) Schematic of the synthetic process of the hollow S-MnO_2_ nanocomposites. (F) Scanning electron micrograph of the hollow S-MnO_2_ nanocomposite spheres. Reprinted with permission from (Wang et al., 2016c). Copyright 2016, The Royal Society of Chemistry.

Following the above research, conductive Magnéli phase Ti_4_O_7_ was further investigated, and it was a highly effective matrix to bind with sulfur active species. This was demonstrated in experiment and theoretical calculation. Pang et al. (Pang et al., 2014) reported a new class of inorganic sulfur host material, which possessed a good electronic conductivity and a polar surface. It could suppress the dissolution of polysulfides by forming an excellent interface with Li_2_S (Figures 9A and 9B), and thus improve the electrochemical performance of LSBs. In the meantime, Ti_4_O_7_ showed excellent adsorbing capability, and Ti_4_O_7_/S-60 battery delivered a discharge capacity of 1,070 mAh g^−1^ at intermediate rate. When the percent of sulfur was compared with those of mesoporous carbons, the electrodes of LSBs delivered better cycling stability. This result demonstrated that surface interaction played a large role in mitigating sulfide dissolution and deposition than mesoporous confinement. More importantly, the surface interaction contributed to converting polysulfides into Li_2_S, and enhancing redox electron transfer. This result was consistent with theoretical calculation of Cui's group (Tao et al., 2014). Similarly, Wei and co-workers prepared mesoporous Magnéli Ti_4_O_7_ microsphere by *in situ* carbothermal reduction, which possessed interconnected mesopore (20.4 nm), large pore volume (0.39 cm^3^ g^−1^), and high surface area (197.2 m^2^ g^−1^) (Wei et al., 2016). Owing to the strong chemical bonding between lithium polysulfides and Ti_4_O_7_, and effective physical trapping in the mesopores and voids in the matrix, a sulfur hosted on Magnéli Ti_4_O_7_ showed excellent electrochemical performance.

Titanium dioxide (TiO_2_) as a metal oxide host has been investigated by many researchers (Ma et al., 2015b). Inspired by Cui's study on TiO_2_, Wang's group reported that surface acidity of the host material played an important role in the chemisorption of polysulfides (Wang et al., 2016b). The stronger the surface acidity of metal oxide host, the higher the capability of polysulfide chemisorption. The surface acidity of TiO_2_ was tailored by heteroatom doping, and the polysulfide-TiO_2_ interaction could be fortified, hence the electrochemical performance of LSBs was improved. Meanwhile, a low capacity fading of 0.04% was obtained after 700 cycles at 0.5 C (Figure 9C). The improved properties of LSBs could be attributed to the strengthened polysulfide chemisorption. The surface acidity of TiO_2_ host formed a stronger Ti-S bond with polysulfide anion than porous carbon. Consequently, the doped TiO_2_/S composite cathode exhibited better cycling performance.

Other metal oxides have also been used to improve the cathode properties. For instance, MnO_2_ (Liang and Nazar, 2016), MgO (Ponraj et al., 2016), Co_3_O_4_ (Wang et al., 2016a), ZnO (Liang et al., 2015c), and SnO_2_ (Cao et al., 2016a) can be used together with sulfur to form metal oxide-sulfur composite electrode and thus improve the electrochemical performance of LSBs. Liang et al. (Liang et al., 2015b) reported a strategy to entrap polysulfides in the cathode, which was based on a chemical process. This process could be described as a host reaction with initially formed lithium polysulfides to form surface-bound intermediates. During this chemical process, thiosulfate groups were created *in situ* on the surface of ultra-thin MnO_2_ nanosheets for the first time. Figure 9D shows the TEM image of S/MnO_2_ nanosheets. The surface thiosulfate groups could anchor the newly formed soluble “higher” polysulfides and convert them to insoluble “lower” polysulfides. This process played an important role in decreasing the active mass loss during cycling process and inhibiting the shuttle effect of polysulfides. Thus, a low capacity decay of 0.036% over 2,000 cycles at 2 C and high capacity retention of 92% after 200 cycles at a current rate of 0.2 C were achieved. Inspired by Liang's work on using non-conductive manganese dioxide as a host to entrap polysulfides in the cathode, an innovative strategy to efficiently entrap Li_x_S_n_ via the synergistic effect of structural restriction and chemical encapsulation using metal oxide-decorated hollow sulfur spheres was proposed by Chen's group (Wang et al., 2016c). Manganese dioxide nanosheet-decorated hollow sulfur sphere nanocomposites were fabricated by a facile synthesis method (as shown in Figure 9E), and its scanning electron micrograph is shown in Figure 9F. The hollow sphere is beneficial to alleviate the volumetric expansion of sulfur composite and encapsulate polysulfides within the spherical structure. The decorated MnO_2_ nanosheets effectively restricted lithium polysulfide dissolution. Consequently, this material architecture for LSBs provided a prolonged cycling stability. LSBs assembled with MnO_2_ nanosheet-decorated hollow S sphere (hollow S-MnO_2_) nanocomposites delivered excellent electrochemical performance. A discharge capacity of 644 mAh g^−1^ and an extremely low capacity decay rate of 0.028% could still be achieved after 1,500 cycles at a C-rate of 0.5 C. Compared with Liang's work and other reports over 1,000 cycles, the capacity decay was very low.

### Sulfur/Conductive Polymer Binary Composite Materials

Conducting polymers have been explored as host matrices to physically adsorb and hold sulfur and play a significant role in improving the electrochemical performance of LSBs. Compared with traditional carbon-based materials, conductive polymers have the following advantages: (1) conductive polymers are usually synthesized using chemical oxidation method at lower temperatures, and the synthetic method is also relatively easy; (2) the good mechanical resilience can partially buffer volumetric variation in the host material during charge/discharge process and alleviate the pulverization of cathode material; (3) the unique chain structures and rich variety of functional groups can effectively confine elemental sulfur and its redox products in composites with controlled morphology by inter- and/or intra-chain bonding, which can inhibit the dissolution of polysulfides into electrolyte, and improve the utilization rate of the active material; and (4) their inherently conducting nature can solve the problem of insulating nature of sulfur. Furthermore, as some polymers are electrochemically active, they can be regarded as a part of the active material to provide additional capacity for the sulfur-polymer composite electrode. Therefore, they are good candidates in the fabrication of polymer-sulfur hybrid materials. In this section, we mainly investigate sulfur/conductive polymer binary composite materials as the cathodes of LSBs.

Polypyrrole (PPy) is the earliest conductive polymer studied as a coating material for cathode in LSBs (Liang et al., 2015d, Zhang et al., 2016b, Ma et al., 2014a, Xin et al., 2017). In 2006, sulfur-PPy composites were synthesized by Wang and co-workers for the first time in this field (Wang et al., 2006). The composites were prepared by using oxidative polymerization of pyrrole onto commercial sulfur particles with iron (III) chloride as an oxidizing agent. After conductive PPy was coated on the surface of sulfur, the electrical conductivity, the capacity, and the cycle durability of S-PPy electrode were improved. Compared with the pristine case, the composite showed a high initial capacity. PPy nanoparticles coated on the surface of sulfur improved the conductivity of the cathode, slowed down the dissolution of the polysulfides to some extent, and contributed to capacity of the electrode during the electrochemical reaction. The improved conductivity and kinetics could be verified by electrochemical impedance spectroscopy measurements on pure S and S-PPy electrodes. However, the capacity decay was observed over 20 cycles, indicating that there was still intermediate polysulfide dissolution into the electrolyte. Following pioneering work, PPy with various morphologies was synthesized, such as tubules (Li et al., 2017a), hollow sphere (Ma et al., 2014b), and nanowire (Sun et al., 2008). Inspired by Wang's work on preparing sulfur-PPy composite materials by the chemical polymerization method, Sun et al. (Sun et al., 2008) prepared S-PPy composite materials by heating the mixture of elemental sulfur and PPy nanowire. The composite cathodes showed excellent electrochemical properties in LSBs. The special morphology of PPy greatly contributed to the excellent electrochemical performance of S-PPy cathode.

**Figure 10:**
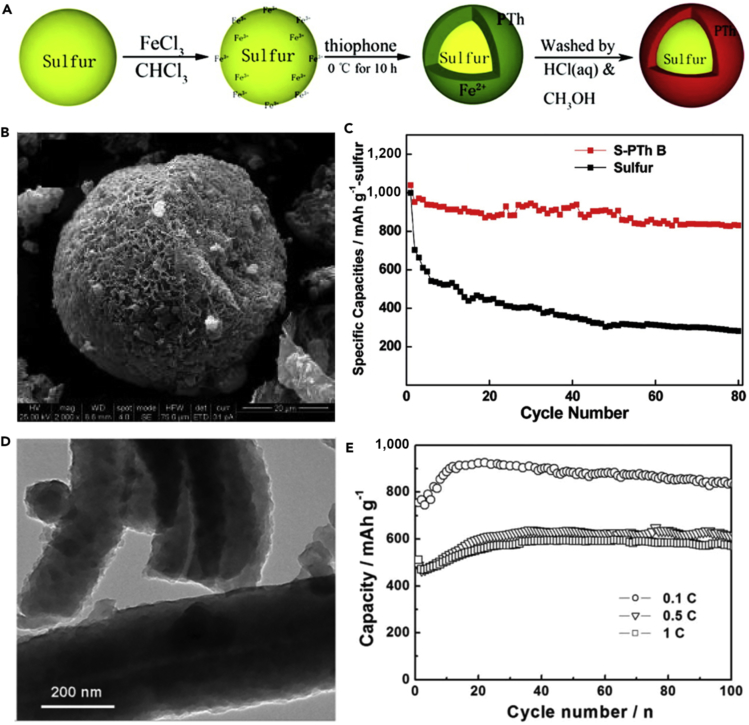
Synthesis Strategies, Morphological Characterization, and Performance Investigations (A) Synthesis of S-polythiophene (PTh) composite. (B) Scanning electron micrograph for core/shell S-PTh B (the ratio of elemental sulfur is 71.9%). (C) Discharge capacities versus cycle number for sulfur and S-PTh B composite cathodes at a current density of 100 mA g^−1^. Reprinted with permission from (Wu et al., 2011). Copyright 2011, American Chemical Society. (D) TEM image of SPANI-NT/S composite. (E) Discharge capacities versus cycle numbers of the electrode at different rates as labeled. Reprinted with permission from (Xiao et al., 2012). Copyright 2012, Wiley-VCH.

To further confine polysulfide dissolution and thus improve the electrochemical properties of LSBs, the conductive polythiophene was applied in LSBs. Wu et al. (Wu et al., 2011) prepared a novel sulfur/polythiophene composite with core/shell structure by an *in situ* chemical oxidative polymerization method (Figure 10A). This approach used chloroform as a solvent, thiophene as a reagent, and iron chloride as an oxidant. The core/shell structure was verified by using TEM image (Figure 10B). A suitable ratio for the composite was found to be 71.9% sulfur and 18.1% polythiophene because the composite at this ratio showed the best electrochemical properties in the rechargeable lithium battery. Conductive polythiophene acted as both a conducting additive and a porous adsorbing agent. The pore and thickness of the shell on the surface of sulfur were of benefit to lithium-ion diffusion from the surface to the core sulfur. Consequently, conductive polythiophene was coated onto the surface of the sulfur powder, and a core/shell structure was formed, which led to a remarkable improvement in the electrochemical performance of LSBs. The composite showed a high initial capacity of 1,119.3 mAh g^−1^ at a current rate of 0.06 C (Figure 10C). The capacity retention of 74% was obtained after 80 cycles when used as cathode material for LSBs. The long cycling life, cycling stability, and rate capability are critical to practical applications of LSBs. To promote practical application of LSBs, polyaniline (PANI) has been applied in lithium secondary batteries. For example, Liu et al. (Xiao et al., 2012) synthesized the self-assembled polyaniline nanotubes (PANI-NT) to encapsulate sulfur, which resulted in a notable improvement in the electrochemical properties of LSBs. The synthesis process of PANI-NT was facile and environmentally benign. The sulfur-PANI nanotube (SPANI-NT) composites were prepared by an *in situ* vulcanization process in which a mixture of PANI-NT and sulfur was heated at 280°C. Figure 10D shows its TEM image. A part of sulfur could react with the polymer to form a 3D structurally stable polymer backbone during the vulcanization process, which was beneficial to encapsulate the sulfur and lithium polysulfides. The soft polymer matrix and nanostructures could effectively accommodate the volumetric expansion and improve the reversible electrochemical reaction and conversion of sulfur species during the charge/discharge process. Therefore, a high discharge capacity of 837 mAh g^−1^ was maintained after 100 cycles at a C-rate of 0.1 C (Figure 10E).

**Figure 11:**
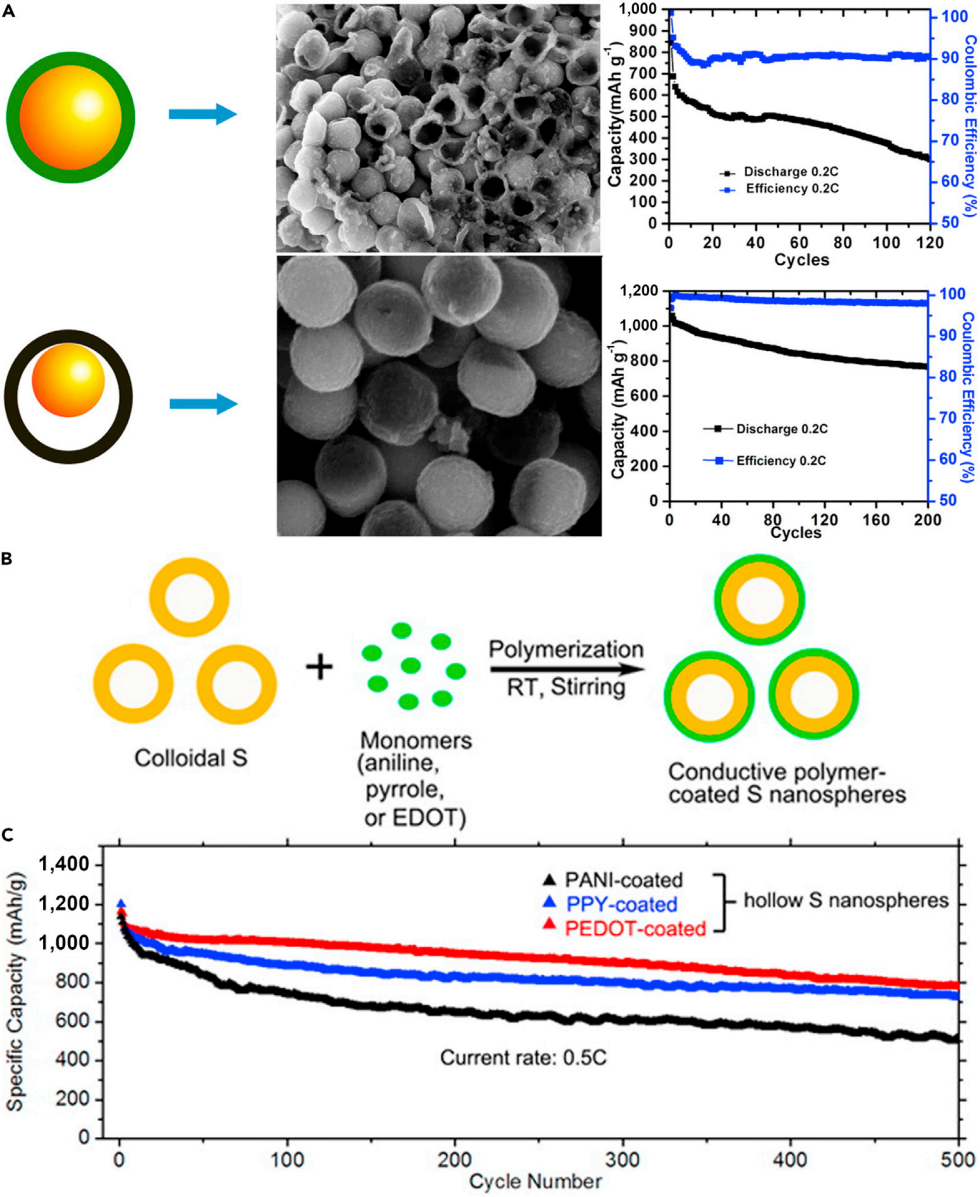
Morphological Characterization, Performance Investigations, and Synthesis Strategies (A) Schematic comparison of S-PANI core-shell and yolk-shell, scanning electron micrograph after running five cycles in cell, and the long-term cycling performance. Reprinted with permission from (Zhou et al., 2013b). Copyright 2013, American Chemical Society. (B) Schematic illustration of the fabrication process of conductive polymer-coated hollow sulfur nanospheres. (C) Cycling performance of the cells made from hollow sulfur nanospheres with PANI, PPy, and PEDOT coatings at C/2 rate for 500 cycles. Reprinted with permission from (Li et al., 2013). Copyright 2013, American Chemical Society.

Following this research, another morphology of PANI was employed in LSBs; for example, a PANI-sulfur yolk-shell nanocomposite was prepared through heating vulcanization of a PANI-sulfur core-shell structure (Zhou et al., 2013b). With the aim of exhibiting that yolk-shell structure possessed excellent electrochemical properties, sulfur-polyaniline (S-PANI) core-shell nanoarchitecture was fabricated by a uniform polymer coating on the surface of the sulfur particles. The yolk-shell composite and polymer shell could offer internal void space to buffer the volumetric expansion from sulfur particles during the charge/discharge process, maintain the intact shells to encapsulate the polysulfides and the elemental sulfur within the polymer shell, and maximize the capacity retention. By contrast, most PANI shells of the core-shell composite were cracked after running 5 cycles in coin cells, as shown in Figure 11A. S-PANI yolk-shell composite electrode obviously displayed improved cycling stability with a high initial capacity of 1,101 mAh g^−1^. Stable capacities of 765 and 628 mAh g^−1^ were obtained at current rates of 0.2 and 0.5 C after 200 cycles, respectively. However, slight capacity fading was also observed in the long term and with repeating discharging/charging processes, that is to say, polysulfide dissolution still existed.

The conductive polymer-sulfur composites employed in previous studies possess greatly different structural configurations. However, the role of different conductive polymers in the electrochemical performance of LSBs remains poorly understood. To solve this problem, Cui's group investigated the effects of different conductive polymers on the electrochemical properties of sulfur electrode systematically (Li et al., 2013). Conductive polymer-coated hollow sulfur nanospheres were prepared via a facile, versatile, and scalable polymerization process, in which monodisperse hollow sulfur nanospheres were coated by PANI, PPy, and poly(3,4-ethylenedioxythiophene) (PEDOT) (Figure 11B), respectively. Cui et al. investigated some effects on sulfur cathode performance through experimental observation and theoretical simulation. They found that PEDOT-S showed excellent cycling stability in long-term charge/discharge process, and a high discharge capacity of 780 mAh g^−1^ was achieved at a higher current rate of C/2 after 500 cycles (Figure 11C). The results of theoretical simulation demonstrated that PEDOT possessed a much stronger binding energy with lithium atom in Li-S than PANI and PPy. Consequently, it could effectively inhibit polysulfide dissolution. Under identical experimental conditions, among all the three polymers, PEDOT possessed the highest conductivity, indicating the best electrode kinetics and stability. The capability of these three polymers in improving long-term cycling stability and high-rate performance of the sulfur cathode was found to follow the order: PEDOT > PPy > PANI. This order was consistent with the results of theoretical calculations.

### Sulfur/Metal Sulfide Composites

Similar to metal oxides, metal sulfides have also been applied as sulfur hosts for LSBs. Metal sulfides consist of 3D networks of the metal and discrete S_2_^2−^ units. Compared with their metal oxide counterparts, metal sulfides possess higher electrochemical activity and redox chemistry due to their more covalent nature. Moreover, metal sulfides display stronger polysulfide affinity and lower overpotential than carbon-based materials. However, the electronic conductivities of metal sulfides are relatively poor, and intermediate polysulfides only can be chemically absorbed near the surfaces. Therefore, not all the polysulfides can be effectively entrapped in high-sulfur-loading cathode.

**Figure 12:**
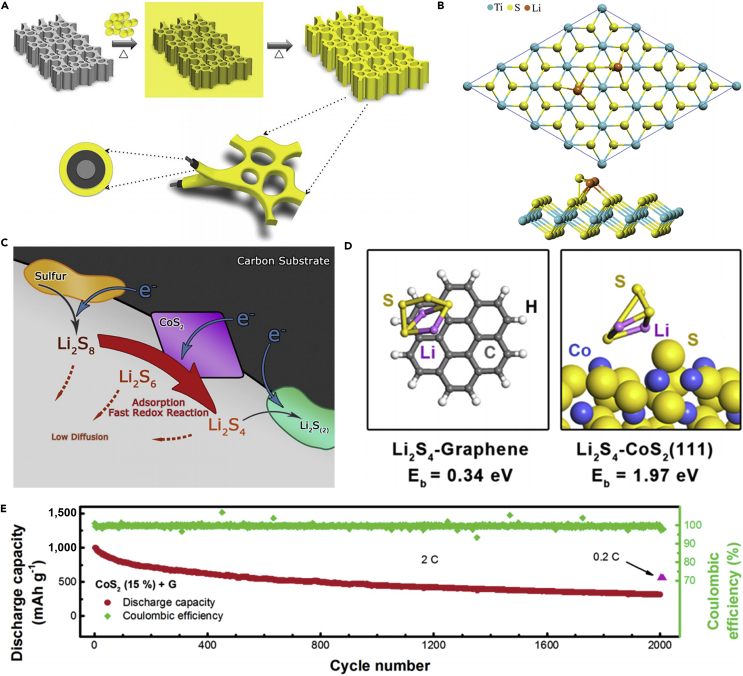
Synthesis Strategies, First-Principle Calculations, Discharge Process, and Electrochemical Performance Investigations (A) One-step method to prepare sulfur-infused TiS_2_ foam. Yellow: sulfur; dark gray: TiS_2_; gray: Ti. (B) Top and side views of Li_2_S adsorption on TiS_2_. Reprinted with permission from (Ma et al., 2015a). Copyright 2015, The Royal Society of Chemistry. (C) CoS_2_-incorporated carbon/sulfur cathode where polysulfide reduction is accelerated and polysulfide diffusion is weakened. (D) Binding geometries and energies of a Li_2_S_4_ molecule on graphene (left, modeled as coronene) and (111) plane of CoS_2_ with cobalt-terminated surface (right), which is derived from theoretical calculation based on DFT. (E) Cycling performance of CoS_2_ (15%) + G-based sulfur cathode at a current density of 2.0 C for 2,000 cycles, followed by 10 cycles at 0.2 C. Reprinted with permission from (Yuan et al., 2016). Copyright 2016, American Chemical Society.

TiS_2_ is one of the earliest intercalating cathode materials applied in secondary lithium batteries (Whittingham, 1976). In 2015, Archer and his team fabricated a 3D S_8_/TiS_2_ hybrid foam as a cathode by thermal reaction, and elemental sulfur was filled into the porous TiS_2_ foam (Figure 12A) (Ma et al., 2015a). With the purpose of understanding the interaction between TiS_2_ and sulfur, DFT calculations were performed. Figure 12B shows the optimized atomic structure of a Li_2_S molecular group attached on the surface of TiS_2_. To maximize the attractive interaction between Li cations and surrounding S anions, two Li atoms were located on the top of the central S atom. Significant interaction between Li_2_S and TiS_2_ was also manifested by a large positive binding energy of 2.60 eV, which was around 10 times higher than that between polysulfide and graphene. This result was also consistent with that of Cui's group, where they applied TiS_2_ as an effective encapsulation material for Li_2_S electrode (Seh et al., 2014). Owing to the synergistic effect between sulfur and TiS_2_ foam, the electronic conductivity and rate performance of the cathode were improved. Consequently, a high areal specific capacity (9 mAh cm^−2^) and high retention ratio were realized.

The pyrite-type CoS_2_ crystal possesses an appreciable electronic conductivity of 6.7 × 10^3^ S cm^−1^ at 300 K and has been proposed as a sulfur host in LSBs. Zhang and co-workers demonstrated that CoS_2_ could provide strong adsorption and activation sites for polar polysulfides, and thus significantly accelerate redox reaction of polysulfides in CoS_2_-incorporated carbon/sulfur cathode (Figure 12C) (Yuan et al., 2016). Compared with the binding energy between graphene and Li_2_S_4_, CoS_2_ and Li_2_S_4_ possessed higher binding energy of 1.97 eV, which was confirmed by DFT calculations (Figure 12D). Because of the enhanced polysulfide redox kinetics, polarization could be mitigated. Meanwhile, increased discharge capacity by 60%, energy efficiency by 10%, and stable cycling performance during 2,000 cycles could also be achieved (Figure 12E).

Other metal sulfides were investigated as well; for instance, Tang et al. studied lithiation/delithiation dynamics of sulfur particles encapsulated by MoS_2_ nanoflakes by using in situ TEM (Tang et al., 2017). They confirmed that MoS_2_ layers on the hollow sulfur nanospheres could limit the volumetric change of the sulfur particles upon lithiation. Moreover, the authors also confirmed that the hermetic encapsulation of sulfur particles by MoS_2_ nanoflakes was effective in restricting the dissolution of polysulfides by physical confinement and chemisorption. This composite electrode showed excellent electrochemical performance with an initial capacity of up to 1,660 mAh g^−1^ at 0.1 C and long-term cycling stability over 1,000 cycles at 1 C.

### Sulfur/Metal Nitride Composites

**Figure 13:**
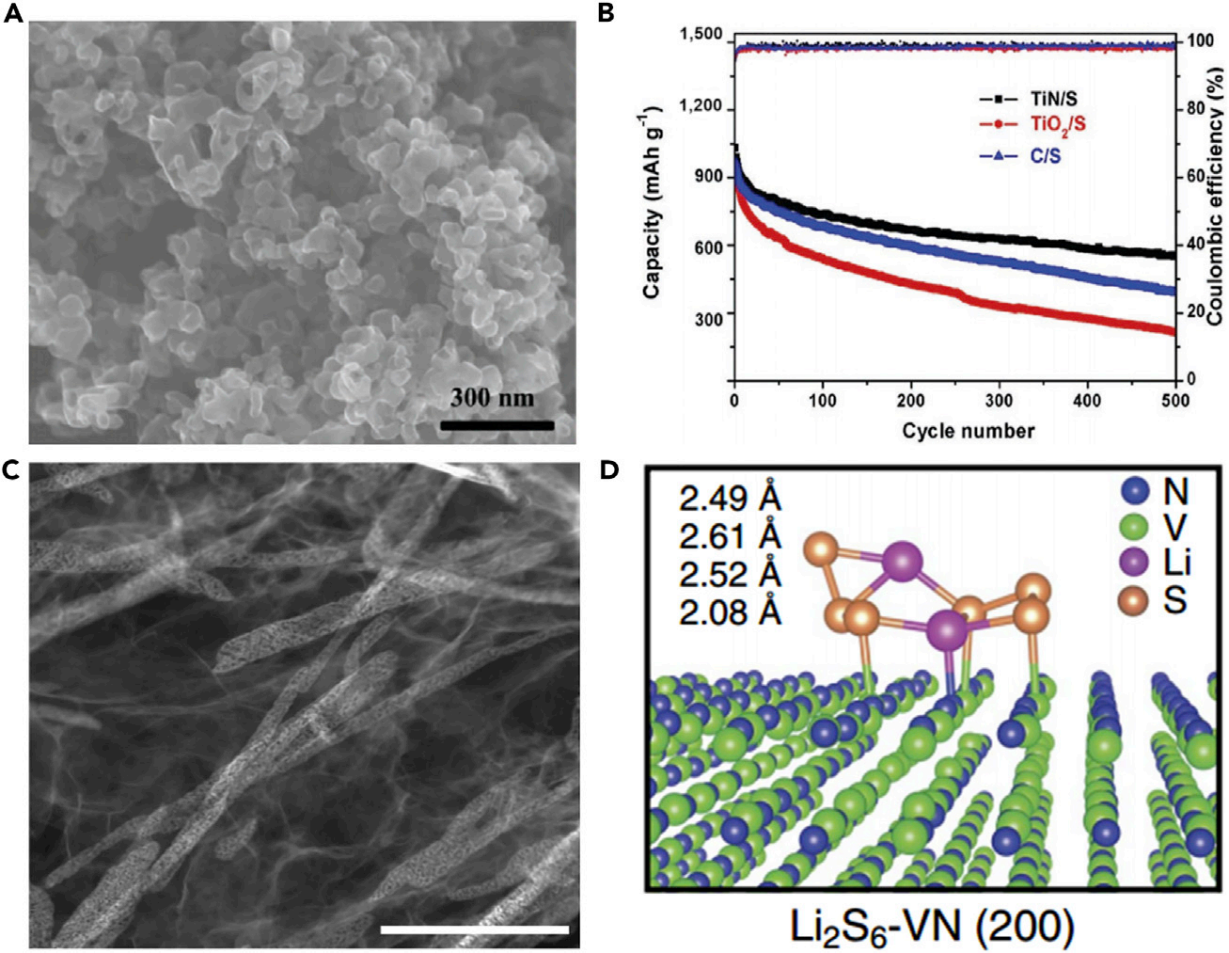
Morphological Characterizations, Performance Investigations, and Theoretical Calculation (A) Scanning electron micrograph of TiN-S. (B) Cycling performance of TiN-S, TiO_2_-S, and Vulcan C-S over 500 cycles at a charge/discharge rate of 0.5 C rate. Reprinted with permission from (Cui et al., 2016). Copyright 2016, Wiley-VCH. (C) High-angle annular dark-field STEM image. Scale bar: 500 nm. (D) Side view of a Li_2_S_6_ molecule on VN (200) surface; the binding energy between Li_2_S_6_ and VN is calculated to be 3.75 eV. Reprinted with permission from (Sun et al., 2017). Copyright 2017, Nature Publishing Group.

Because metal nitrides have some advantages, such as good electronic conductivity (higher than carbon), attractive chemical stability, and strong affinity for polysulfides, they have drawn widespread attention in sulfur cathodes. Despite the positive aspect, there are some intrinsic drawbacks limiting their practical applications, for instance, complex or even uneconomical synthetic process and a poor understanding of the detailed electrochemical reactions of metal nitrides. However, low-cost and green synthetic techniques for the preparation of high-quality metal nitrides need to be explored in the future. In a recent report, Goodenough's research group demonstrated that mesoporous TiN was an excellent host material for LSBs (Cui et al., 2016). The mesoporous TiN was synthesized through a solid-solid phase separation strategy, and the sublimed sulfur was encapsulated in the mesoporous TiN by a melting diffusion method. The scanning electron micrograph of TiN-S composite is shown in Figure 13A. Benefiting from the excellent electronic conductivity, robust porous framework, and advantageous adsorption properties of TiN, TiN-S composite cathode with sulfur content of 58.8 wt% and areal sulfur loading of 1 mg cm^−2^ displayed better cycling stability than the mesoporous TiO_2_-S and Vulcan C-S composite cathodes. Meanwhile, the morphology of TiN-S and a capacity retention of 65.2% could still be kept after 500 cycles (Figure 13B).

Vanadium nitride (VN) possesses a high electronic conductivity (1.17 × 10^6^ S cm^−1^ at room temperature), a strong chemical adsorption for polysulfides that can effectively inhibit the shuttle effect, and catalytic properties that may facilitate redox reaction kinetics. Therefore, VN can be used as a perfect host material in recent researches. For example, Sun et al. fabricated a 3D porous conductive VN/graphene (VN/G) composite accommodating the catholyte as a free-standing cathode material of LSBs (Sun et al., 2017). As shown in Figure 13C, scanning transmission electron microscopic (STEM) image revealed that the VN/G composites were composed of RGO sheets and 3D interconnected network of VN nanoribbons. This composite combined the advantages of both graphene and VN. The free-standing 3D interconnected network of graphene could promote the electron and lithium-ion transportation, and accommodate the volume expansion of sulfur. Moreover, VN showed strong chemical absorption for polysulfides, and could accelerate the redox reaction kinetics. The polar VN and Li_2_S_6_ have strong interaction, which was studied in a dissolved polysulfides system and demonstrated by theoretical calculations (Figure 13D). Owing to the above-mentioned advantages, VN/G composite cathode with a high areal sulfur loading of 3 mg_sul_ cm^−2^ delivered a specific capacity of 1,131 mAh g_sul_^−1^ at 1 C. This work opens new direction of metal nitrides for energy storage.

### Sulfur/Metal Carbide Composites

**Figure 14:**
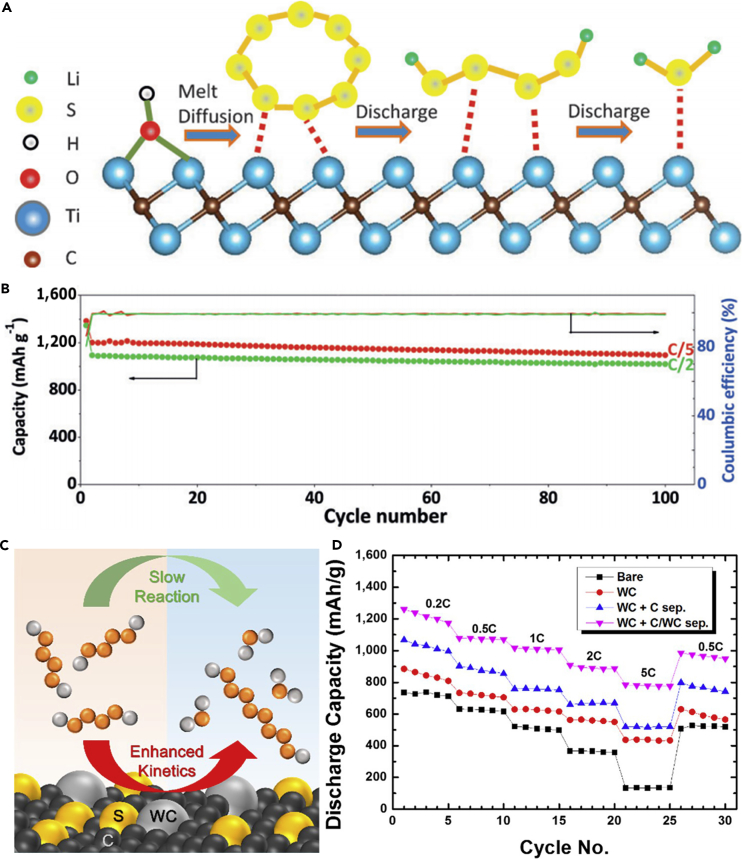
Polysulfides Adsorption, Performance Investigations, and Electrode Kinetics (A) Replacement of Ti-OH bond on MXene surface with an S-Ti-C bond on heat treatment or by contact with polysulfides. (B) Cycling performance of 70S/day Ti_2_C at C/5 and C/2. Reprinted with permission from (Liang et al., 2015a). Copyright 2015, Wiley-VCH. (C) Schematic drawing describing the enhanced kinetics of composite electrode with WC used as additive. (D) Rate capability of the bare electrode, WC electrode, WC electrode with C-coated separator and WC electrode with C/WC-coated separator cells. Reprinted with permission from (Choi et al., 2018). Copyright 2018, American Chemical Society.

Recently, metal carbides have raised widespread concerns due to their excellent electronic conductivity, highly active 2D surface, high melting points, and good mechanical properties. However, metal carbides have the following disadvantages: (1) nano-sized particles of metal carbide agglomerate easily, resulting in poor catalytic activities; (2) it is instable in aqueous media; and (3) it is difficult to grow in preferred orientation. The above-mentioned drawbacks need to be overcome before metal carbides can be used widely. Ti_2_C possesses the above-mentioned advantages. In addition, it possesses ample Lewis acid Ti sites and hydroxyl groups. Nazar's research group reported the use of Ti_2_C as a cathode host to improve the electrochemical performance of LSBs for the first time (Liang et al., 2015a). The composite could chemically absorb polysulfides due to the strong interaction of polysulfide species with the surface Ti atoms (Figure 14A), and the existence of S-Ti-C bonding at the interface demonstrated by X-ray photoelectron spectroscopy studies. As a result, 70S/Ti_2_C composite cathode displayed excellent cycling performance with a specific capacity close to 1,200 mAh g^−1^ at a 5-hour charge/discharge (C/5) current rate (Figure 14B).

In a recent report, Choi et al. reported that tungsten carbide (WC) was used as a cathode additive to enhance the electrochemical performance (reversible capacity and rate capability) of LSBs (Choi et al., 2018). This study demonstrated that WC could offer the strong sulfiphilic surface moieties to entrap dissolved polysulfides. Meanwhile, it could facilitate the chemical disproportionation of intermediate polysulfides, thus enhancing sulfur utilization and restricting polysulfide shuttling (Figure 14C). Based on these superiorities, the composite cathode with WC as an additive displayed excellent electrochemical properties, and a high discharge capacity of 780 mAh g^−1^ could be achieved at a higher C-rate of 5 C (Figure 14D). The materials and methodology will provide a promising way to solve the technical challenges of LSBs.

### Sulfur/Metal Phosphide Composite

**Figure 15:**
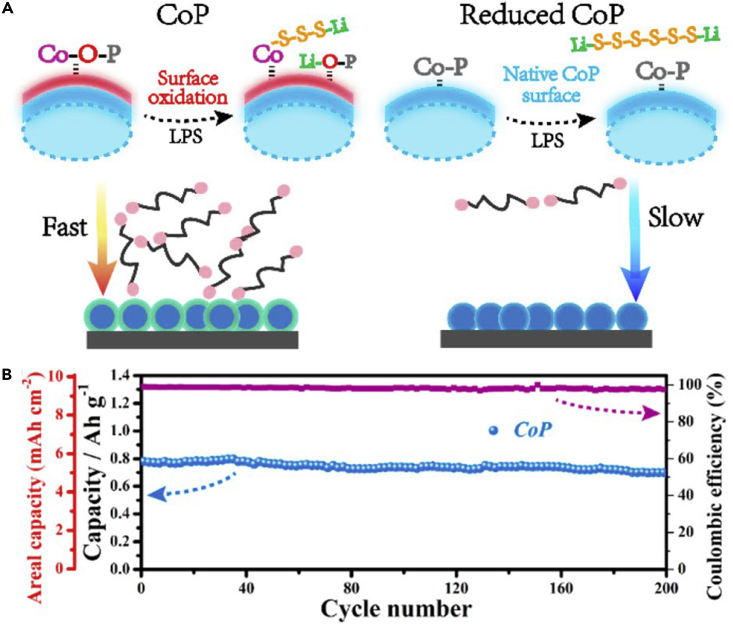
Polysulfides Adsorption and Performance Investigations (A) Schematic illustration of polysulfides adsorption and conversion behavior on the natural versus reduced CoP surfaces. (B) Cycling performance of a high-capacity S cathode (S mass loading: 7 mg cm^−2^; rate: 0.2 C modified with CoP-CNT). Reprinted with permission from (Zhong et al., 2018). Copyright 2018, American Chemical Society.

Apart from the sulfur/metal sulfide, sulfur/metal nitride, and sulfur/metal carbide composite cathode materials discussed above, sulfur/metal phosphide composite has also been investigated as a cathode candidate for LSBs due to its abundance, high activity, and excellent stability. However, the low electronic conductivity is a drawback that limits their practical application. For example, Wang's group reported that CoP nanoparticles could effectively adsorb polysulfides by strong Co-S bonding (Zhong et al., 2018). Co-O-P-like species could be formed on the surface of CoP nanoparticles, resulting in activated Co sites for chemically binding intermediate polysulfides (Figure 15A). Meanwhile, the inner core was beneficial to facilitate electron conduction. However, the pure CoP without the surface oxidation layer hardly bound or adsorbed intermediate polysulfides. As a result, CoP-containing composite cathode with a high sulfur mass loading of 7 mg cm^−2^ delivered a high specific capacity (790 mAh g^−1^) and a stable areal capacity (5.6 mAh cm^−2^) after 200 cycles at 0.2 C (Figure 15B).

### Organosulfur-Based Cathode Materials

**Figure 16:**
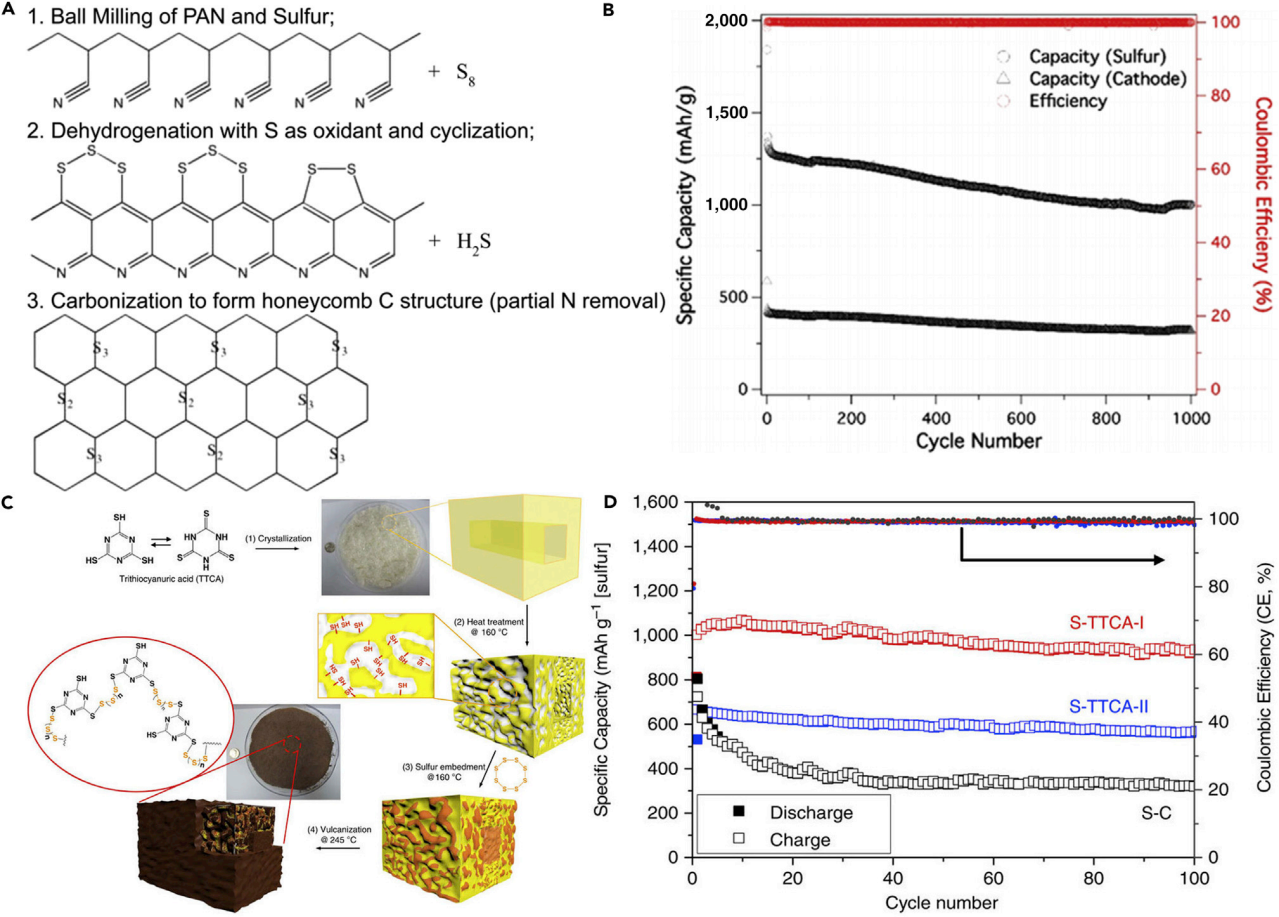
Synthesis Strategy and Performance Investigations (A) Proposed synthesis route for creating sulfur/polyacrylonitrile (SPAN) nanocomposite cathode materials. (B) Capacity and Coulombic efficiency versus cycle number for PANS4. Reprinted with permission from (Wei et al., 2015). Copyright 2015, American Chemical Society. (C) Schematic drawing describing the synthetic procedures of sulfur-rich polymers with controllable morphology. (D) The discharge/charge capacities and Coulombic efficiencies of Li/S-TTCA-I and Li/S-TTCA-II cells, compared with those of conventional Li/S-C cells. Reprinted with permission from (Kim et al., 2015). Copyright 2015, Nature Publishing Group.

Recently, owing to the outstanding processability, flexibility, and broad electrochemical stability window, organosulfur compounds as cathode materials have received extensive attention. Although recent studies on organosulfur cathodes have made great breakthrough in the field of LSBs, the preparation of organosulfur-based cathode material with higher sulfur loading in the large scale is a challenge, which needs to be overcome. Moreover, the electronic conductivity of organosulfur-based cathode needs to be further enhanced. To address multiple, stubborn technical barriers that existed in high-energy LSBs, sulfur/polyacrylonitrile (SPAN) nanocomposite was prepared via a straightforward thermal synthesis process by Archer and his team (Figure 16A) (Wei et al., 2015). The elemental sulfur was strongly linked to polyacrylonitrile (PAN) and used as cathode material for LSBs. Metastable and covalently bound sulfur species S_x_ (x = 2–3) could be obtained by thermal treatment. During cycling, sulfur was maintained as S_3_/S_2_ units covalently attached to a polymer backbone with nitrile groups. SPAN cathode presented long cycling stability with slow capacity decay and high reversible capacity at the end of the 1,000th cycle (Figure 16B). Every sulfur atom in the copolymer composite involved one-electron transfer, which gave this cell a theoretical specific capacity of 837 mAh g^−1^. Moreover, smaller molecular sulfur active species were entrapped in the cathode through covalent bonding and physical confinement in a host, and thus polysulfide dissolution and shuttle were restricted, which led to beneficial electrochemical performance and improved sulfur utilization. SPAN provides a promising route to improve the electrochemical performance of LSBs. However, the specific capacity was low.

In another report, Kim et al. reported a sulfur-containing polymer as an active cathode material to improve the electrochemical performance of LSBs by a new methodology (Kim et al., 2015). Organosulfur cathodes with different morphologies were synthesized by using porous organic crystal templates, which was a unique feature of their system (Figure 16C). The amine groups of trithiocyanuric acid could facilitate fast Li^+^ transport during cycling. As shown in Figure 16D, the organosulfur cathode exhibited a discharge capacity of 945 mAh g^−1^ after 100 cycles at a C-rate of 0.2 C with excellent capacity retention over 92%, and notable rate performance at high current rates of 1 C (872 mAh g^−1^), 3 C (803 mAh g^−1^), and 5 C (730 mAh g^−1^). The size- and shape-controlled soft-template synthesis of organosulfur cathode provides a promising way for advancing LSBs technologies.

### Sulfur-Based Ternary and Quaternary Composite Materials

Sulfur/carbon composites often contact with the electrolyte directly, and result in low utility of the active material and the redox shuttle effect during long charge/discharge process. Carbon materials are nonpolar, only partly inhibit polysulfide dissolution, and act as the conducting additive and backbone to form the conducting network. The polar metal oxides possess good polysulfide adsorption ability, but the electrical conductivity of polar metal oxides is relatively low. The conductive polymers can efficiently prevent the dissolution of lithium polysulfides and improve the electronic conductivity and stability of sulfur cathodes during the electrochemical process. Therefore, the fabrication of multicomposites of sulfur with carbons, conductive polymers, and metal oxides would be an optimized way to enhance the electrochemical performance of LSBs by the synergistic effect of carbons, conductive polymers, and metal oxides.

#### Polymer-Sulfur-Carbon Composite Materials

**Figure 17:**
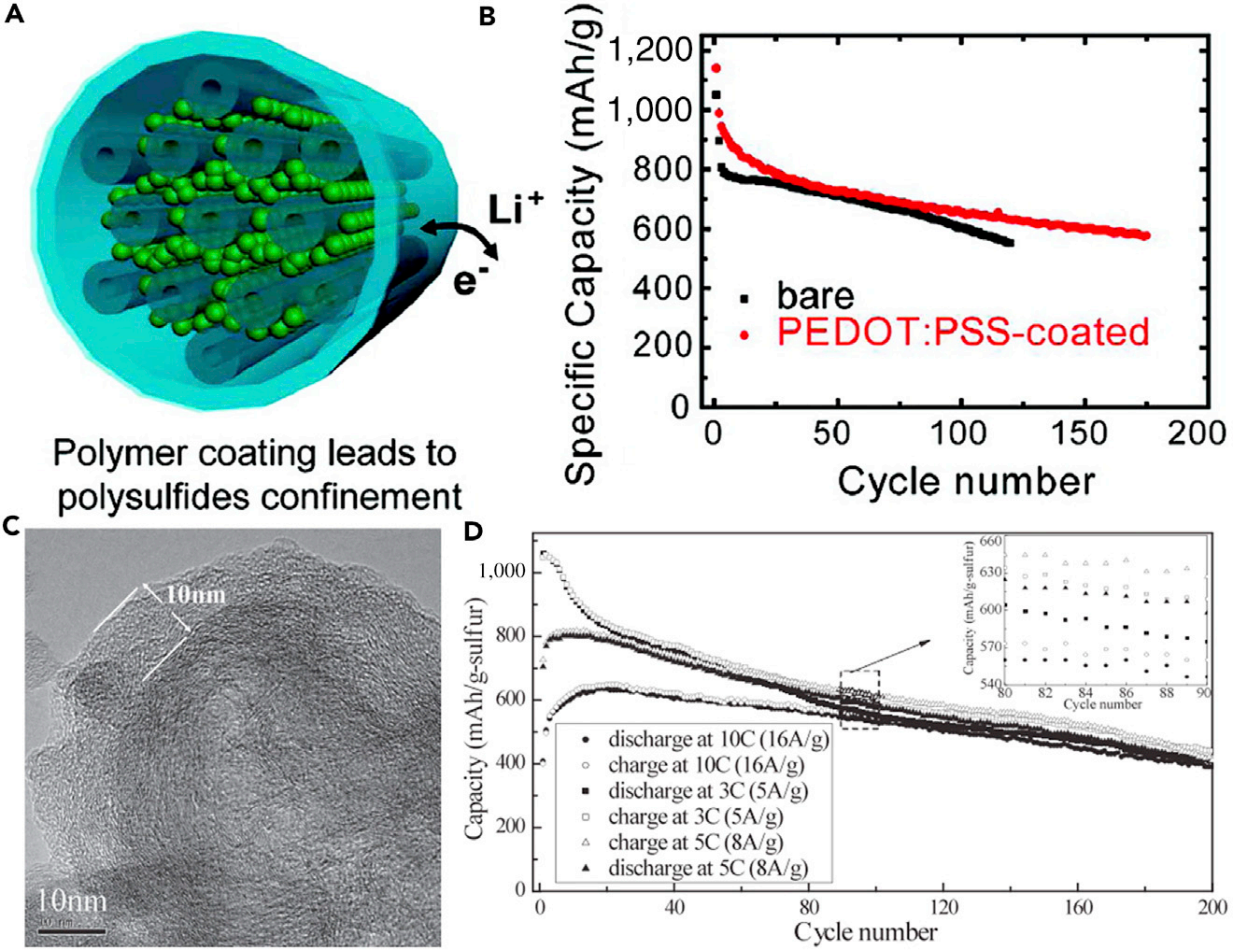
Polysulfides Containment, Morphological Characterizations, and Performance Investigations (A) With conductive polymer coating layer (blue color), polysulfides could be confined within the carbon matrix. (B) Absolute discharge capacity against cycle number. Reprinted with permission from (Yang et al., 2011). Copyright 2011, American Chemical Society. (C) TEM image of PANI@S/C composite with 43.7 wt% S. (D) Cycle performance of PANI@S/C composite with 43.7 wt% S at different rates. Reprinted with permission from (Li et al., 2012). Copyright 2012, Wiley-VCH.

With the purpose of further improving the electrochemical performance of LSBs as mentioned above, more and more researches on a mixed conductive polymer coating with conductive/porous carbon substrates for sulfur-based nanocomposites have been carried out by many groups. For instance, CMK-3 mesoporous carbon/sulfur composite was coated by PEDOT:PSS (Yang et al., 2011). Mesoporous carbon/sulfur composite was prepared by heating well-mixed CMK-3/sulfur at 155°C for 12 hr. PEDOT:PSS coating was beneficial to entrap polysulfides, and thus more polysulfides could be converted to Li_2_S and the loss of active mass in cathode was minimized, which could improve the electrochemical performance of LSBs (Figure 17A). Using PEDOT:PSS-coated CMK-3/sulfur structure as a cathode material, the cycle life and Coulombic efficiency of LSBs were markedly enhanced. In the meanwhile, the composite had demonstrated a high initial discharge capacity, but rapid decay occurred in the subsequent cycles due to the absence of efficient adsorption ability of polysulfides (Figure 17B). In the same context, a rational design of a PANI-coated sulfur/conductive-carbon-black (PANI@S/C) composite with different contents of sulfur was reported (Li et al., 2012). Figure 17C shows the TEM image of PANI@S/C. The sulfur/carbon (S/C) composite was synthesized by a ball-milling method and a subsequent heat treatment of the mixture of conductive carbon black and sublimed sulfur. PANI was coated onto the surface of the as-prepared S/C composite to form the core/shell structure of PANI@S/C composite. PANI@S/C composite with 43.7 wt% S displayed excellent electrochemical properties, and a discharge capacity retention of over 60% was achieved after 200 cycles at an ultrahigh rate of 10 C (Figure 17D). The unique core/shell structure in PANI@S/C composite made a great contribution to the enhanced performance of the cell. It is noteworthy that the two parts had different functions. In particular, the porous carbon black was used as a conductive matrix to improve the conductivity, and the PANI layer suppressed the polysulfide dissolution. Therefore, this synergistic combination of the conductive carbon black and PANI improved the electrochemical performance of a sulfur cathode. However, the cycle stability needs to be further enhanced.

**Figure 18:**
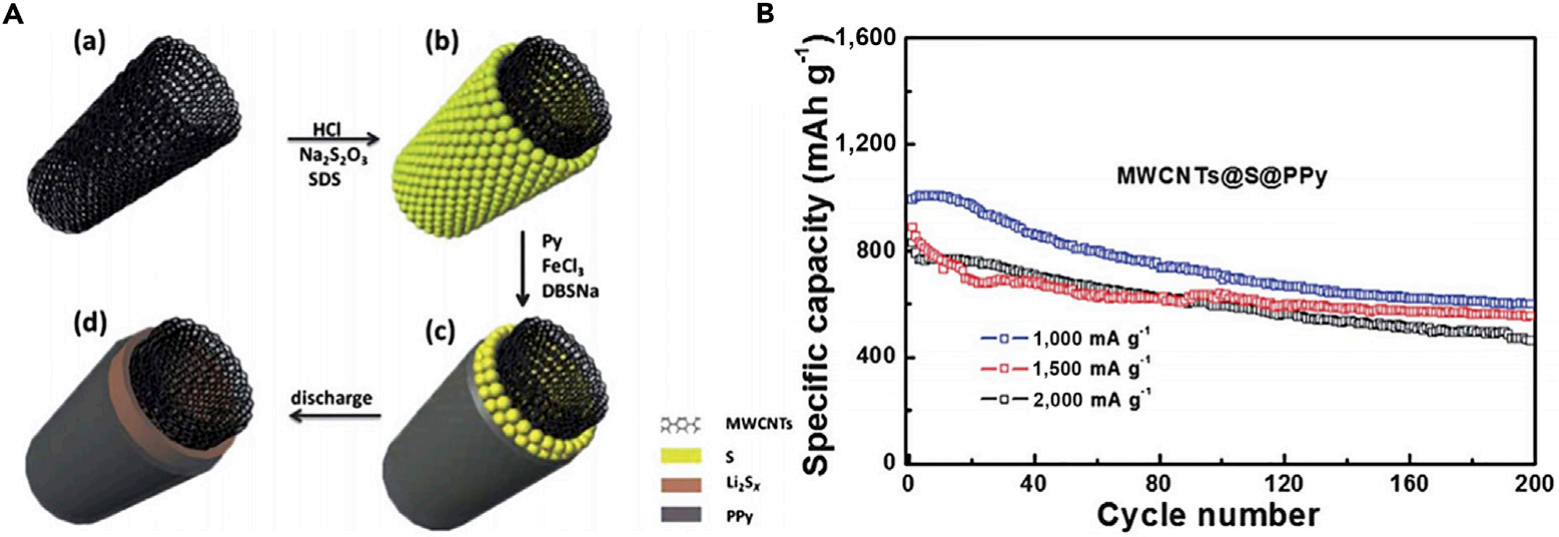
Synthesis Strategy and Performance Investigations (A) Schematic illustration for the synthesis and discharge process of the dual core-shell structured MWCNTs@S@PPy composite. (B) Cycling performance of MWCNTs@S@PPy composite at current densities of 1,000, 1,500, and 2,000 mA g^−1^. Reprinted with permission from (Wang et al., 2013a). Copyright 2013, The Royal Society of Chemistry.

To further enhance the cycle stability, Wang et al. (Wang et al., 2013a) reported a novel MWCNTs@S@PPy composite used as a cathode material for LSBs through a facile one-pot method in 2013 (Figure 18A). The composite possessed a dual core-shell structure. PPy was uniformly coated on the outer surface of MWCNTs@S by chemical oxidation polymerization. Both MWCNTs and PPy were used as the conductive framework to improve the conductivity of sulfur composite. Furthermore, they could provide a fast pathway for electron transport, efficaciously suppress polysulfide dissolution into the organic electrolyte, and mitigate the volumetric expansion of elemental sulfur during cycling process to a certain extent. Benefitting from this unique synergistic effect, the battery based on the as-prepared MWCNTs@S@PPy composite cathode displayed an outstanding rate capability and cyclability, and a high capacity of 560 mAh g^−1^ was maintained after 200 cycles at a higher current density of 1,500 mA g^−1^, implying a good cycle performance (Figure 18B). Owing to their unique architecture, MWCNTs@S@PPy composites demonstrated high specific capacity and long cycle stability over 200 cycles, but still there was significant capacity degradation. That is to say, the shuttling effect of polysulfides still existed during the electrochemical reaction.

**Figure 19:**
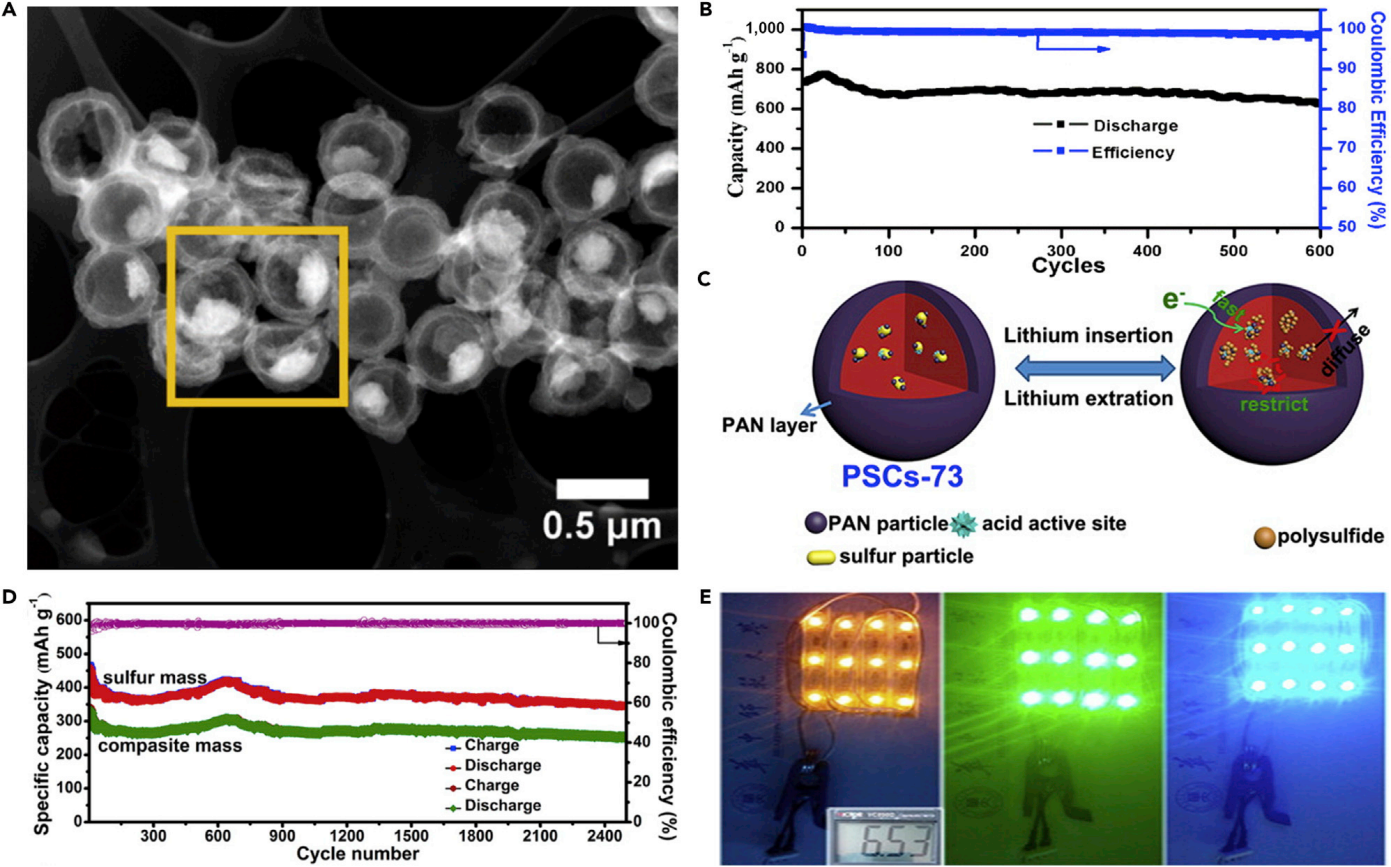
Morphological Characterization, Lithium Insertion-Extraction, and Electrochemical Performance Investigations (A) STEM image of NHC-S composite. The yellow square is the elemental mappings of nitrogen-doped hollow carbon-sulfur in the literature. (B) Discharge capacity and Coulombic efficiency versus cycles for PDA-NHC-S composite at 0.6 C. Reprinted with permission from (Zhou et al., 2014b). Copyright 2014, American Chemical Society. (C) Schematic illustration of the properties of PSCs-73 during lithium insertion and extraction. (D) Cycling performance of PSCs-73 at 5 C. (E) Pictures showing that three lithium batteries in series can light up 12 yellow, green, and blue indicators of 2,835 LED modules (0.96 W). Reprinted with permission from (Hu et al., 2015). Copyright 2015, American Chemical Society.

To better confine the sulfur/polysulfides and improve the cycling stability, a polydopamine-coated hollow carbon-sulfur composite with double-layered core-shell structure was prepared by Zhou et al. (Zhou et al., 2014b) The sulfur species were impregnated into hollow carbon spheres by heat treatment, and then the polydopamine was coated on the hollow carbon-sulfur composite. The sulfur diffused into the inner wall of the hollow carbon, which was demonstrated by STEM image (Figure 19A). The double-layered core-shell architecture efficaciously confined elemental sulfur and lithium polysulfides, and nitrogen-doped hollow carbon could control the size of sulfur core and enhance the conductivity of the cathode. The polymer coating further restricted the elemental sulfur and polysulfides inside the porous carbon shell, and acted as a reservoir to re-utilize these species in the electrochemical reaction. By using this structure, a reversible capacity of 630 mAh g^−1^ and good cyclability could still be maintained at 0.6 C more than 600 cycles (Figure 19B). The sulfur electrodes combined with the silicon film electrodes were applied in the full batteries, and delivered highly improved capacity retention and Coulombic efficiency. However, slight capacity fading still remains in these studies, and the cycle number and sulfur content also need to be improved. Similarly, Hu et al. (Hu et al., 2015) prepared a PAN-assisted S/C nanosphere with high S content (PSCs-73, 73 wt% sulfur) electrode via *in situ* chemical oxidation polymerization successfully. The colloidal carbon sphere with high elastic coefficient was employed to load sulfur and encapsulate dissolvable polysulfide species (Figure 19C). Moreover, the PAN layer on the surface of S/C nanosphere further minimized polysulfide diffusion into the organic electrolyte, and improved the stability of sulfur cathode during electrochemical cycling. Consequently, the designed sulfur cathode demonstrated remarkable cycling stability up to 2,500 cycles at a higher C-rate of 5 C, corresponding to a capacity decay of 0.01% per cycle (Figure 19D), which was a very promising result. Three half-cells were assembled in series. After charging for only 10 min (at 5 C) at 6.53 V, the device could power 12 yellow, green, and blue round light-emitting diode (LED) indicators efficiently (Figure 19E). More importantly, a total of 57 white indicators of LED modules (2.28 W) could be powered by this composite after minutes of charging.

**Figure 20:**
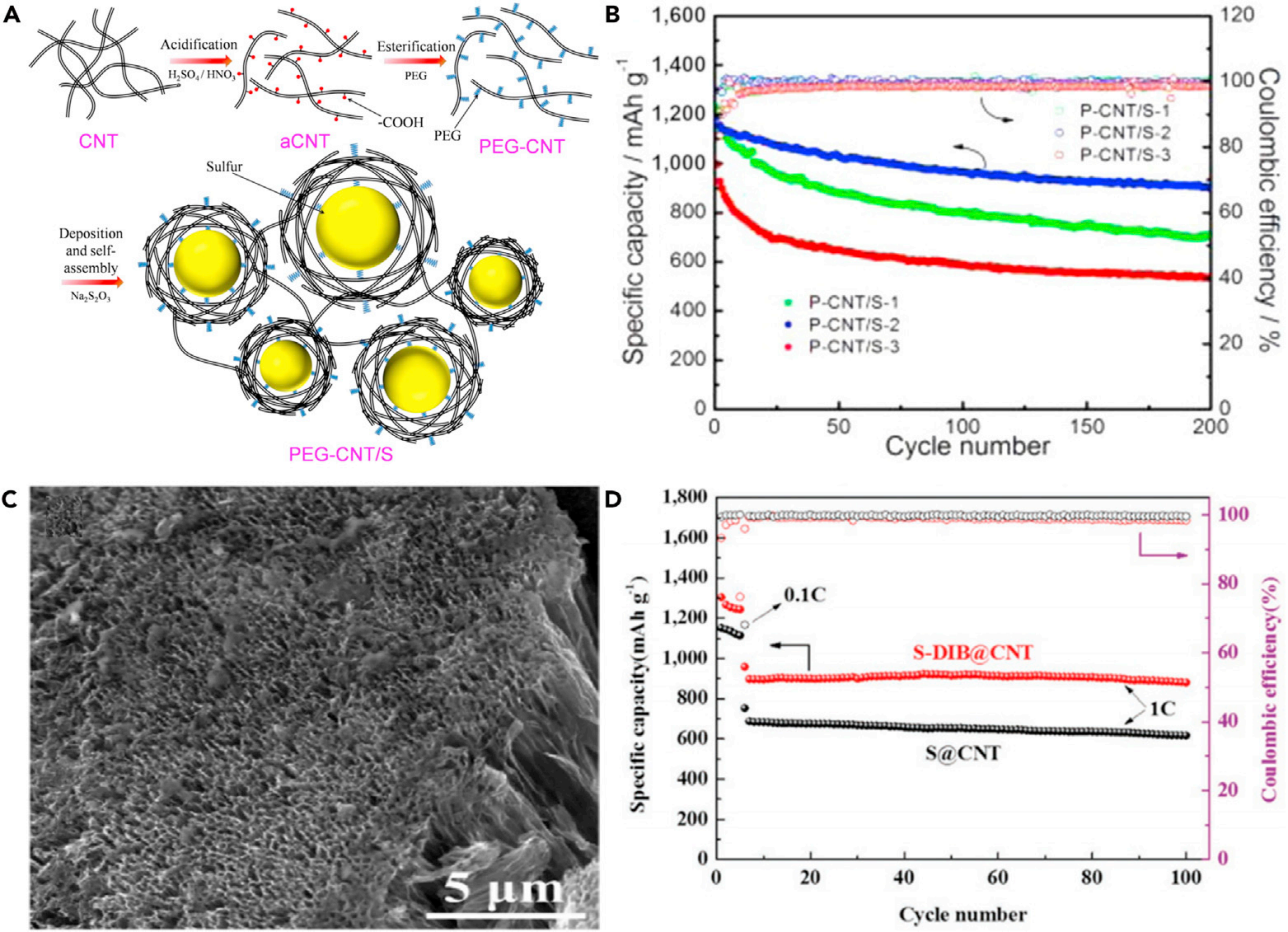
Synthesis Strategy, Morphological Characterization, and Performance Investigations (A) Schematic diagram of the formation of PEG-CNT/S composite. (B) Cycling performance and Coulombic efficiencies of PCNT/S-1, P-CNT/S-2, and P-CNT/S-3 at 0.2 C. Reprinted with permission from (Li et al., 2016b). Copyright 2016, American Chemical Society. (C) Scanning electron micrograph of the as-prepared S-DIB@CNT hybrid. (D) Cycling performance of S@CNT and S-DIB@CNT hybrids at 1 C. Reprinted with permission from (Hu et al., 2016). Copyright 2016, Wiley-VCH.

More recently, for the purpose of further enhancing sulfur content and cycling stability of high-performance LSBs, a novel nest-like PEG-CNT/S composite cathode was successfully synthesized by a facile and simple method with combination process of liquid-phase deposition and self-assembly (Figure 20A) (Li et al., 2016b). The resulting 3D architecture could provide a rapid charge transfer pathway to improve the reaction kinetics, inhibit the notable lithium polysulfide diffusion, and alleviate the volumetric expansion from sulfur particles during charging and discharging cycling process. Taking advantages of this specific structure, the sulfur was successfully confined in 3D architecture and could be clearly observed under electron microscopy. The composite possessed a high and stable sulfur loading. Benefitting from this unique structure design, using PET-CNT/S composite with a sulfur content of 75.9 wt% as a cathode material, an outstanding cycling stability and rate capability was achieved with a stable capacity of 723 mAh g^−1^ after 200 cycles at a higher C-rate of 2 C (Figure 20B), indicative of the high conductivity of the cathode material. However, the capacity fade was still observed during long cycling. With the aim to further improve cycling performance and promote practical application of LSBs, Hu et al. (Hu et al., 2016) fabricated a sulfur-1,3-diisopropenylbenzene@CNT (S-DIB@CNT) membrane hybrid cathode by combining the physical and chemical confinement strategies. The scanning electron micrograph of the as-prepared S-DIB@CNT hybrid is shown in Figure 20C. The sulfur copolymer inside CNT could form C-S bonds with carbon matrix, and hence prevented the dissolution of polysulfides. In particular, the CNT hollow core could efficaciously encapsulate the active material, buffer sulfur volumetric expansion during lithiation, and facilitate fast electron and ion transport. Therefore, both the binder-free and metal current-collector-free electrode delivered a high specific capacity of 880 mAh g^−1^ at 1 C, indicating excellent cycling stability (Figure 20D). This dual confinement strategy offers a new and efficient pathway for the fabrication of high-performance LSBs.

#### Graphene-Sulfur-Carbon Composite Materials

**Figure 21:**
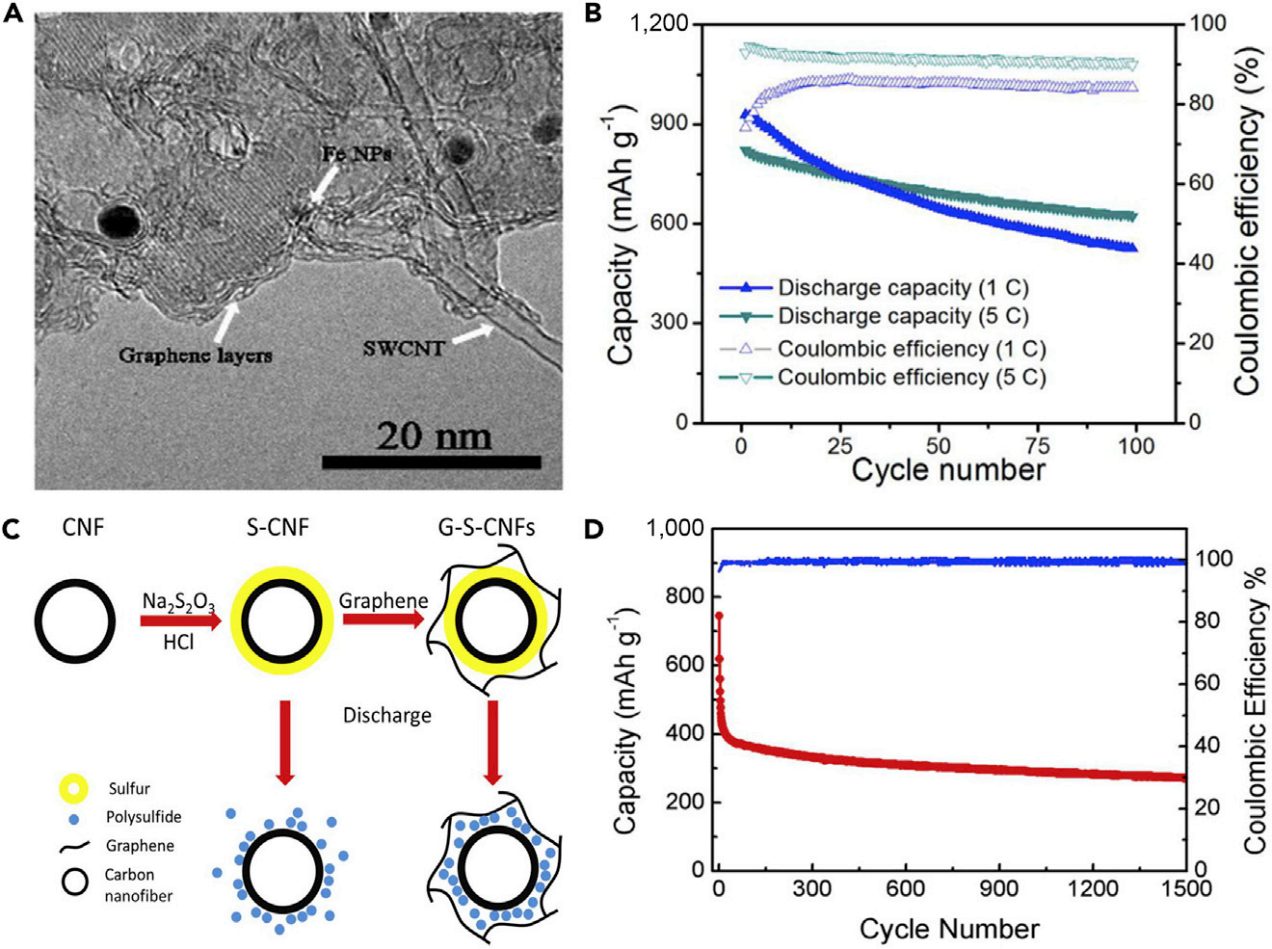
Synthesis Strategy, Morphological Characterization, and Performance Investigations (A) TEM image of the as-grown G/SWCNT/LDO hybrid. (B) Cycling stability of G/SWCNT-S nanocomposites for LSBs. Reprinted with permission from (Zhao et al., 2012). Copyright 2012, American Chemical Society. (C) Schematic illustration of the assembled G-S-CNF multilayered coaxial nanocomposite for improving cathode performance. (D) Cycling performance of the assembled G-S-CNF multilayered coaxial nanocomposite. Reprinted with permission from (Lu et al., 2013). Copyright 2013, American Chemical Society.

In 2012, Zhao et al. (Zhao et al., 2012) prepared a novel graphene/SWCNT (G/SWCNT) hybrid by one-step CVD of methane on FeMgAl layered double hydroxide (LDO) flakes at a high temperature above 950°C. The graphene was deposited onto the LDO surface (Figure 21A), and the thermally stable Fe nanoparticles embedded on the LDO flakes not only catalyzed SWCNT growth but also facilitated the intimate connection between SWCNTs and graphene. The internal spaces between the two stacked graphene layers and among SWCNTs provided ample spaces for sulfur storage and alleviated the volumetric expansion of elemental sulfur during charge/discharge processing. G/SWCNT composite material possessed high surface area, excellent electrical conductivity, and hierarchal porous structure. The conductive agent-free G/SWCNT-S electrodes were fabricated by the typical melting diffusion strategy. Based on the advantages mentioned above, G/SWCNT-S cathode with an S loading amount of 60% displayed outstanding electrochemical performance, and impressive specific capacities of 928 and 650 mAh g^−1^ were achieved even after 100 cycles at higher C-rates of 1 and 5 C, respectively (Figure 21B). However, CVD has high energy resources consumption, and the cycling stability and cycle number also need to be further improved. In a further study, Lu et al. (Lu et al., 2013) fabricated a novel graphene-sulfur-CNFs coaxial nanocomposite as the cathode for LSBs. Element sulfur was coated on the surface of CNFs by the disproportionation reaction of Na_2_S_2_O_3_ with HCl. Graphene and CNF formed a conductive network (Figure 21C). This structure combined the advantages of both CNFs and graphene sufficiently. CNFs not only enhanced the electrical conductivity of the sulfur but also improved the mechanical stability of the cathode, and graphene trapping could effectively immobilize the polysulfides inside the sandwiched structure and accommodate volumetric expansion during the cycling process. Benefitting from this unique synergistic effect, the graphene-sulfur-CNF (G-S-CNF) multilayer and coaxial structure as a cathode material showed increased capacity and long life cycling stability over 1,500 cycles at 1 C (Figure 21D), demonstrating an extremely low decay rate of 0.043% per cycle. In addition, this synthetic route had mild reaction conditions, and thus energy resource consumption was relatively lower than that of CVD.

**Figure 22:**
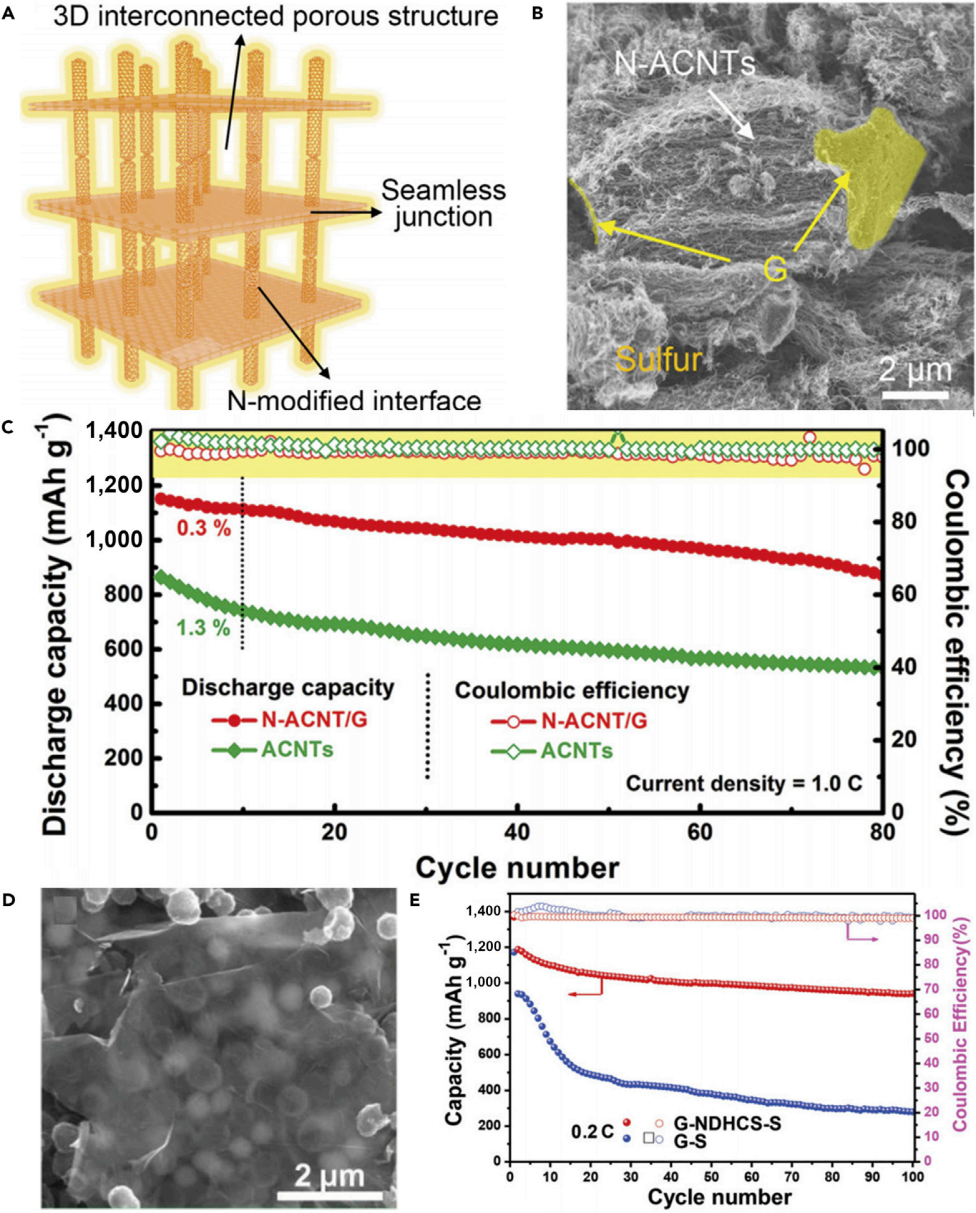
Synthesis Strategy, Morphological Characterization, and Performance Investigations (A and B) (A) Illustration and (B) scanning electron micrograph of N-ACNT/G@S composite. (C) Cycling performance at a current density of 1.0 C. Reprinted with permission from (Tang et al., 2014). Copyright 2014, Wiley-VCH. (D) Scanning electron micrograph of G-NDHCS-S hybrid. (E) Cycling stability and Coulombic efficiencies of G-NDHCS-S and G-S electrodes at 0.2 C for 100 cycles. Reprinted with permission from (Zhou et al., 2015). Copyright 2015, Wiley-VCH.

Similar to the case of porous carbon, the surface of CNT/graphene can be modified by using heteroatoms to improve the affinity for lithium polysulfides as well. Tang et al. (Tang et al., 2014) fabricated nitrogen-doped aligned CNT/graphene (N-ACNT/G) hybrid via a two-step CVD growth, and a metal-embedded bifunctional catalyst was also proposed. Their study demonstrated sp^2^ carbon hybrids for energy storage. Meanwhile, further improvement was achieved as well. Figures 22A and 22B show an illustration and a scanning electron micrograph of N-ACNT/G@S composite cathode, respectively. The sandwich-like hierarchical architecture enabled effective electron transfer pathways and ion diffusion channels to improve the utilization of the active species. Moreover, 3D interconnected mesoporous space contributed to penetration and diffusion of electrolyte. Nitrogen doping could offer more defects and active sites to the carbon framework, and thus enhance the interfacial adsorption for efficient confinement and utilization of sulfur and polysulfides, and the electrochemical performance of LSBs. The electrochemical results showed that the novel N-ACNT/G hybrid with 52.6 wt% sulfur loading as cathode material for LSBs exhibited high sulfur utilization with good cyclability and excellent rate capability (Figure 22C). Instead of using N-ACNT/G hybrids, Zhou et al. (Zhou et al., 2015) prepared a dual-confined flexible cathode by encapsulating sulfur in nitrogen-doped double-shelled hollow carbon spheres (NDHCSs) followed by graphene wrapping. Figure 22D shows the scanning electron micrograph of the G-NDHCS-S hybrid. The porous double-shelled hollow structure of NDHCSs is in favor of enhancing the sulfur content, accommodating the volumetric expansion of sulfur cathode during the charge/discharge process, and inhibiting the dissolution of intermediate polysulfides into the electrolyte. A high electrical/ionic conductive network was offered by graphene wrapping, and nitrogen doping could help to trap the polysulfides. Furthermore, the well-built 3D carbon conductive network did not require binders, which contributed to improving the capacity and long cycle life. Owing to its highly optimized structure, it maintained excellent rate capability and high reversible capacity even at a high current density of 2 C (Figure 22E).

**Figure 23:**
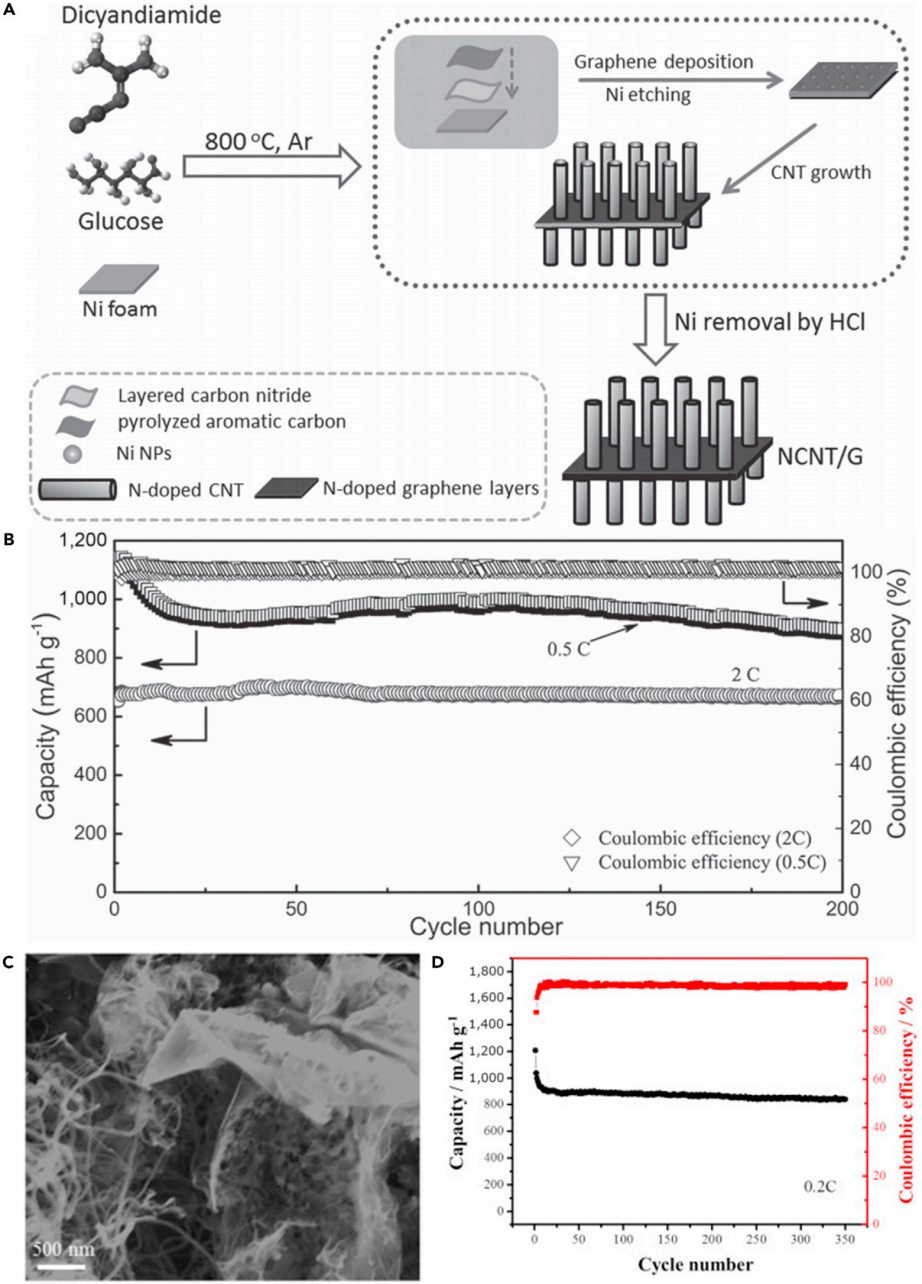
Synthesis Strategy, Morphological Characterization, and Performance Investigations (A) Schematic diagram of NCNT/G hybrid fabricated by a facile solid-state growth method. (B) Cycling performance of NCNT/G@S electrode at 0.5 and 2 C. Reprinted with permission from (Ding et al., 2016). Copyright 2016, Wiley-VCH. (C) Scanning electron micrograph of CNT-g-Gr. (D) Long-term cycle life of S@CNT-g-Gr at 0.2 C. Reprinted with permission from (Wang et al., 2017). Copyright 2017, Elsevier.

To achieve superior properties in energy conversion and storage, highly nitrogen-doped CNT-graphene 3D nanostructures were fabricated through a facile solid-state growth strategy without the necessity to use CVD by Yu and co-workers (Figure 23A) (Ding et al., 2016). The as-prepared NCNT/G composite possessed robust, porous, and well-interconnected 3D architecture, which was beneficial to fast electron transport and lithium diffusion. Specifically, the unique 3D architecture remarkably buffered volumetric change during the charge/discharge cycling, and high nitrogen doping and physical confinement of the as-prepared composite could enhance utilization of sulfur. Consequently, this structure exhibited excellent electrochemical performance when used as a cathode material for LSBs. The developed NCNT/G@S hybrid delivered high improvement in the reversible capacity (e.g., 1,314 and 922 mAh g^−1^ at different current rates of 0.2 and 1 C, respectively). In the meanwhile, at a higher C-rate of 2 C, it displayed long life cycling stability over 200 cycles (Figure 23B), implying their great potential for energy storage application. Compared with other studies mentioned above, this research utilizes a synthesis method with energy saving and environmental protection. This work not only improves the electrochemical performance of LSBs but also reduces energy consumption. For the same purpose, Wang et al. (Wang et al., 2017) fabricated a CNT-grafted-graphene (CNT-g-Gr) through CNT growth on Ni-deposited graphene sheet, which possessed high electric conductivity and polysulfide adsorption capability. The scanning electron micrograph of CNT-g-Gr is shown in Figure 23C. The *in situ* formation of Ni nanoparticles on graphene sheet could act as catalytic sites for CNT growth and the anchor sites for polar lithium polysulfide adsorption. The superb polysulfide adsorption capability of the hybrid was attributed to the synergistic effect of graphene, CNT absorbing weakly polar Li_2_S_6_, and Ni nanoparticles absorbing strongly polar Li_2_S_4_. Due to its well-designed structure, it displayed long life cycling stability over 350 cycles with a high capacity of 800 mAh g^−1^ at a current rate of 0.2 C (Figure 23D).

#### Metal Oxide-Sulfur-Carbon Composite Materials

**Figure 24:**
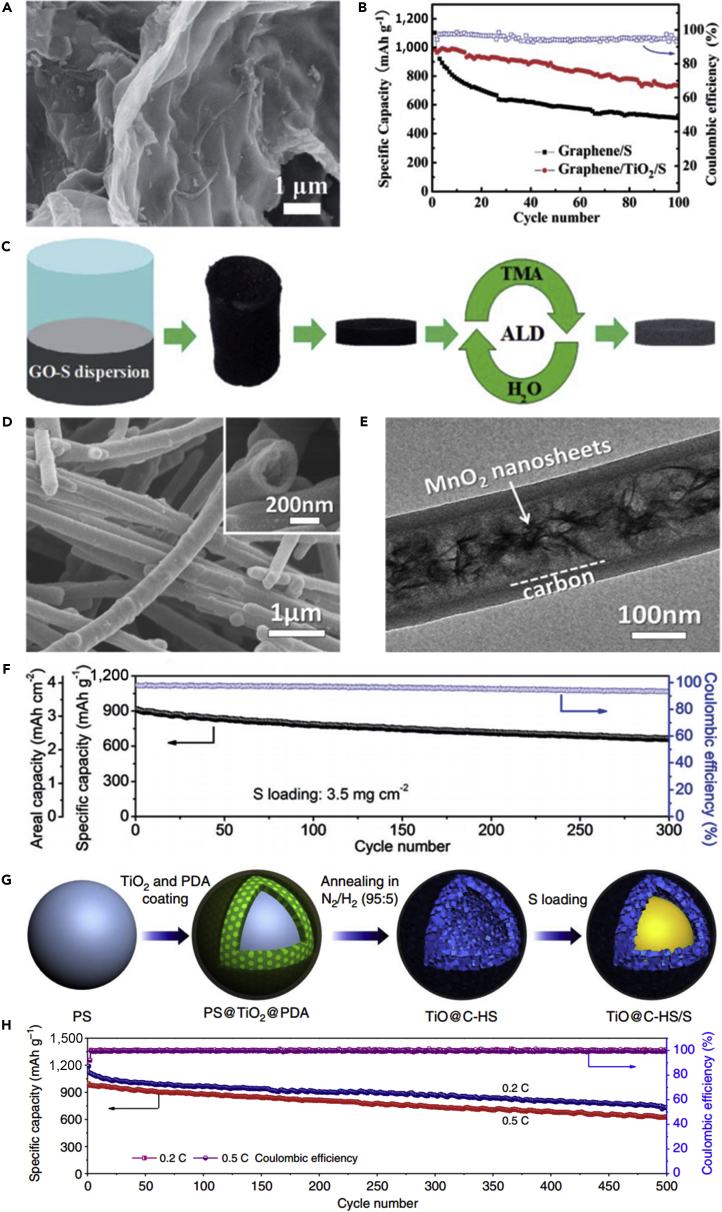
Synthesis Strategy, Morphological Characterization, and Performance Investigations (A) Scanning electron micrograph of graphene/TiO_2_/S nanocomposite. (B) Cycling performance of graphene/S and graphene/TiO_2_/S electrodes at a current rate of 0.5 C. Reprinted with permission from (Ding et al., 2013). Copyright 2013, The Royal Society of Chemistry. (C) Schematic illustration of the preparation of G-S composite coated with Al_2_O_3_ by ALD. Reprinted with permission from (Yu et al., 2014). Copyright 2014, The Royal Society of Chemistry. (D and E) FESEM and TEM images of MnO_2_@HCF. (F) Prolonged cycling performance of MnO_2_@HCF/S at 0.5 C and the corresponding Coulombic efficiency. Reprinted with permission from (Li et al., 2015). Copyright 2015, Wiley-VCH. (G) Schematic illustration of the synthesis process of TiO@C-HS/S composite. (H) Prolonged cycle life and Coulombic efficiency of TiO@C-HS/S electrode at 0.2 and 0.5 C. Reprinted with permission from (Li et al., 2016f). Copyright 2016, Nature Publishing Group.

To design an efficient host to confine sulfur and promote the electrochemical reaction, a sandwich structured graphene/TiO_2_/S electrode was fabricated by Zhang and co-workers (Ding et al., 2013). Elemental sulfur was confined in a TiO_2_ nanocrystal (3–5 nm)-decorated graphene nanosheet host. Figure 24A is a scanning electron micrograph of graphene/TiO_2_/S nanocomposite. The graphene/TiO_2_/S composite electrode with a sandwich structure exhibited improved electrochemical properties with a high initial specific capacity of about 985 mAh g^−1^ at 0.5 C and capacity retention of up to 75% after 100 cycles (Figure 24B). The electrolyte could rapidly diffuse, and the volumetric change of sulfur was also alleviated in the sandwich structure. Moreover, pore adsorption of the graphene/TiO_2_ host and the adsorption of TiO_2_ nanocrystals could efficaciously prohibit lithium polysulfide dissolution. *In situ* formed Li_x_TiO_2_ worked together with the highly conductive graphene layer to facilitate fast Li^+^/e^−^ transport. The advantages mentioned above are responsible for the enhanced electrochemical performance. Similarly, Al_2_O_3_ could be used as a portion of the cathode for improving electrochemical performance. For example, Yu et al. (Yu et al., 2014) prepared a graphene-sulfur (G-S) composite via a facile hydrothermal process. The composite was modified with an atomic layer deposition (ALD)-Al_2_O_3_ coating, and used as a cathode material for LSBs (Figure 24C). Compared with a bare G-S composite electrode, the battery based on G-S composite cathode with an ALD-Al_2_O_3_ coating exhibited better electrochemical properties. At a high C-rate of 0.5 C, high specific capacity of 646 mAh g^−1^ could be also achieved, with 82% capacity retention after 100 cycles. ALD-Al_2_O_3_ coating could prohibit the dissolution of polysulfide intermediates to electrolyte and alleviate the shuttle effect. Hence, G-S composite electrode showed excellent rate capability and reversibility. Although the electrochemical properties were improved to some extent, the cycling stability and sulfur content need to be further enhanced.

Instead of using graphene, Lou's group reported a 1D composite nanoarchitecture, in which hollow CNFs were filled with MnO_2_ nanosheets (MnO_2_@HCF) as highly efficient host for LSBs (Li et al., 2015). Figures 24D and 24E show the field-emission scanning electron microscopy (FESEM) and TEM images of MnO_2_@HCF, respectively. MnO_2_@HCF hybrid host could facilitate electron and ion transfer and prevent polysulfide dissolution. In detail, 1D nanofibers could form a 3D interconnected conductive network, and hence reduce the resistance of electron and ion transport during charging/discharging cycling. The birnessite-type MnO_2_ nanosheets could chemically bind polysulfides and efficaciously suppress the dissolution. The battery assembled with MnO_2_@HCF/S composite with 71 wt% sulfur and an area sulfur loading as high as 3.5 mg cm^−2^ exhibited high specific capacity and excellent cycling stability. A remarkable reversible capacity of almost 662 mAh g^−1^ (2.3 mAh cm^−2^) at 0.5 C after 300 cycles stood out (Figure 24F).

Recently, a sulfur host based on highly conductive polar titanium monoxide@carbon hollow nanospheres was designed and synthesized for LSBs by Lou's group (Figure 24G) (Li et al., 2016f). This sulfur host could enhance the conductivity of the sulfur cathode and alleviate the dissolution of intermediate polysulfide products at the same time. DFT calculation indicated that rock-salt structured stoichiometric TiO could provide stronger chemical adsorption energies for Li_2_S_x_ than TiO_2_. TiO@C shells possessed excellent conductivity and strong lithium polysulfide adsorption capability. Meanwhile, it could also enhance the redox reaction kinetics of sulfur species during the electrochemical reaction. Therefore, using TiO@C/S composite as a cathode for LSBs, excellent long-term cycling performance was achieved. Owing to its highly optimized structure, impressive specific capacities of 750 and 630 mAh g^−1^ were maintained at C-rates of 0.2 and 0.5 C after 500 cycles, respectively (Figure 24H). The high Coulombic efficiency of >99% was obtained during the charging/discharging process. Even the areal loading of sulfur was as high as 4.0 mg cm^−2^; high areal capacities could be attained at various current densities. The outward diffusion of lithium polysulfides was prevented by using polar shells, which broke the limitation of chemically bonding polysulfides on the surfaces of the polar host. This indicated the successful strategy in restricting the notable lithium polysulfide diffusion, and consequently resulting in the prominently improved electrochemical performance of LSBs.

#### Sulfur-Based Quaternary Composite Materials

**Figure 25:**
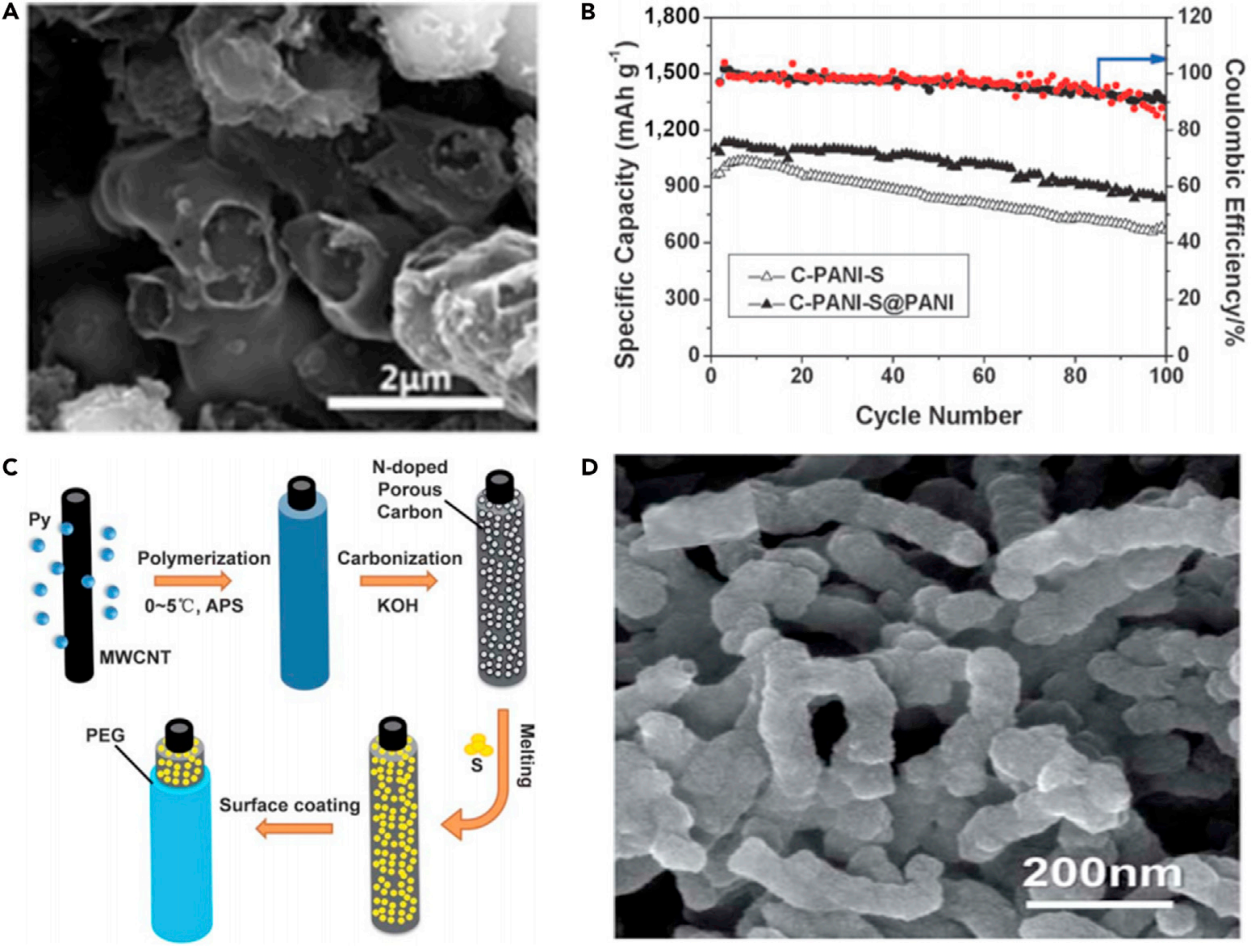
Synthesis Strategy, Morphological Characterization, and Performance Investigations (A) Scanning electron micrograph of C-PANI-S@PANI composite. (B) Cycling performance of C-PANI-S and C-PANI-S@PANI composites and Coulombic efficiency of C-PANI-S (black) and C-PANI-S@PANI (red) composites at 0.2 C. Reprinted with permission from (Wang et al., 2013b). Copyright 2013, The Royal Society of Chemistry. (C) Synthesis procedure of MWCNT@S/NPC@PEG composite. (D) Scanning electron micrograph of MWCNT@S/NPC@PEG. Reprinted with permission from (Li et al., 2014c). Copyright 2014, The Royal Society of Chemistry.

In 2013, to improve the utilization of cathode material and the sulfur loading, Wang et al. (Wang et al., 2013b) synthesized a multi-core-shell structured C-PANI-S@PANI composite with sulfur content up to 87%, and Figure 25A depicts its scanning electron micrograph. The composite showed enhanced conductivity, and the diffusion of intermediate polysulfides was also inhibited. The pores on the composite provided channels for ion diffusion and electrolyte infiltration. The surface coating of PANI on C-PANI-S composite was more effective at improving the capacity and active sulfur utilization, and accommodating volumetric change produced by sulfur discharge products. Consequently, the battery assembled with C-PANI-S@PANI composite presented higher specific capacity and excellent cycle stability. When the sulfur loading of the cathode was above 6 mg cm^−2^, a specific capacity of 835 mAh g^−1^ could be achieved with 76% capacity retention after 100 cycles at 0.2 C (Figure 25B). In the meanwhile, a high Coulombic efficiency of approximately above 90% could still be achieved during 100 cycles. However, faster capacity decay was the main issue for flexible cathodes. The following year, Li et al. (Li et al., 2014c) designed a novel MWCNTs@S/NPC@PEG composite with a coaxial nanocable structure as a cathode material for high-rate LSBs (Figure 25C). MWCNT@NPC composite was prepared via KOH-assisted carbonization of MWCNT@PPy. Elemental sulfur was added into the MWCNT@NPC composite by a melting diffusion strategy, and thus the MWCNT@S/NPC composite cathode was fabricated. The scanning electron micrograph of MWCNT@S/NPC@PEG is shown in Figure 25D. The middle hierarchical porous nitrogen-doped carbon (NC) capsule could entrap the sulfur particles and further enhance the electrical conductivity of the cathode; the inner MWCNT matrix and the outermost restrictive PEG sheath could ensure fast electronic transport and suppress the dissolution of polysulfides generated in the electrochemical reaction, respectively. The developed MWCNT@S/NPC@PEG coaxial nanocable structure delivered high improvement in reversible capacity (e.g. 791, 551, and 400 mAh g^−1^ at different rate densities of 0.5, 2, and 5 C, respectively), indicating excellent high-rate capability.

**Figure 26:**
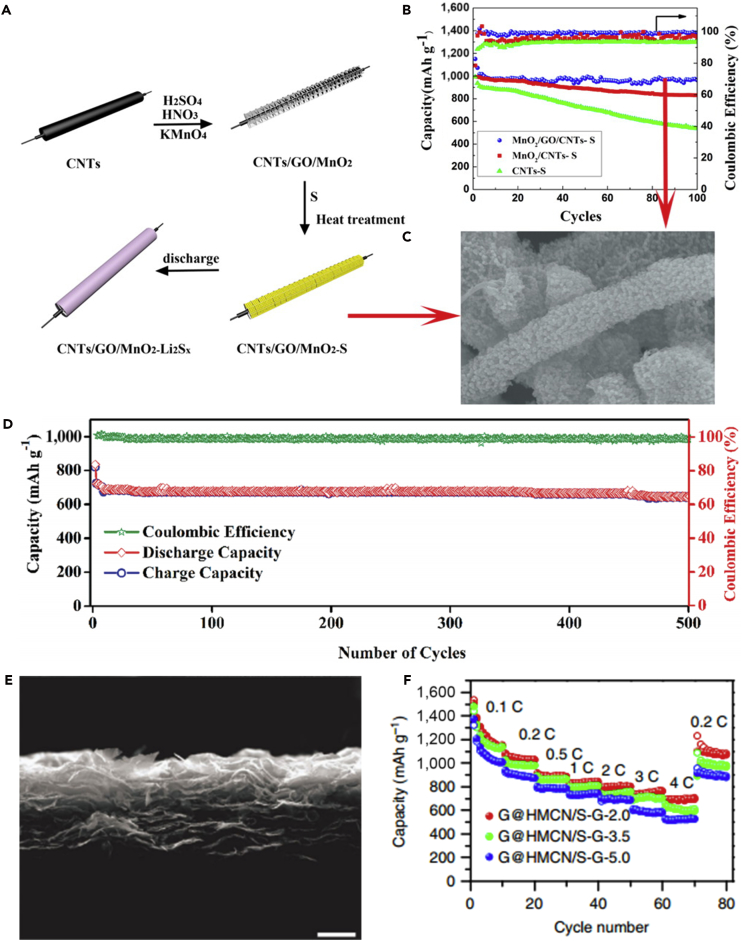
Synthesis Strategy, Morphological Characterization, and Performance Investigations (A) Schematic illustration of the synthesis and discharge process of the 3D-structured MnO_2_/GO/CNTs-S composite. (B) Comparison of cycling stability and Coulombic efficiencies of CNT-S, MnO_2_/CNTs-S, and MnO2/GO/CNTs-S composites at 0.2 C. (C) Scanning electron micrograph of MnO_2_/GO/CNTs-S composite. Reprinted with permission from (Li et al., 2016c). Copyright 2016, American Chemical Society. (D) Cyclic performance and Coulombic efficiency of Si/SiO2@C-S hybrid spheres with 15.5 wt% Si/SiO_2_ content. Reprinted with permission from (Rehman et al., 2016). Copyright 2016, Wiley-VCH. (E) Cross-sectional scanning electron micrograph of the flexible G@HMCN paper. Scale bar: 5μm. (F) Rate capabilities of G@HMCN/S-G-2.0, G@HMCN/S-G-3.5, and G@HMCN/S-G-5.0. Reprinted with permission from (Pei et al., 2017). Copyright 2017, Nature Publishing Group.

In 2016, with the purpose of improving cycling stability and promoting commercial application of LSBs, Li et al. (Li et al., 2016c) fabricated a MnO_2_/GO/CNTs-S composite with a unique 3D architecture by a one-pot chemical method and heat treatment approach (Figure 26A). In such a composite, the innermost 1D CNTs offered a conducting network. 2D petal-like ultrathin MnO_2_/GO nanosheets were anchored on the sidewalls of CNTs, and possessed high specific surface area and highly efficient polysulfides adsorbents, and hence could suppress the shuttling effect. It could also afford adequate space for sulfur loading. The outermost nano-sized sulfur particles were distributed uniformly onto the surface of MnO_2_/GO/CNTs. The scanning electron micrograph of MnO_2_/GO/CNTs-S composite is shown in Figure 26C. The as-prepared MnO_2_/GO/CNTs-S cathode displayed excellent comprehensive performance. As shown in Figure 26B, MnO_2_/GO/CNTs-S composite delivered an initial discharge capacity of 1,150 mAh g^−1^, and more importantly, it was able to maintain a stable cycling performance for 100 charge/discharge cycles at 0.2 C when the area density of sulfur was 2.8 mg cm^−2^. Moreover, MnO_2_/GO/CNTs-S composite was easily synthesized on a large scale, and hence might be a promising candidate for commercial application. In the same year, to solve the hurdle of the intermediate polysulfide dissolution, a silicon/silica (Si/SiO_2_) cross-link with hierarchical porous carbon spheres (Si/SiO_2_/C) composite was designed simply via carbonization of single precursor (Rehman et al., 2016). This well-designed carbon nanoarchitecture could adsorb lithium polysulfides via physical and chemical adsorption, and thus inhibit the polysulfide shuttle effect. The cross-link network of Si/SiO_2_ could suppress lithium polysulfide dissolution, and simultaneously enhance the utility of active material. The micro-mesoporous graphitic carbon could improve sulfur loading and facilitate an easy access to Li^+^ ingress/egress in the electrochemical process. This structure could better confine sulfur and polysulfide species. When the content of Si/SiO_2_ was 15.5 wt% in Si/SiO_2_@C-S hybrid sphere, a high mass sulfur loading of 69.6 wt% was obtained, and the hybrid sphere showed optimized electrochemical performance. Due to its unique porous carbon sphere structure, it maintained excellent rate capability even at a high current density of 2 C, high reversible capacity, and ultraslow capacity decay of 0.063% per cycle during 500 cycles, indicating excellent cycling performance (Figure 26D).

Very recently, the high-energy-density LSBs have received increasing attention. However, the electrochemical performance degradation of high-sulfur-loading cathodes is a crucial problem for practical application of LSBs at present. Moreover, the volumetric capacities of high-sulfur-loading cathodes need to be further improved as well in future research. To solve these issues, a 2D carbon yolk-shell nanosheet was successfully designed and synthesized by a facile and reliable hard-templating method (Pei et al., 2017). The scanning electron micrograph of the composite is shown in Figure 26E. This nanomaterial possessed the structural characteristics of high surface area, nitrogen doping, unique 2D structure, and high dispersibility. The highly porous graphene@hollow mesoporous carbon (G@HMCN) nanosheet could dramatically enhance sulfur content. The abundantly doped nitrogen could chemically adsorb the polysulfides generated in the electrochemical reaction. Graphene in the self-supporting carbon/sulfur cathode could improve the overall electrical conductivity of the cathode material, which led to the fast Li^+^/e^−^ transport and thus improved the electrochemical performance. Based on the structural advantages of G@HMCN, a novel self-supporting sulfur cathode with high sulfur loading and sulfur content was constructed by the co-assembling of G@HMCN/S composite and graphene. Owing to its highly optimized structure, sulfur utilization was improved greatly at high sulfur loading, which was reflected by the enhanced capacity and prolonged cycling life. G@HMCN/S-G cathode with sulfur loading of 5 mg cm^−2^ and sulfur content of 73 wt% exhibited a high capacity, good rate performance, and excellent cycling stability accompanied with the favorable balance between the areal (5.7 mAh cm^−2^) and volumetric capacities (1,330 mAh cm^−3^) (Figure 26F). More importantly, an areal capacity of 11.4 mAh cm^−2^ could be further achieved by increasing the sulfur loading of G@HMCN/S-G to 10 mg cm^−2^. The superb electrochemical performance suggested the successful design concept of novel 2D carbon yolk-shell nanosheet with high surface area, nitrogen doping, and hierarchical pore distribution.

### Conclusions and Outlook

In this review, we comprehensively and systematically discuss the recent development in sulfur/carbons, sulfur/metal oxides, sulfur/conductive polymers, sulfur/metal sulfides, sulfur/metal nitrides, sulfur/metal carbides, sulfur/metal phosphides, organosulfur-based cathode materials, and other ternary and quaternary composite materials for LSBs. LSBs are considered to be one of the most promising candidates for next-generation high-performance lithium batteries and are superior to the routine LIBs. According to previous analysis, LSBs can be comparable to LIBs when the areal capacity reaches 4.0 mAh cm^−2^. To achieve the goal of a battery with a specific energy of 350 Wh kg^−1^ for Li-S pouch batteries, the following parameters need to be considered carefully (Peng et al., 2017). First, the sulfur content needs to be over 75 wt%. Second, a capacity of at least 900 mAh g^−1^ on the strength of the entire cathode is required. Third, areal sulfur loading must be more than 5 mg cm^−2^. It is worth noting that the performance of LSBs is still far from reaching the applied energy density due to some disadvantages, such as the insulating nature of sulfur and the discharge product Li_2_S, polysulfide dissolution causing active sulfur loss, rapid capacity fading, and the large volumetric expansion/contraction in the conversion reaction. Therefore, currently, LSBs are very far from reaching commercial deployment. Computational chemistry is a significant branch of modern science. Various methods (DFT, molecular dynamics, Hartree-Fock-based models, post-Hartree-Fock methods, quantum mechanics/molecular mechanics, etc.) have been developed. The relevance of the above-mentioned model systems will be improved by high-accuracy calculations and large-scale models. For the purpose of predicting, screening, and optimizing materials at an unprecedented scale and rate by integrating computational, experimental, and data science methodologies, and thus instructing future experiments, the concept of materials genome is proposed in recent time. Because systematic, organized, large, and robust data are not available, machine learning approaches have not been applied to LSBs. Therefore, a well-established database is urgently needed for the future development of LSBs, and new materials and synthetic methodology need to be further explored by theoretical simulations. Although important progress has been made on LSBs, we believe that great advances still await discovery. To improve the electrochemical performance of LSBs and achieve its commercial application, the choice of host materials for sulfur cathode is very important. Herein, we outline several possible directions for future studies of LSB cathode materials, which may lead to pathways for practical application of LSBs.

1. Pure sulfur is incorporated with carbon or conductive polymer hosts to increase electronic conductivity of sulfur (5 × 10^−30^ S cm^−1^ at room temperature) to >10^−4^ S cm^−1^ (commercial LiCoO_2_, ∼10^−4^ S cm^−1^) and entrap lithium polysulfides by physical adsorption, chemical adsorption, and coating. The electrochemical performance of LSBs is significantly improved by using these carbon and polymeric matrices.
2. The metal oxides are commonly used as inorganic encapsulation materials; however, researches on metal nitrides and sulfides remain relatively less. Moreover, the application of some metal sulfides with catalytic properties is also appealing in this respect. They can combine the advantages of strong affinity for polysulfides and high electronic conductivity in the development of high-performance LSBs.
3. The transition-metal phosphides are even better at stabilizing sulfur; how they chemically interact with polysulfides at the solid/liquid interface remains poorly understood, which is preventing molecular-level understanding of LSBs' surface chemistry and the rational design of high-performance cathode materials for LSBs.
4. MXenes possess high metallic conductivity, highly active 2D structure, strong polysulfide-anchoring sites, and good mechanical properties, and are exciting host materials of sulfur. They can be produced by selectively etching “A” element from MAX phases (layered carbides or nitrides, where M is a transition metal, A is a group IIIA or IVA element, and X is C and/or N). However, this synthetic method with low yield is dangerous. Therefore, developing moderate and safe synthesis routes with high yield and low cost to produce MXenes as cathode materials are worthy of further exploration and research. Moreover, ion dynamics and charge storage mechanism among MXene nanosheets need further investigation.
5. Since metal ions possess various valence states in certain MOFs, so they can offer various Lewis acid sites and form chemical interactions of multiple strengths with sulfur and polysulfides. This insight may guide future research in the area of LSBs. Meanwhile, covalent organic frameworks (COFs), novel porous crystalline materials with high porosity, large surface area, high stability, and ready functionality, can potentially be used as host materials. The existence of highly ordered nanopores provides open paths for electrolyte transportation, and wraps elemental sulfur and lithium polysulfides. However, the intrinsically low electronic conductivity of COFs is a limitation for high-rate discharging. Consequently, improving the electronic conductivity and modifying the surface of COFs to promote practical application of LSBs may be the promising research directions.
6. Due to the outstanding processability, flexibility, and broad electrochemical stability window, organosulfur compounds as cathode materials have received extensive attention. Although recent studies on organosulfur cathodes have made great breakthrough in the field of LSBs, the preparation of organosulfur-based cathode materials with higher sulfur loading in large scale is a challenge, which needs to be overcome.
7. A large number of researches are concentrated on experimental efforts. Consequently, the establishment of theoretical models and calculations should be an urgent need for studying LSBs. Theoretical approach plays a very large role in searching, predicting, and guiding the future development of LSBs. Some heteroatoms such as oxygen and nitrogen possess strong affinity with intermediate polysulfides, which has been demonstrated by theoretical calculations.
8. Based on the practical perspective, the electrochemistry and fundamental reaction mechanism of LSBs should be profoundly understood by *in situ* characterization approaches.

Overall, the research on LSBs is still at its early stage, a deeper understanding of the battery system and design of novel cell configurations with higher specific capacity and power density is extremely important, and the profound comprehension of both the fundamental electrochemistry of LSBs and the sulfur redox reaction within the electrode are also crucial. Moreover, investigations on the growth and precipitation of sulfur active species in Li-S system and studies on the effect of volumetric change of elemental sulfur on battery performance during the charge/discharge process are relatively less, which needs to be explored more deeply. Most notably, the cycling stability of lithium metal anode has become a limiting bottleneck in Li-S technology. The commercial application of LSBs is very likely to be realized by the establishment of theoretical models and calculations, advanced host materials, stable solid-state electrolytes, modified lithium metal anodes, and *in situ* characterization approaches. Novel battery configurations, including interlayers and modified separators, are also vital to improve the electrochemical performance of LSBs. All these strategies would make LSBs the most promising candidates for next-generation high-performance lithium batteries.
